# An Unexpected Early Rhabdodontid from Europe (Lower Cretaceous of Salas de los Infantes, Burgos Province, Spain) and a Re-Examination of Basal Iguanodontian Relationships

**DOI:** 10.1371/journal.pone.0156251

**Published:** 2016-06-22

**Authors:** Paul-Emile Dieudonné, Thierry Tortosa, Fidel Torcida Fernández-Baldor, José Ignacio Canudo, Ignacio Díaz-Martínez

**Affiliations:** 1 Grupo Aragosaurus−IUCA, Área de Paleontología, Facultad de Ciencias, Universidad de Zaragoza, Pedro Cerbuna 12, 50009 Zaragoza, Spain; 2 Réserve Naturelle Nationale Sainte-Victoire, Conseil Départemental des Bouches-du-Rhône, 52 avenue de Saint-Just, 13256 Marseille Cedex 20, France; 3 Museo de Dinosaurios de Salas de los Infantes and Colectivo Arqueológico−Paleontológico Salense (CAS), Plaza Jesús Aparicio 9, 09600 Salas de los Infantes, Burgos, Spain; 4 CONICET—Instituto de Investigación en Paleobiología y Geología, Universidad Nacional de Río Negro, General Roca 1242, 8332 Fisque Menuco (General Roca), Río Negro, Argentina; Raymond M. Alf Museum of Paleontology, UNITED STATES

## Abstract

Disarticulated and incomplete remains from a new diminutive ornithopod are described. They come from the Cameros Basin in the north of Spain and were collected from the red clays of the Castrillo de la Reina Formation, ranging from Upper Barremian to Lower Aptian. The new ornithopod described here is slender and one of the smallest ever reported. An up-to-date phylogenetic analysis recovers this taxon as a basal iguanodontian. Its unique combination of characters makes it more derived than slender ornithopods like *Hyphilophodon* and *Gasparinisaura*, and bring very interesting insights into the basal iguanodontian phylogeny. Though possessing a minimum of three premaxillary teeth, this taxon also bears an extensor *ilio-tibialis* groove on the distal part of its femur. Moreover, its dentary and maxillary teeth are unique, remarkably similar to those regarded as having a “rhabdomorphan” affinity. This unknown taxon is suggested to be a stem taxon within Rhabdodontidae, a successful clade of basal iguanodonts from the Late Cretaceous of Europe. The Gondwanan ornithopods share the strongest affinities with this family, and we confirm *Muttaburrasaurus* as a sister taxon of the Rhabdodontidae within a newly defined clade, the Rhabdodontomorpha.

## Introduction

Seeley [1] was the first to recognize two orders within Dinosauria: Saurischia and Ornithischia. Ornithischia is recognized by a typically posteriorly oriented pubis. Within Ornithischia, Romer [2] considered Ornithopoda as a suborder that includes all the relatively unspecialized, bipedal ornithischians. Since then, there has been an increasing awareness that all the main suborders of ornithischians in fact arose from a “paraphyletic plexus” of small, unarmored and bipedal forms, commonly called the “hypsilophodontids” [3]. Nonetheless, concepts of “hypsilophodontid” have changed over time. At one time Sereno [4] considered Hypsilophodontidae to be monophyletic within Ornithopoda, though later it was again considered paraphyletic [5–7]. In its most recent conception, Hypsilophodontidae contains only a single taxon, *Hypsilophodon foxii* [8]. The original sense of the suborder Ornithopoda *sensu* Galton [5] also changed to be more restrictive, notably by eliminating the fabrosaurids and heterodontosaurids [7] until the point that, in the most recent work of Boyd [8], most of the previously thought basal ornithopods were placed outside of Ornithopoda into the new clade Parksosauridae and as sister taxa to Cerapoda. This considerable change results in the only non-iguanodont basal ornithopod being *Hypsilophodon foxii*. Basal iguanodontian are numerous and very diverse, and their origins could date to as early as in the Middle Jurassic [9]. The monophyly of Iguanodontia has also been questioned many times, because some basal iguanodonts share numerous plesiomorphic characters with those animals previously called “hypsilophodontids” [10–13]. Recent discoveries found many “basal iguanodonts” that were not graviportal, as occurs for *Gasparinisaura* [14], *Anabisetia* [15], *Talenkauen* [16] and *Macrogryphosaurus* [17]. These taxa often raised questions about their real systematic position and suggested a deeply nested origin of Iguanodontia within Ornithopoda [7, 15–17]. The most recent phylogeny of Boyd [8] discards the iguanodontian affinities of *Talenkauen* and *Macrogryphosaurus* and set them within the new family Parksosauridae.

Among basal iguanodontians, Rhabdodontidae constitutes a peculiar family endemic to the Late Cretaceous of Europe [18]. This clade is composed of three genera with six species: *Mochlodon* (*M*. *suessi*, *M*. *vorosi*) from Austria and Hungary [18], *Rhabdodon* (*R*. *priscus*, *R*. *septimanicus*) from France and Spain [19–21] and *Zalmoxes* (*Z*. *robustus* and *Z*. *shqiperorum*) from Romania [22, 23]. Historically, the first attempt to place the rhabdodontids into an ornithopod phylogeny was achieved by Pincemaille [24], who created the informal group of ‘rhabdomorpha”. Ruiz-Omeñaca [25] proposed an initial definition of this clade. The family Rhabdodontidae was then created by Weishampel *et al*. [23] and represented a node-based taxon defined as “The most recent common ancestor of *Zalmoxes robustus* and *Rhabdodon priscus* and all the descendants of this common ancestor”. Unfortunately, the family definition suffered from the fragmentary nature and unknown relationships of many of the species associated with the group and the risk of the specifiers falling outside of the traditional clade [26]. A new definition of Rhabdodontidae *sensu* Sereno 2005 [26] corresponds to a stem-based family, defined as the most inclusive clade containing *Rhabdodon priscus* Matheron 1869 [20] but not *Parasaurolophus walkeri* Parks 1922 [27].

Ornithopods from the lowermost Cretaceous (Berriasian–Hauterivian) are poorly known in Europe. During the Barremian, they suddenly appear in greater numbers, extending from England to the Iberian Peninsula. In England, *Hypsilophodon foxii* is one of the best-known and best-documented species among dinosaurs [28]. In Spain, postcranial remains from a new “hypsilophodontid” species have been reported near Igea [29]. A new ornithopod genus and species, *Gideonmantellia amosanjuanae*, has recently been described in the Barremian of Galve [30]. In Spain (e.g. Vallipón [31] and La Solana [25]) and in France (Angeac-Charente [32]), many isolated teeth found from these ages present a morphotype close to that of *Hypsilophodon foxii* [33, 34]. However, some of these isolated teeth clearly stand out as distinct morphotypes, resembling those borne by the so-called “rhabdomorpha” [25]. Upper and lower teeth are spade-like, maxillary teeth have many sub-equal labial ridges and no prominent primary ridge, and dentary teeth bear one very prominent lingual ridge. The Peñascal teeth (Barremian-Aptian of Salas de los Infantes) were classified as “Ornithopoda nov. gen. et sp.”, with a noticeable “rhabdomorphan” affinity [35]. The teeth belonging to the material we describe herein are very similar to the former, and come from a locality named Vegagete, in very close proximity to the Peñascal site. Another interesting Early Cretaceous maxillary crown from the deposits of La Cantalera has been mentioned; it was assigned to Rhabdodontidae? indet. [36]. However, given the current state of knowledge for the “rhabdomorphans” in the Early Cretaceous of Europe, any assignment to this group should be taken very cautiously.

The use of “Rhabdomorpha” as a clade name is problematic because 1) it was never clearly defined; and 2) its name conflicts as a junior synonym with the crustacean *Rhabdomorpha* Fukui, 1965 [37]. Even though this genus is a junior synonym, and now rejected, we have to follow the article 23.3.6 of the International Code of Zoological Nomenclature. It stipulates that the principle of priority continues to be applied to an available name when treated as a junior synonym because it may be available for the case an author considers the synonymy to be erroneous. So, the informal clade name “Rhabdomorpha” must be abandoned.

The Vegagete ornithopod was initially interpreted to belong to “*Hypsilophodon* cf. *foxii*” in a very brief description given by Fuentes Vidarte and Meijide Calvo [38]. Later, these remains were classified as “Ornithopoda indet.” [39]. However, no detailed study has yet been made of this material. The first aim of this paper is to provide a complete anatomical description of all the Vegagete ornithopod remains. We follow with a phylogenetic analysis that includes this taxon. This recovers some surprisingly primitive characters, with others usually found in more derived ornithopods. Some important ontogenetic issues are addressed, based on the different individual sizes found.

## Geographical and Geological Context

**Fig 1:**
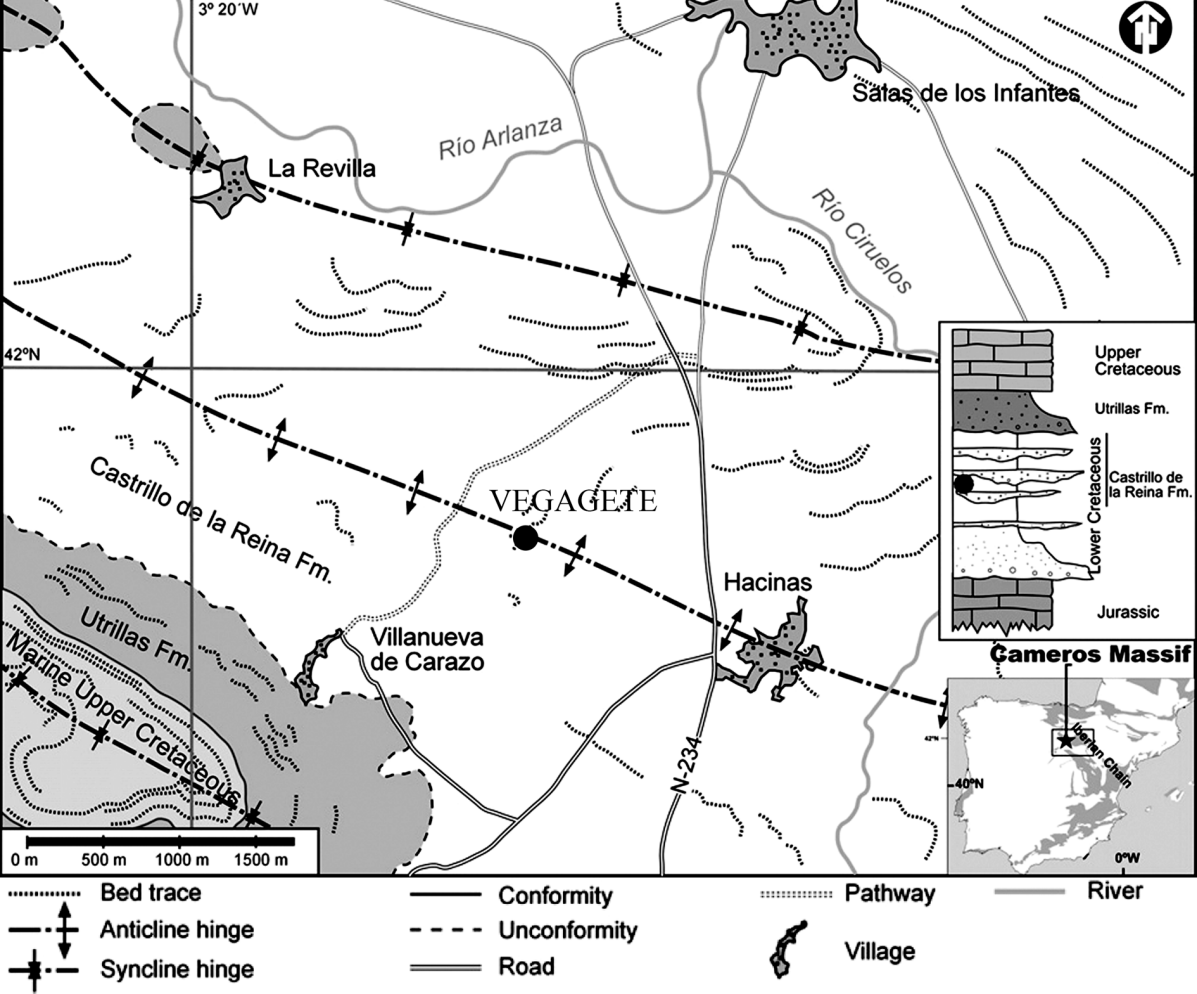
Geographic and geological context surrounding the Salas de los Infantes municipality. The stratigraphic position of the Vegagete deposit is indicated with the dot. It consists principally of floodplain red clays deposits. The geological map has been taken from [44].

The Vegagete dig-site is located in the province of Burgos, between the municipalities of Salas de los Infantes and Villanueva de Carazo (Fig 1). Its geographic coordinates are 4649825N / 30T474828E [38]. The deposit was excavated in 1998, from the surface of a red clay lens extending over an area of roughly three square meters. Vegagete lies in the north-western sector of the Cameros Basin. This basin was infilled above an extensive fault several kilometers deep, between the Tithonian and the Albian, during the avulsion of the rift that opened along the Iberian Range axis [40]. The Cameros sedimentary megasequence is subdivided into five stratigraphic units [41]. The geological formation containing the Vegagete dig site is Castrillo de la Reina, belonging to the Urbión lithostratigraphic group [42]. Despite the lack of any clear magnetostratigraphic discontinuity, the biozonation of this group, based on charophytes and ostracods, gives a late Barremian to early Aptian age [42, 43]. It is characterized by alternating banks of white fluviatile sandstones with floodplain red clay deposits. The red clay yielded the ornithopod remains under study in this paper. This formation has already produced a number of terrestrial vertebrate fossil remains, such as the varanoid *Arcanosaurus ibericus* [44] and the rebbachisaurid sauropod *Demandasaurus darwini* [45].

## Material and Methods

All of the material described is curated at the Dinosaur Museum of Salas de los Infantes—MDS (Salas de los Infantes, Burgos, Spain). This material was obtained from two distinct sources. Part of it was given by the cultural association “Colectivo Arqueológico y Paleontológico Salense” to the town hall of Salas de los Infantes for curation at the Dinosaur Museum. Note that the MDS is legally integrated into the Museum system of the Castilla y León Autonomous Community (Order CYT/1210/2007, from June 15, 2007). The second part of the material was retrieved during a prospecting campaign that aimed to inventory the different paleontological deposits from the Salas de los Infantes region. This latter campaign was promoted by the Heritage General direction of the Castilla y León Autnomous Community (number of document 05/020-BU, Section: Research and development JDVR/MCP). Authorization to access the material was given by Fidel Torcida, the Museum conservator.

The material under study is composed of numerous fragments, numbered from MDS-VG,1 to MDS-VG,280, and many of the fragments used in the description do not have an inventory number. The collection represents at least five fragmentary individuals from a single ornithopod taxon. This estimation was achieved by counting left and right distal fragments of metatarsal II. Three ontogenetic stages stand out, based on size differences for similar bones. These are distributed as follows: one very small (ontogenetic stage 1), three medium sized (ontogenetic stage 2), and one bigger individual (ontogenetic stage 3). The latter individual was estimated at 65 to 70 cm in body length [38]. No detailed taphonomical study could be made efficiently at Vegagete, because of the very small proportions of the dig site, a sedimentary lens no deeper than 5 cm, and no larger than 3 m^2^, as well as the very small size of the material and its apparent disarticulation at the surface. Despite this, we can make the following statements. The assemblage is monospecific. Most of the material is fractured and preserves only the extremities of the long bones, except for some pedal phalanges. We note that the material is in a few cases embedded in a dolomitic matrix. In many cases, even the tiniest bones preserve a smooth and intact bony surface. We attribute the fragmentary nature of the material both to post-mortem diagenetic breakage under short distance transport as well as to the original lack of bone ossification. The death of the five individuals simultaneously is likely to have been due to a sudden inundation, which carried and buried the cadavers a relatively short distance away. The finding of these different-sized individuals altogether suggests they were living in a herd.

The following bones are described here: three fragments of dentaries (MDS-VG,7, 8, 16/17/152), one fragment of a premaxilla (MDS.VG, unlabelled) and another of a maxilla (MDS-VG,9), one premaxillary tooth (MDS-VG,3), three maxillary teeth (MDS-VG,9, 35, 37), four dentary teeth (MDS-VG,33, 34, 42, 16/17/152), a laterosphenoid (MDS.VG, unlabelled), three cervical vertebrae (MDS-VG,50, 53, 56), seven dorsal vertebrae (MDS-VG,57, 59, 64, 66, 67, 69, 75), three sacral vertebrae (MDS-VG,77, 79, 81), eight caudal vertebrae (MDS-VG,55, 72, 86, 87, 95, 100, 101, 102), one proximal fragment of a scapula (MDS.VG, unlabelled), one coracoid (MDS.VG, unlabelled), one proximal fragment of humerus (MDS-VG,113), one distal fragment of humerus (MDS-VG,168), one proximal fragment of ulna (MDS-VG,202), one distal fragment of ulna (MDS.VG, unlabelled), one partial ilium (MDS.VG, unlabelled), three proximal femora fragments (MDS-VG,108, 109, 159), one femoral diaphysis (MDS-VG,122), three distal femora fragments (MDS-VG,132, 134, 135), three proximal tibia fragments (MDS-VG,136, 137, and unlabelled), one distal tibia fragment (MDS-VG,140), one proximal fibula fragment (MDS-VG,107), one distal fibula fragment (MDS-VG,199), and one foot recomposed with the help of the distal first, proximal and distal second, third and fourth metatarsals (MDS-VG,171, 177, 160, 174, 163, 178, 169 respectively), the phalanges of digit I (MDS-VG,239), digit II (MDS-VG,232, 210, 233), digit III (MDS-VG,209, 237, 254, 245), digit IV (MDS-VG,211, 215, 243, 240, 247), and the claws from digit I, II, III and IV (MDS-VG,266, 262, 272, 258 respectively). All of these bones are listed in Table 1, with their given size-related ontogenetic category and their respective measurements.

**Table 1:** List of the bones referred in the text, followed with their respective measurements and detailed information. Abbreviations: APPEND., appendicular skeleton; ONTO., ontogenetical stage; j., juvenile; subad., subadult; ad., adult; F., fused centra; nF., non-fused centra; MDS.VG, inventory number for the Vegagete specimen; N.I., non-inventoried; Fr. details, if fragmentary: fragment location onto the bone; L, length; W, width; H, height; (ant.), anterior; (post.), posterior; (prox.), proximal; (dist.), distal; NA, non-applicable. Measures are in millimeters. N.B.1: Teeth measurements are exclusively done on their crowns. N.B.2: vertebrae measurements are exclusively done on their centra.

CRANIUM	**ONTO.**	**MDS. VG**	**Fr. details**	**L**	**W**	**H**		
Premaxillary	?	N.I.	?	NA	?	NA		
Maxillary	?	9	Posterior	NA	?	NA		
Dentary	?	7	Anterior	NA	?	NA		
Dentary	?	8	-	NA	?	NA		
Dentary	?	16/17/152	Posterior	NA	?	NA		
Pmx. tooth	?	3	Broken	NA	NA	NA		
Maxillary tooth	?	9	-	3.2	1.5	2.9		
Maxillary tooth	?	35	-	3.3	1.9	3.6		
Maxillary tooth	?	37	-	2.8	1.6	3.2		
Dentary tooth	?	16	-	2.7	1.4	2.2		
Dentary tooth	?	33	-	4.5	2.2	3.9		
Dentary tooth	?	34	-	3.1	1.9	3.9		
Dentary tooth	?	42	-	2.7	1.8	3.7		
Laterosphenoïd	?	N.I.	-	10.7	5.6	5.9		
								
AXIAL	**ONTO.**	**MDS.VG**	**Details**	**L**	**W (ant.)**	**H (ant.)**	**W (post.)**	**H (post.)**
Dorsal vertebra	1. nF.	64	Anterior	7.4	5.1	4.6	5.3	4.8
Dorsal vertebra	1. nF.	75	Anterior	7.9	5.6	5.4	6.0	4.0
Dorsal vertebra	1. nF.	57	Mid-series	8.7	6.3	5.3	6.3	5.6
Dorsal vertebra	1. F.	67	Posterior	9.2	7.4	6.1	7.0	5.7
Dorso-sacral v.	1. F.	79	Dorso-sacral	8.4	5.9	6.2	7.3	5.2
Sacral vertebra	1. F.	81	First sacral	8.7	8.0	5.0	7.8	4.7
Caudal vertebra	1. nF.	87	Anterior	10.0	6.6	6.6	6.1	6.6
Caudal vertebra	1. nF.	95	Anterior	8.9	6.0	6.2	5.9	6.7
Caudal vertebra	1? F.	55	Posterior	7.2	3.8	3.4	2.2	1.0
Cervical vertebra	2? F.	53	Anterior	9.7	7.6	4.5	6.1	5.1
Cervical vertebra	2? nF.	56	Mid-series	8.7	6.2	4.7	6.2	4.5
Cervical vertebra	2? nF.	50	Posterior	8.2	5.7	4.9	5.2	5.0
Dorsal vertebra	2? nF.	59	Anterior	8.2	5.9	5.3	6.1	5.0
Dorsal vertebra	3. F.	66	Mid-series	10.9	7.8	6.2	7.8	7.2
Dorsal vertebra	3. F.	69	Posterior	11.2	9.3	7.7	8.8	8.3
Sacral vertebra	3. F.	77	Posterior	11.0	7.4	8.1	7.4	6.8
Caudal vertebra	3. F.	72	Anterior	11.0	8.4	7.8	8.0	6.8
Caudal vertebra	3. F.	101	Anterior	10.8	5.7	5.6	5.3	5.4
Caudal vertebra	3. F.	86	Mid-series	10.5	6.1	6.5	6.5	6.6
Caudal vertebra	3. F.	102	Mid-series	10.9	4.8	6.3	5.0	6.3
Caudal vertebra	3. F.	100	Posterior	12.8	5.7	5.6	5.3	5.4
APPEND.	**ONTO.**	**MDS.VG**	**Fr. details**	**L**	**W (prox.)**	**H (prox.)**	**W (dist.)**	**H (dist.)**
Left scapula	3.	N.I.	Post-distal	NA	NA	NA	5.4	NA
Right humerus	3.	113	Proximal	NA	10.9	NA	NA	NA
Right humerus	2?	168	Distal	NA	NA	NA	8.1	5.8
Left ulna	2?	202	Proximal	NA	5.3	7.8	NA	NA
Left ulna	2?	N.I.	Distal	NA	NA	NA	6.8	3.2
Ilium (left)	1.	N.I.	-	NA	NA	NA	3.4	NA
Femur (right)	1.	159	Proximal	NA	12.3	10.6	NA	NA
Femur	2.	108	Proximal	NA	15.8	NA	NA	NA
Femur (right)	3.	109	Proximal	NA	17.5	NA	NA	NA
Femur (right)	2?	122	Diaphysis	NA	NA	NA	NA	NA
Femur (left)	2.	132	Distal	NA	NA	NA	NA	?
Femur (left)	2.	134	Distal	NA	NA	NA	15.1	11.9
Femur (right)	3.	135	Distal	NA	NA	NA	17.6	NA
Tibia (left)	2.	137	Proximal	NA	7.0	4.3	NA	NA
Tibia (left)	2.	N.I.	Proximal	NA	8.4	NA	NA	NA
Tibia (left)	2.	136	Prox., crushed	NA	8.6	16.7	NA	NA
Tibia (right)	3.	140	Distal	NA	NA	NA	18.5	8.4
Fibula (left)	2.	107	Proximal	NA	NA	NA	NA	NA
Metatarsal I (left)	3.	171	Distal	NA	NA	NA	5.6	4.7
Metatarsal II (left)	2.	177	Proximal	NA	6.6	NA	NA	NA
Metatarsal II (left)	2.	160	Distal	NA	NA	5.2	5.3	NA
Metatarsal III (left)	2.	174	Proximal	NA	4.9	7.6	NA	NA
Metatarsal III (left)	2.	163	Distal	NA	NA	NA	6.8	5.1
Metatarsal IV (left)	2.	178	Proximal	NA	5.5	5.8	NA	NA
Metatarsal IV (left)	2.	169	Distal	NA	NA	NA	5.6	5.6
Phalanx I-1 (left)	2.	239	Distal	NA	NA	NA	4.0	3.4
Claw I (left)	3.	266	Proximal	NA	4.7	3.6	NA	NA
Phalanx II-1 (left)	2.	232	Proximal	NA	5.6	6.1	NA	NA
Phalanx II-1 (right)	2.	210	Distal	NA	NA	NA	5.0	5.1
Phalanx II-2 (left)	2.	233	Distal	NA	NA	NA	4.1	?
Claw II (left)	2.	262	Complete	?	3.6	3.6	NA	NA
Phalanx III-1 (left)	2.	209	Complete	14.2	8.4	5.6	6.7	5.0
PhalanxIII-2(right)	2.	237	Proximal	NA	6.6	NA	NA	NA
Phalanx III-2 (?)	2.	254	Distal	NA	NA	NA	5.8	?
Phalanx III-3 (left)	2.	245	Complete	8.2	5.7	?	4.5	?
Claw III (left)	2.	272	≈ Complete	≈ 9.0	4.6	3.9	NA	NA
PhalanxIV-1(right)	2.	211	Proximal	NA	6.1	6.0	NA	NA
PhalanxIV-1(right)	2.	215	Distal	NA	NA	NA	6.1	?
Phalanx IV-2 (left)	2.	243	Complete	8.2	5.4	?	5.1	?
Phalanx IV-3 (left)	2.	240	Complete	7.2	4.8	?	4.2	?
Phalanx IV-4 (left)	2.	247	Complete	5.7	4.4	4.6	3.9	?
Claw IV (right)	3.	258	Proximal	NA	4.9	4.4	NA	NA

## Systematic Paleontology

DINOSAURIA Owen, 1842 [46]

ORNITHISCHIA Seeley, 1887 [1]

NEORNITHISCHIA Cooper, 1985 [47]

CERAPODA Sereno, 1986 [4]

ORNITHOPODA Marsh, 1881[48] *sensu* Butler *et al*. 2008 [7]

IGUANODONTIA Dollo, 1888 [49]

RHABDODONTOMORPHA nov.

**Etymology:** From the genus of the first representative of this clade *Rhabdodon priscus* [20] and “-morpha” the suffix indicating an ancient variant or morph for this clade.

**Phylogenetic definition:** Rhabdodontomorpha is phylogenetically defined as a node-based taxon consisting of the most inclusive clade containing *Rhabdodon priscus* Matheron, 1869 [22] and *Muttaburrasaurus langdoni* Bartholomai and Molnar, 1981 [50]. Rhabdodontomorpha currently includes *Mochlodon suessi* [51], *M*. *vorosi* [18], *Muttaburrasaurus langdoni* [50], *Rhabdodon priscus* [20], *R*. *septimanicus* [52], *Zalmoxes robustus* [22] and *Z*. *shqiperorum* [22].

**Diagnosis:** Rhabdodontomorpha is defined by the combination of the following synapomorphies (see phylogenetic analysis): 1) the maxillary process of the jugal is subrectangular and overlaps the maxilla with parallel dorsal and ventral margins, 2) the humerus shaft is strongly bowed from an anteroposterior view, 3) the ilium has a lateral deflection of the preacetabular process equaling or exceeding 30°, 4) the ilium has a dorsal margin of the preacetabular process transversely expanded to form a narrow shelf, 5) the dorsal margin of the ilium is mediolaterally thickened at the level above its ischiac peduncle, 6) the femur has a shallow and non-constricted trochanteris fossa on its proximal articular surface (not present in the more derived taxa *Zalmoxes* and *Rhabdodon*).

RHABDODONTIDAE Weishampel, Jianu, Csiki & Norman, 2003 [22] *sensu* Sereno, 2005 [26]

**Emended diagnosis:** Rhabdodontidae is defined by the combination of the following synapomorphies (see phylogenetic analysis): 1) a humerus with a flat proximal anterior surface, i.e. devoid of any bicipital sulcus, 2) a humerus with a concave lateral border between the head and the deltopectoral crest in anteroposterior view, 3) an ulna with a relatively large olecranon process. A potential apomorphy would be a femur with a crest-like, non-pendant fourth trochanter.

Gen. et sp. indet.

## Description

### Skull

#### Premaxilla

**Fig 2:**
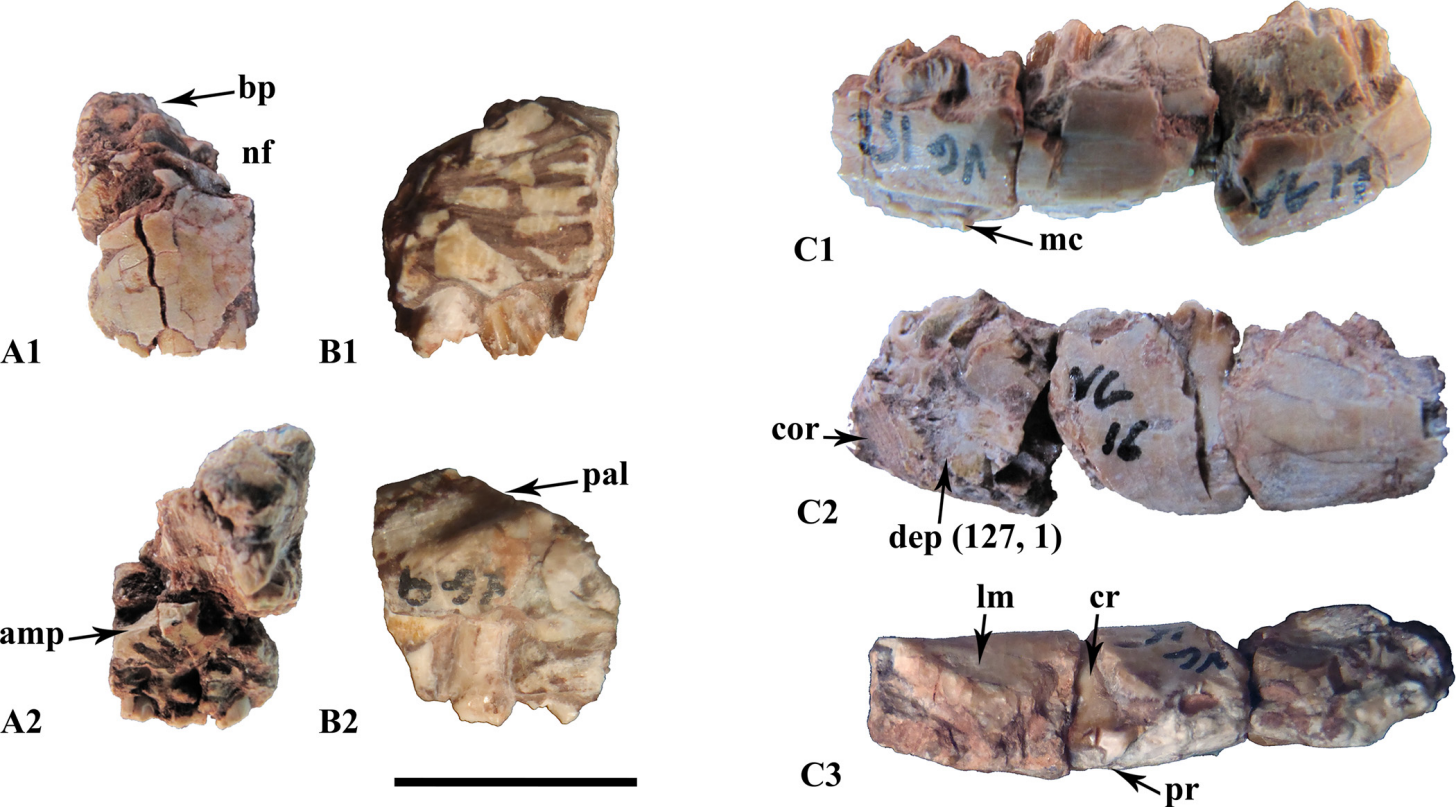
Snout elements from the Vegagete taxon. (A) premaxillary fragment (non-inventoried) in labial (A_1_) and medial (A_2_) views; (B) posterior maxillary fragment (MDS-VG,9) in labial (B_1_) and lingual (B_2_) views; (C) posterior dentary fragment (MDS-VG,16/17/152) in lingual (C_1_), labial (C_2_) and occlusal (C_3_) views. Abbreviations: amp, anteromedial maxillary process; bp, bony process; mc, Meckelian canal; cor, coronoid insertion area; cr, curved root; dep, depression for the adductor jaw musculature; lm, labial emargination; nf, narial fossa; pal, palatin insertion area; pr, mesially bent primary ridge. Scale: 1cm.

One left premaxilla fragment was found (still unnumbered) and preserves a tooth row consisting of three hollow roots. As is usually found in premaxillae, there is neither a labial nor lingual emargination of the tooth row. The lateral margins are not everted, but are completely vertical toward the tooth row. Dorsally, a massive and unrecognized bony process seems to be either stuck to, or part of, the premaxilla (Fig 2). The postero-dorsal portion of the premaxilla displays part of the nasal fossa (Fig 2A_1_). The postero-medial side presents a diagonal, narrow horizontal groove which twists to the vertical more anteriorly and may have served for the insertion of the anteromedial maxillary process (Fig 2A_2_).

#### Maxilla

One fragmentary maxilla (MDS-VG,9) was found. It preserves three teeth that are arranged “*en échelon*”, one behind the other, with the distal side of each tooth labially affixed. On the bone itself, a curious notch, located on the lingual side rises in a posterodorsal direction. This would have articulated with the palatine (Fig 2B_2_). As a consequence, this fragment should belong to the posterior part of the maxilla. The labial surface is marked by tight, sub-parallel ligament insertions.

#### Laterosphenoid

**Fig 3:**
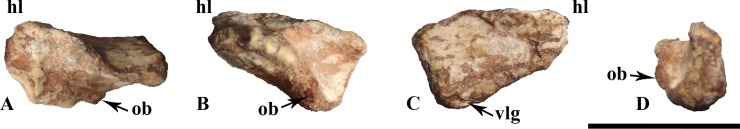
Right laterosphenoid in dorsal (A), medial (B), lateral (C) and posterior (D) views. Abbreviations: hl, anterior head of laterosphenoid; ob, orbitosphenoid boss; vlg, ventral laterosphenoid groove for the *ramus ophtalmicus* (CN IV). Scale: 1cm.

The laterosphenoid is an anteroposteriorly elongate bone located behind the orbit, which forms the junction with the prootic, supraoccipital, parietal, frontal and postorbital. Its posterior margin is straight, and it bends ventrally to contact both the prootic and supra-occipital. At its posterior extremity, the shape of the bone is roughly that of a quarter of cylinder that encloses the ventral part of the parietal (Fig 3D). The posterior basal floor is thick and straight. Interestingly, the presence of the oculomotor nerve foramen (CN III) can be observed, running longitudinally on the ventral side of the posterior part (Fig 3C). The floor of the laterosphenoid is bordered and wrapped laterally by a vertically rising wall, which remains dorsally horizontal all along the bone, thus being higher posteriorly than anteriorly. The basal floor is bent upward anteriorly until the point where it is level with the horizontal lateral wall. An orbitosphenoid boss is located along the ascending medial margin (Fig 3A and 3D). The passage for the trochlear nerve (CN IV) is located immediately anterior to this boss. Anteriorly and at the top of the basal floor, the bone forms a dorsal horizontal shelf, or “head”, constituted by a single, thin sheet of bone. A lip bends ventrally again for a very brief distance more anteriorly. The dorsal surface of the head would probably make contact medially with the posterolateral end of the frontal. The head spreads laterally to contact the medial process of the postorbital. The trigeminal, or prootic foramen, is not observed to notch the posterior end of the laterosphenoid.

#### Dentary

All dentary fragments are slightly curved, concavo-convex dorsoventrally (Fig 2C_1,2_). MDS-VG,7 is an anterior dentary fragment, though its anteriormost portion is not preserved. The Meckelian canal narrows slightly before it ends abruptly anteriorly. From a dorsal view, this dentary has almost no labial emargination, and it is curved with a convex lingual side and a concave labial side. On MDS-VG,8, three neurovascular foramina are aligned labially, equidistantly one behind the other, along a parapet that overhangs a strong ventrolateral convexity. This convexity probably represents the beginning of a more posterior labial emargination. MDS-VG,16/17/152 is the most complete and represents a posterior fragment of a right dentary. Its Meckelian canal is broken in its anterior portion (Fig 2C_1_). Postero-labially, a slightly striated, posteriorly climbing diagonal groove may have served as the anteriormost part of the coronoid insertion area (Fig 2C_2_). Immediately anterior to this groove appears a well-marked depression that may have served as an extended insertion for the external jaw adductor musculature (Ösi *et al*. 2012). In dorsal view, the mandible is smoothly convex lingually, and straight labially. The tooth row curves lingually at mid-length so that a strong labial emargination is created (Fig 2C_3_). The labial emargination disappears again completely posteriorly.

### Teeth

#### Premaxillary teeth

**Fig 4:**
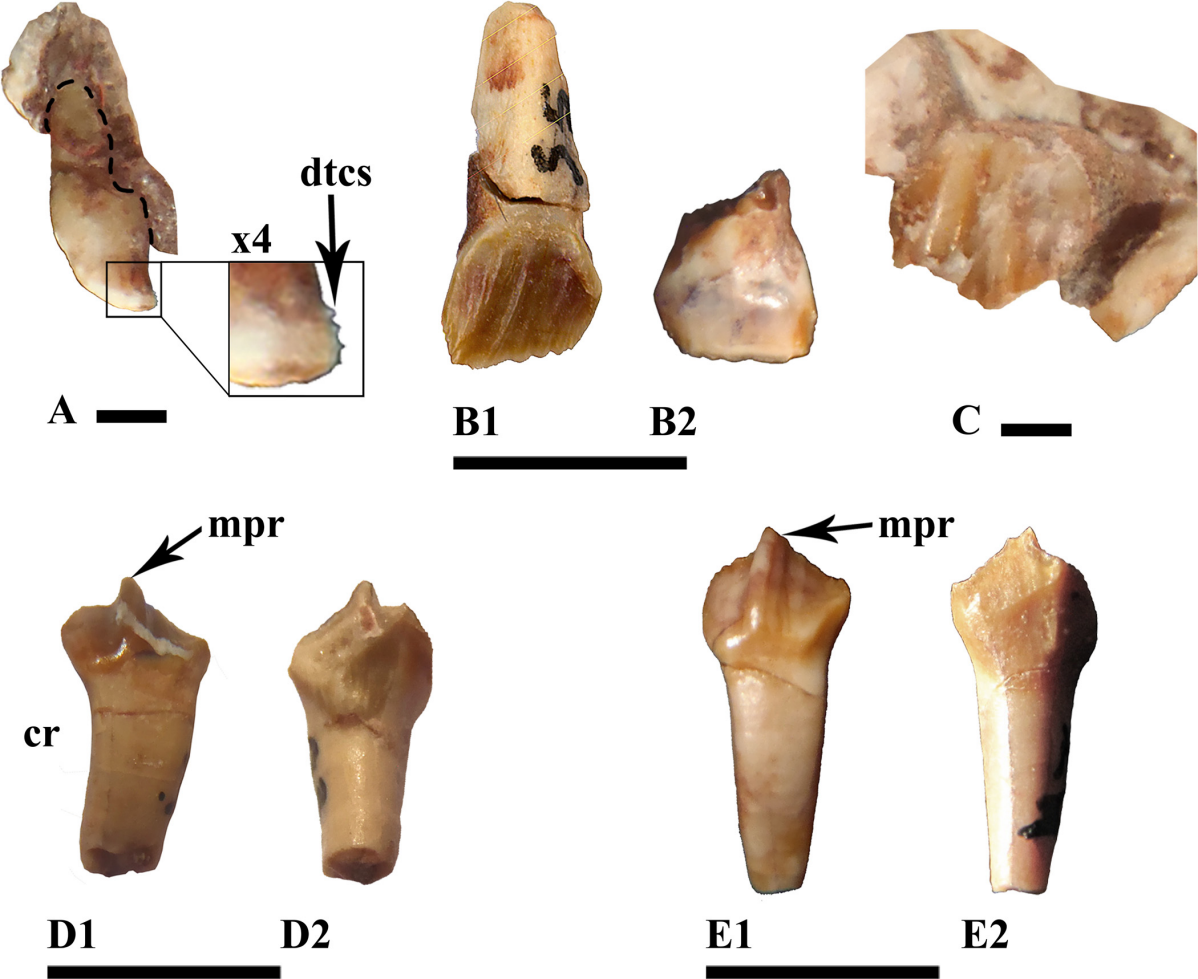
Premaxillary, maxillary and dentary teeth. (A) premaxillary tooth in distal view, (B) standard right maxillary tooth in labial (B_1_) and lingual (B_2_) views, (C) one of the posteriormost left maxillary teeth in labial view, (D) and (E) right dentary teeth in lingual (D_1_, E_1_) and labial (D_2_, E_2_) views. Specimen numbers are MDS-VG,3 (A), MDS-VG,35 (B), MDS-VG,9 (C), MDS-VG,33 (D), MDS-VG,34 (E). Abbreviations: cr, curved root; dtcs, denticles; mpr, mesially bent primary ridge. Scales: 5mm (B, D, E) and 1mm (A, C).

The premaxillary teeth are small and fragile, and their pulp cavities may have reached as far as the base of the crown. The only premaxillary crown found (MDS-VG,3) was studied and photographed, though it later broke off accidentally. The transition between the root and the crown takes the form of a very short and shallow incision (Fig 4A). The crown preserves a circular section, with dimensions identical to the root. It shrinks abruptly at mid-height on one–either labial or lingual–side, producing a sort of “wedge”. The other side remains vertical. Occlusally, the wedged side of the crown flattens and ends up curving as a small tip onto the opposite labiolingual side. An enlarged view of the apicodistal side enables us to see very small emerging denticles (Fig 4A). This tooth is very small, but whether this tooth belonged to a ontogenetic stage 1, 2 or 3 could not be determined with certainty. We cannot reject the possibility that the characteristics of this tooth are variable ontogenetically.

#### Maxillary teeth

The labial sides of the maxillary crowns are ornamented and more strongly enameled. The crown is spatulate. The base of the crown is slightly mesiodistally compressed, being more compressed mesially than distally (Fig 4B). The mesial side is thickened at its base and presents a replacement groove, as also occurs in *Zalmoxes* [22]. In contrast, the base of the crown is thinner distally. The cingulum rises on the mesial side. Jointly, the mesial side is lowest and the most worn, so that apically it is much sharper. The central ridge is as prominent as the secondary ridges. It is observable because it always reaches the cingulum, and bends distally towards the apex. Secondary ridges rise above the first third of the crown’s height, and undulate together in a subparallel way, though this latter character could vary depending on the crown considered. An erupting maxillary tooth crown (MDS-VG,9) reveals that every apical denticle extends into secondary ridges. The sharp, vertical mesiodistal borders bear denticles that extend into shorter tertiary ridges. The maximum number of ridges, including the central one, may be up to ten. Only one maxillary tooth preserves its root (MDS-VG,35). In this specimen, the maxillary crown is low and reaches a height of 0.46 times the total height of the tooth. The root is hollow and not curved (Fig 4B).

Of the three *in situ* maxillary teeth found on the fragment MDS-VG,9, the morphology and ornamentation of the second, the best-preserved and most diagnosable crown (Figs 2B_1_ and 4C), differs substantially from those of the standard isolated maxillary crowns found in this material (Fig 4B). This crown displays a posteriorly shifted prominent central ridge, together with another prominent, more mesial ridge (Fig 4C). These two ridges are indented into further smaller ridges toward the occlusal rim. The distal side is eroded. The relative prominence of these two ridges is very similar to the base of the crown, and this maxillary tooth crown morphology resembles that of *Hypsilophodon foxii* [34]. However, because of its similar overall proportions to the other maxillary teeth and a mostly enameled labial side, for the sake of consistency, MDS-VG,9 is assumed to belong to the same taxon. This specimen provides rare evidence of heterodonty in an ornithopod. Note that a similar issue has already been discussed in the case of the ornithopod *Anabisetia saldiviai* [53].

#### Dentary teeth

The enamel layer is more developed on the ornamented lingual side. This side presents a prominent, mesially inclined central ridge (Fig 2C_3_). The cingulum rises on the posterior side of the tooth. A minimum of three secondary ridges are observed; the first is mesial, the second rises onto the distal flank of the central primary ridge, and the third one is distal. Each secondary ridge is prolonged as a denticle. There are many other denticles, which are the prolongation of smaller tertiary ridges. These denticles are gathered occlusally along the mesial and distal margins of the crown. Some of them are observed in pairs in a single dentary tooth (MDS-VG,42). With respect to the central primary ridge, the distal portion of the crown is usually more worn and lower in height than the mesial portion of the crown (Fig 2D_2_ and 2E_2_). A maximum of six denticles has been counted on the mesial margin. Labially and below the wear surface, the crown shows in some cases some very smooth, inconspicuous ridges (Fig 4D_2_). The root is hollow and curved, being concavo-convex labiolingually. The observed degree of curvature for the dentary tooth roots is highly variable. For instance, it is strong in MDS-VG, 33 and MDS-VG,16/17/152 (Figs 4D and 2C_3_), but it is weaker in MDS-VG,34 (Fig 4E). The root curvature would have varied gradually depending on the position which the tooth would have occupied along the mandible. Most probably, the greatest curvature would have been acquired where the mandible had the widest labial emargination, (i.e. posteriorly here). The crown is slightly smaller than the root, and has a ratio of 0.48 with respect to the total height of the tooth (cf. MDS-VG,33 and 34).

### Axial skeleton

#### Cervical vertebrae

**Fig 5:**
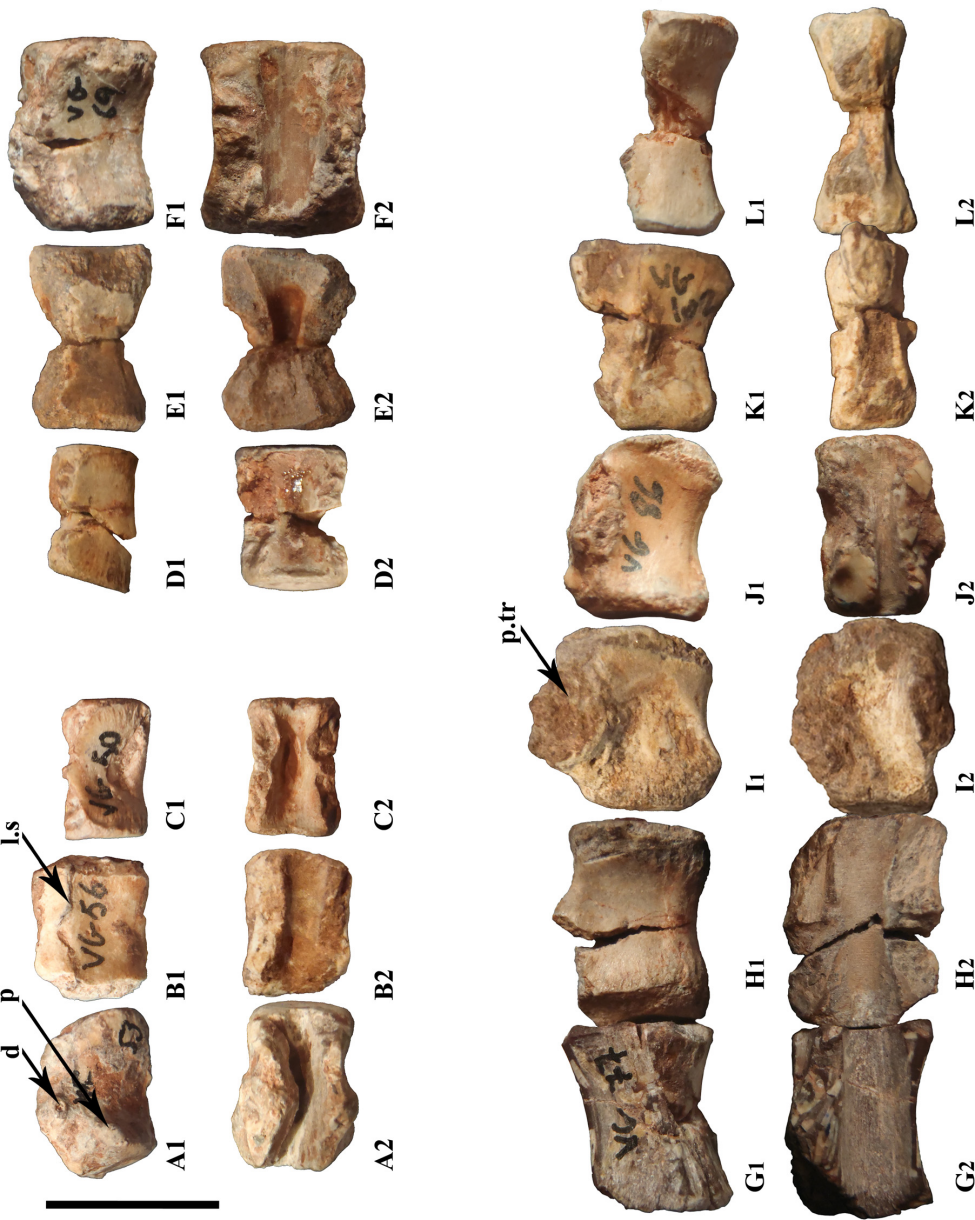
Vertebral column reconstruction of the Vegagete ornithopod using most representative vertebrae. Cervical (A-C); dorsal (D-F); one posterior sacral and caudal vertebrae (G-L) are represented. These are the lateral (A_1_-M_1_) and dorsal views (A_2_-M_2_) of the same vertebrae. Their identifying number are respectively MDS-VG,53 (A), -56 (B), -50 (C), -59 (D), -66 (E), -69 (F), -77 (G), -72 (H), -101 (I), -86 (J), -102 (K), -100 (L). Abbreviations: d, diapophysis; l.s, suture line; p, parapophysis; p.tr, transversal process. Scale:1 cm. N.B.: all vertebrae probably belong to ontogenetic stage 2 and/or 3, except for (A-D) which could belong either to ontogenetic stage 1 and/or 2.

The cervical centra are characterized by their anterodorsally located parapophyses. These centra are rectangular in lateral outline and are more elongated than the other centra (Fig 5A_1_–5C_1_). They are biconcave laterally. Their ventral surface forms a straight ventral keel that is more or less sharp depending on the specimen. All of the cervical centra are amphiplatyan to slightly amphicoelous. MDS-VG,53 corresponds to an anterior cervical vertebra (maybe the third cervical centrum). The anterior articular surface is pentagonal, whereas the posterior surface is heart-shaped. The ventral surface is wedge-like and one of the widest. MDS-VG,56 is from a more posterior position than MDS-VG,53. Its two lateral edges are more vertical; the centrum is narrower. The neurocentral suture remains well visible, so the neural arch may have been partially fused to the centrum. It produces a clearly visible bulge anteriorly, as occurs in *Hypsilophodon foxii* [28]. In dorsal view, the neural canals of MDS-VG,56 and MDS-VG,50 remain shallow all along and widen substantially anteriorly (Fig 5B_2_ and 5C_2_). MDS-VG,50 would be from a more posterior position than MDS-VG,56. The ventral ridge of the former is thinner, and more rounded as well, as also occurs in anterior dorsal vertebrae. Its posterior articular surface is heart-shaped.

#### Dorsal vertebrae

As in *Hypsilophodon foxii* [28], all of the dorsal vertebrae are slightly amphicoelous. Their ventral surfaces are rounded. Three additional features vary continuously along the dorsal column (Fig 5D–5F). Firstly, the dorsal centra display a distinct lateral compression at their mid-length, which results in a butterfly-like dorsal outline. Then, the neural canal deepens inside the centrum, to varying degrees, midway in the anteroposterior direction. Finally, the ventral surfaces of the dorsal centra are variably concave in lateral view. All three features are exaggerated in the middle of the trunk (e.g. MDS-VG,66), but they are all weaker in more anterior (e.g. MDS-VG,59) or posterior (e.g. MDS-VG,69) positions. In MDS-VG,59, a thin and straight ventral keel is preserved, as in cervical vertebrae, but this disappears in more posterior dorsal vertebrae. At mid-length, the neural canal makes a shallow incision, and the centrum narrows only moderately mediolaterally. The posterior dorsal centrum MDS-VG,69 keeps a slightly concave ventral surface; its neural canal no longer shows the typical butterfly-like outline. The neural canal is relatively shallow.

#### Sacral vertebrae

The only dorsosacral vertebra found is from the smallest individual (MDS-VG,79). Its ventral surface is smoothly concave in lateral view, as for the posterior dorsal vertebrae. The neural canal is shallow, but curiously narrows suddenly at mid-length, being restricted to a thin strip posteriorly. Other sacral vertebral centra may be recognized by their flat ventral surfaces. Their neural canals remain shallow and are rectangular in dorsal view (Fig 5G and 5H). There are some lateral insertion surfaces for the sacral ribs, but these are not yet fused. The typical anterior sacral centrum is wide and dorsoventrally low (e.g. MDS-VG,81). More posterior sacral vertebrae are taller, and more contracted laterally (e.g. MDS-VG,77). The same arrangement was described in *Gideonmantellia amosanjuanae* by Ruiz-Omeñaca *et al*. [30].

#### Caudal vertebrae

MDS-VG,72 is considered the anteriormost caudal centrum, on the basis of a rounded and slightly concave ventral surface that differs from the distinctly flat and straight ventral surface of the sacral vertebrae. However, it is noteworthy that MDS-VG,72 is devoid of any chevron articular facets. The broken transverse processes are visible at their base and arise from slightly above the neurocentral suture. The second caudal centrum is MDS-VG,101, with transverse processes being angled steeply upward. No suture line can be made out, but these transverse processes may have risen slightly above the neurocentral suture line as well. MDS-VG,101 bears chevron articular facets posteriorly. These are almost not visible anteriorly. MDS-VG,72 and MDS-VG,101 are considered slightly opisthocoelous, in that they have a flat anterior and a concave posterior articular surface. They are shorter than the more anterior sacral vertebrae (Fig 5H and 5I). All of the other more posterior centra are amphicoelous and bear transverse processes on their neurocentral suture line. At a certain point, the caudal centra start increasing in length and narrowing lateromedially (Fig 5J and 5K). The mid-tail series bears the most concave ventral surfaces. Their transverse processes become restricted to a single mass. Finally, the transverse processes completely disappear and the heights of the centra start decreasing (MDS-VG,100; Fig 5L). Only at this point in the series do the chevron articular facets disappear (MDS-VG,55, unfigured). The last caudal vertebrae, MDS-VG,100 and MDS-VG,55, stand out in that they present a much flatter ventral surface than any of the other caudals.

### Forelimb

#### Scapula

**Fig 6:**
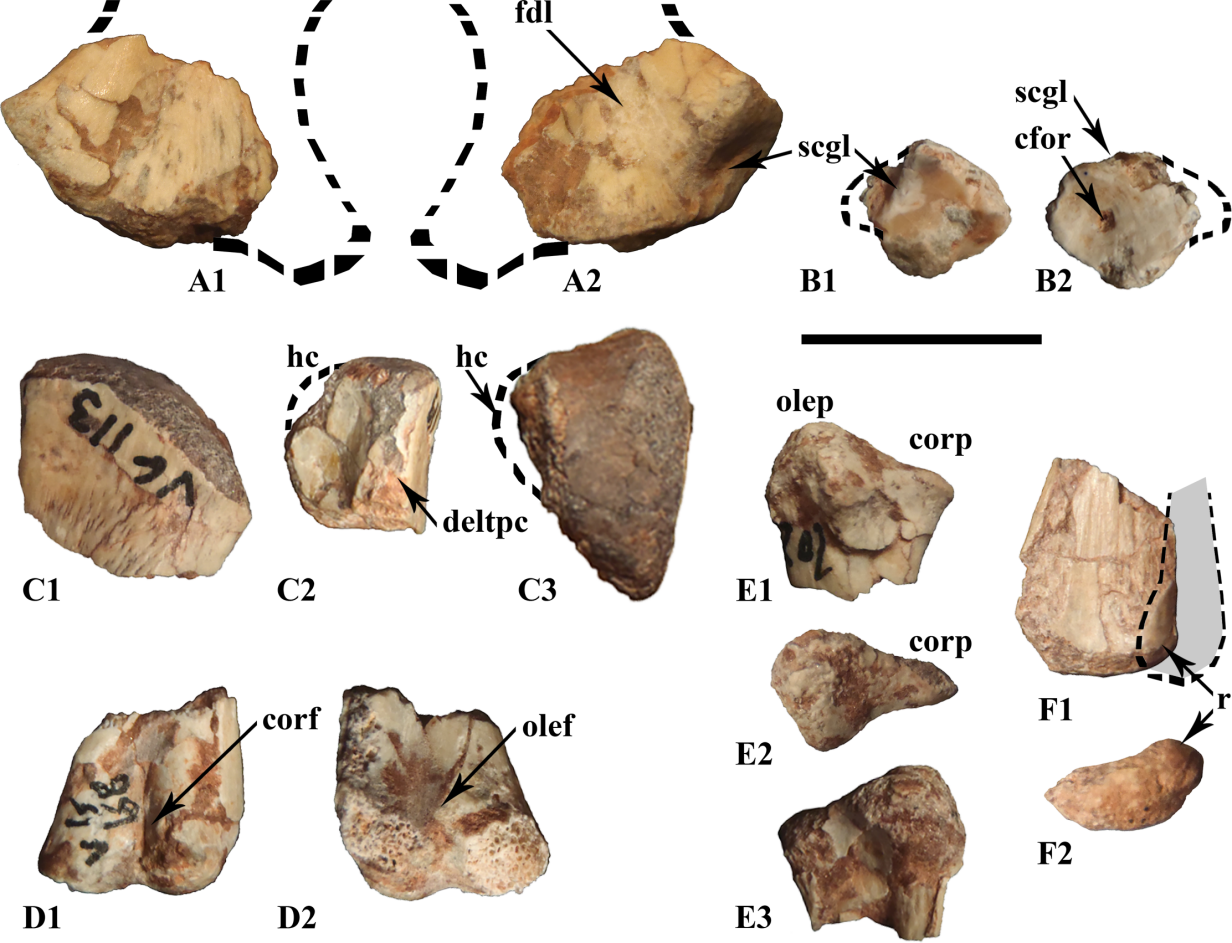
Forearm bones of the Vegagete ornithopod. Left posteroproximal fragment of scapula in medial (A_1_) and lateral (A_2_) views; left coracoid in medial (B_1_) and lateral (B_2_) views; right proximal articular head fragment of humerus in anterior (C_1_) lateral (C_2_) and proximal (C_3_) views; left distal humerus in anterior (D_1_) and posterior (D_2_) views; right proximal ulna in lateral (E_1_), proximal (E_2_) and medial (E_3_) views; left distal ulna in anterior view, with hypothetical reconstruction of the distal contact with the radius (F_1_) and same ulna in distal view (F_2_). Specimens: unnumbered (A, B, F); MDS-VG,113 (C), MDS-VG,168 (D), MDS-VG,202 (E). Abbreviations: cfor, coracoidian foramen; corf, coronoid fossa; corp, coronoid process; deltpc, deltopectoral crest; fdl, deltoidian fossa; hc, humeral condyle; olef, olecranon fossa; olep, olecranon process; r, surface for radius insertion; scgl, scapula-coracoïd glenoid cavity. Scale: 1cm.

A posteroproximal fragment of the left scapula is preserved. Its medial surface is roughly planar. The deltoid fossa is wide and shallow and lies on the lateral side; it extends from the proximal extremity toward the posterior side. The glenoid cavity lies at the postero-proximal extremity, and here it forms a distinct lateral step (Fig 6A_2_). The posterior process above the glenoid cavity is short and angles almost to 90°, unlike most other ornithopods in which this posterior process is flatter and forms a sharper angle posteriorly (new character #191, see S1 Text). A very similar configuration is found in *Zalmoxes shqiperorum*, specimen UBB SO-4 [54], and *Mochlodon vorosi* [18].

#### Coracoid

A fragmentary left coracoid was recovered, with some edges partially broken. Its small size suggests that it belonged to the smallest individual. The coracoid foramen is centrally located (Fig 6B_2_). The dorsal contact for the scapula is flat, wide, and widens even more posteriorly. A deep glenoid cavity is observed posteriorly, with a high lateral edge (Fig 6B_1_).

#### Humerus

The right proximal head (MDS-VG,113) is stout, with a triangular outline in proximal view (Fig 6C_3_). The proximal articular surface is steeply inclined medially (Fig 6C_1_), as is usually observed in other ornithopods. Although the humeral head (or condyle) is partially broken, we can clearly deduce that it was on the posterior side, slightly inset from the medial border. A deeply concave fossa occurs on its posterolateral surface (Fig 6C_2_). This unusual configuration would have served for the insertion of a powerful extensor muscle. In contrast, the anterior side is completely flat (character #197 (1), see S1 Text), a morphology also found in *Mochlodon vorosi* [18]. The lateral and medial borders diverge proximally (Fig 6C_1_), the lateral side being made out as concave (even though it is incomplete proximodistally) from an anteroposterior view. The deltopectoral crest is distinct proximally, although it may rise more distally from the anterolateral border. The left distal extremity of the humerus MDS-VG,168 exhibits a typically diagonally-oriented medial condyle, whereas the lateral condyle remains straight (Fig 6D). The ulnar (medial) condyle is rounded medially, whereas the radial (lateral) condyle is characteristically flat laterally. The radial articular surface is well developed, anteriorly and posteriorly. It extends largely anteriorly (Fig 6D_1_), whereas posteriorly it reaches a point (Fig 6D_2_). The distal extremity is typically concave for articulation with the olecranon process of the ulna. The anterior coronoid fossa is very narrow, whereas the posterior olecranon fossa is wider.

#### Ulna

The proximal extremity of ulna MDS-VG,202 displays a large olecranon process, along with a sharp and anteriorly projected coronoid process (Fig 6). A radial boss is present on the proximal extremity of the lateral side (Fig 6E_1_ and 6E_2_), anteriorly to which the radius would be located. The medial side is concave anteriorly (Fig 6E_3_). The distal extremity of the ulna (Fig 6F, unnumbered fragment) consists of a rather thick plate of bone, anteroposteriorly compressed, with a very convex and rounded posterior side and a flat anterior one (Fig 6F_1_). A small, smoothly beveled distolateral surface probably articulated with the distal extremity of the radius (Figs 6F1 and 5F2). Distally, the medial side of the ulna is thinner and sharp.

### Hindlimb

#### Ilium

**Fig 7:**
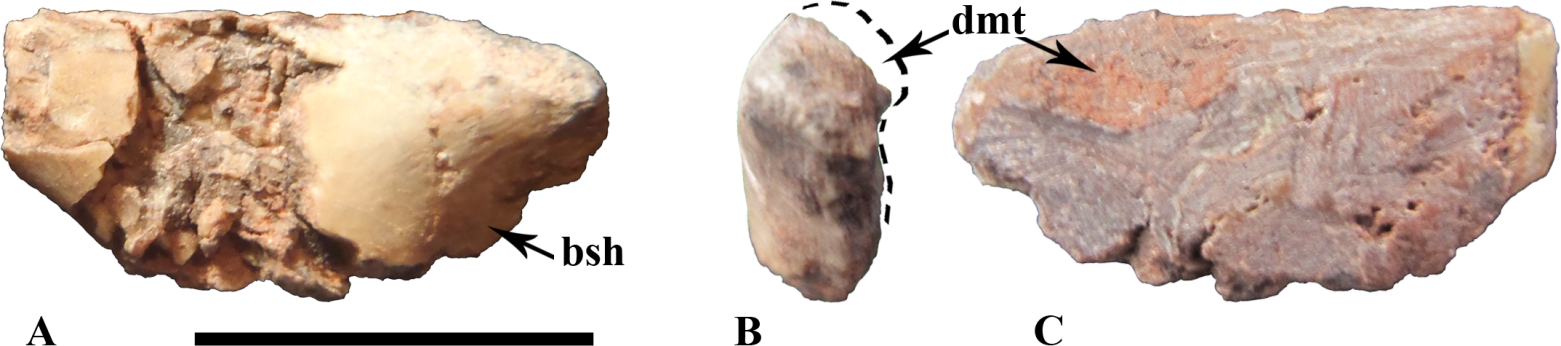
left ilium (unnumbered specimen) belonging to the smallest individual in lateral (A), posterior (B) and medial (C) views. Abbreviations: bsh, brevis shelf; dmt, dorsomedial thickening on the postacetabular process. Scale: 1cm.

Only one fragment of left ilium is preserved, belonging to the smallest individual (Fig 7). The element lacks its anterior process as well as both pubic and ischial peduncles. Its entire medial surface is damaged so that no intact bony surface remains visible in medial view. Notwithstanding, an interesting observation could be made out. A bony outgrowth appears bulging postero-laterally, indicating that there was probably some medial thickening of the posterodoral margin (Fig 7B and 7C). But, as no bony surface remains intact medially, the exact morphology of this outgrowth cannot be determined. The principal features of the ilium are visible from the lateral view. These are apparently plesiomorphic for ornithischians: 1) the dorsal margin of the ilium is almost straight to slightly convex; and 2) the brevis shelf doesn’t form a distinct “step” but it faces completely ventrolaterally, so that it seems completely absent at first sight (Fig 7A and 7B).

#### Femur

**Fig 8:**
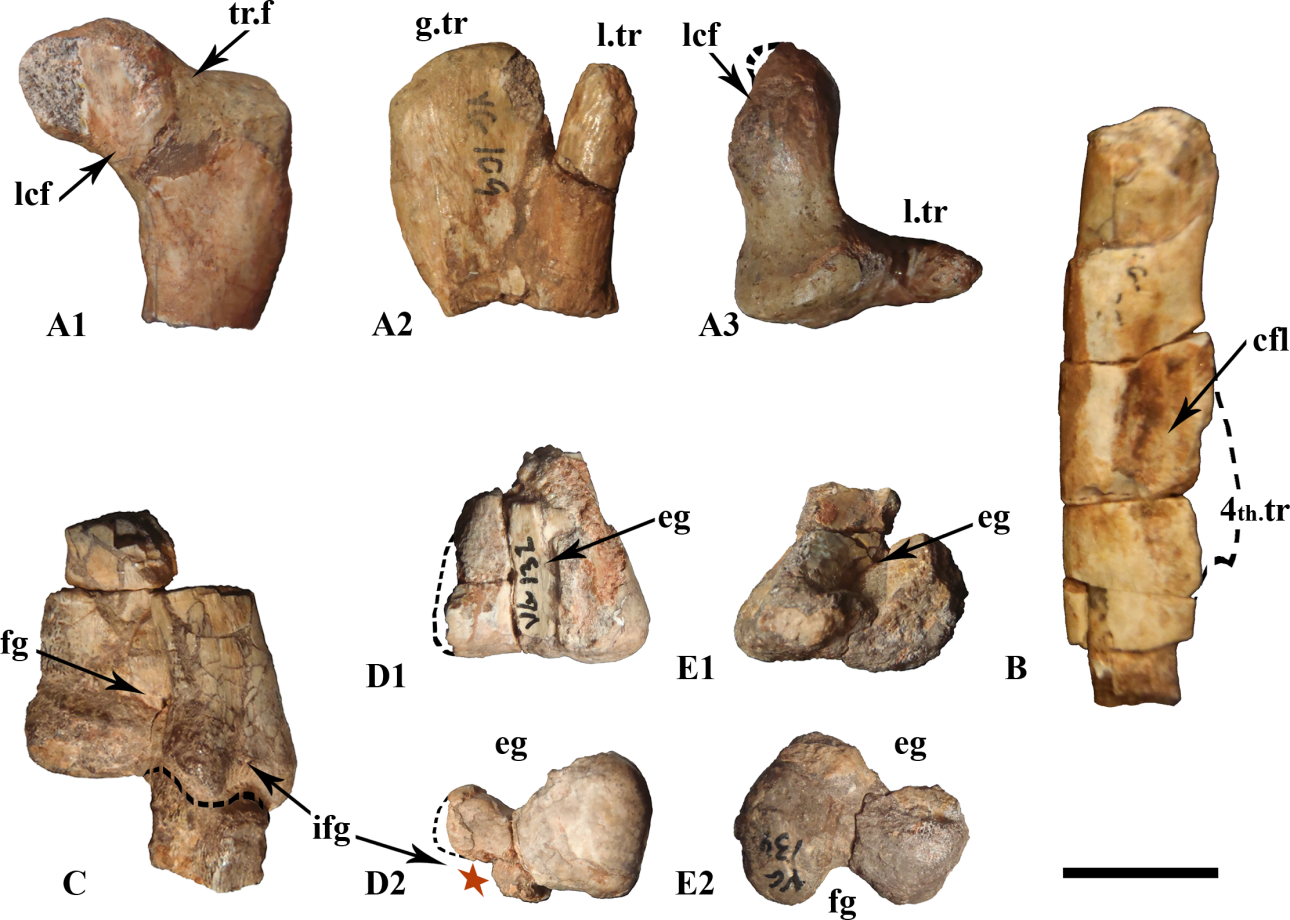
Femora. The proximal extremity belonging to the largest sized individual, MDS-VG,109, is figured in A_1_ posterior, A_2_ lateral, and A_3_ proximal views. The diaphysis fragment (B) belongs to the medium-sized individual and is in medial view. The distal fragments MDS-VG,135 (C), MDS-VG,132 (D), and MDS-VG,134 (E) belong respectively to the largest and two medium-sized individual. They are in posterior (C), anterior (D_1_/E_1_) and distal (D_2_/E_2_) views. Abbreviations: 4th.tr, fourth trochanter; cfl, *caudifemoralis* muscle scar; eg, extensor groove; fg flexor groove; g.tr, greater trochanter; ifg, *ilio-fibularis* muscle groove; l.tr, lesser trochanter; lcf, sulcus for the *ligamentum capitis femoris*; tr.f, *trochanteris fossa*. Scale: 1 cm.

Proximally, the inner articular head is well elevated upward, forming an average angle of 22° with respect to the horizontal plane (measured on MDS-VG,108 and MDS-VG,109, Fig 8A_1_ and 8B). The *fossa trochanteris* is shallow (Fig 8A_1_). The posterior surface of the inner articular head is concave to receive the *capitis femoris* ligament (Fig 8A_3_). The articular surface of this head is well expanded and plunges anteriorly. The greater trochanter is flat to slightly concave laterally. The posterolateral edge is rounded for the *M*. *ilio-trochantericus* insertion. The smooth concavity located more anteriorly serves for the *M*. *pubo-ischiofemoralis internus I* insertion [28]. The lesser trochanter is a digit-like process, rounded in lateral view and flat in medial view, inserted anterolaterally into the proximal part of the greater trochanter. In MDS-VG,109 (Fig 8A), the lesser trochanter is characteristically expanded anteroposteriorly and narrow mediolaterally. It does not quite reach the same height as that of the greater trochanter (Fig 8A_2_). No fourth trochanter was preserved, it was found broken off from its medial insertion on the femur diaphysis MDS-VG, 122. Nevertheless, we can deduce from the same fragment that its insertion was proximodistally expanded (Fig 8B). Medially, the *M*. *caudifemoralis longus* scar is elongated proximodistally and quite anteriorly located. This muscle scar merges roughly with the broken fourth trochanter, though a smooth separation can be seen punctually at mid-length proximodistally, between this scar and the fourth trochanter insertion (Fig 8B). All of the distal extremities of the femora display an anterior intercondylar groove for the extensor *M*. *ilio-tibialis* (Fig 8D_2_ and 8E_2_), except MDS-VG,135, which is highly damaged in this zone. On the posterior side, a deep flexor groove is visible, which is not overlapped by the medial condyle. The medial condyle is typically flat in its inner medial surface, whereas the lateral one is more rounded externally. All of the distal parts of the femora are much wider mediolaterally than tall dorsoventrally (Fig 8D and 8E). The largest femur, MDS-VG,135, clearly displays a medially deviating posterolateral condyle (Fig 8C). This opens a posterolateral notch for the *M*. *ilio-fibularis* passage. The same notch is observed in MDS-VG,132, although its posterolateral condyle is inwardly crushed (Fig 8D_2_). In the smaller MDS-VG,134, the lateral deflection of this posterolateral condyle is absent, and an equivalent inclined surface is observed. In distal view the medial condyle protrudes largely beyond the lateral condyle anteriorly (character #261, S1 Text and Fig 8E_2_).

#### Tibia

**Fig 9:**
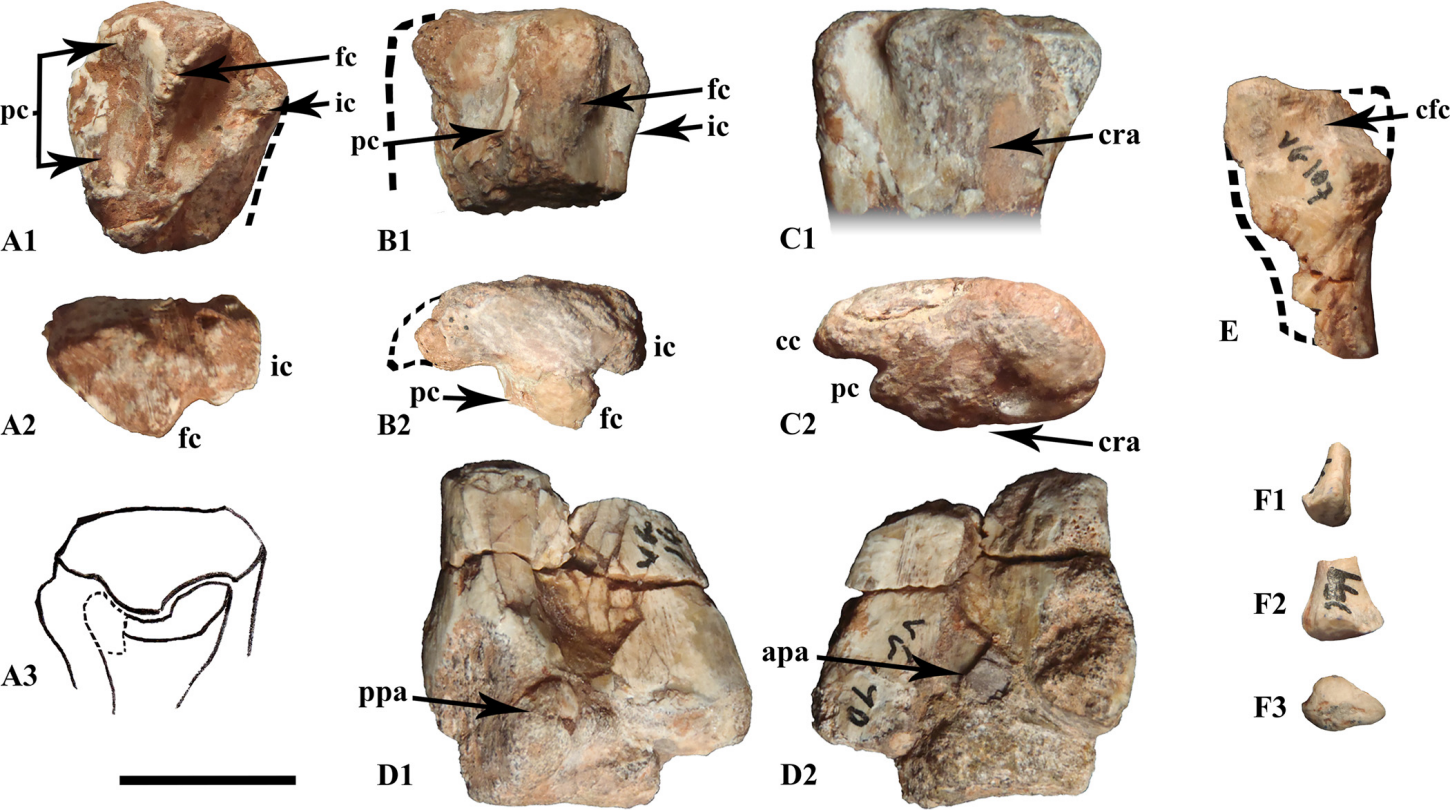
Tibiae and fibulae. The proximal articular extremities of tibiae MDS-VG,137 (A), unnumbered tibia (B) and MDS-VG,136 (C) in lateral (A_1_, B_1_, C_1_) and proximal (A_2_, B_2_, C_2_) views. MDS-VG,107 fits with MDS-VG,137, as shown in the drawing (A_3_) in proximolateral view. The distal extremity of tibia MDS-VG,140 is represented in posterior (D_1_) and anterior (D_2_) view. The proximal extremity of fibula MDS-VG,107 is represented in medial view (E). The distal extremity of fibula MDS-VG,199 is represented in lateral (F_1_), anterior (F_2_) and distal view (F_3_). Abbreviations: apa, anterior ascending process of the astragalus; cfc, cavity for the fibular condyle; fc, fibular condyle; cc, cnemial crest; cra, crushed area around the fibular condyle; ic, inner condyle; pc, precnemial crest; ppa, posterior process of the astragalus. Scale: 1 cm.

The proximal fragments of the tibiae are anteroposteriorly expanded. The medial surface is straight and flattened. The cnemial crest is robust proximally and sharper distally (unfigured proximal tibia MDS-VG,136). On the posterior side, the inner condyle is stout, but no lateral condyle is identified. This latter condyle could have disappeared by itself or by fusion with the more anterior accessory condyle [55]. We named this condyle the “fibular condyle”, as it would have articulated with the proximal articular head of the fibula. This fibular condyle thus rises at mid-length anteroposteriorly. In proximal view it forms a large plateau, but shrinks immediately more distally to articulate with the proximal extremity of the fibula (Fig 9A_3_). Introduced as a term by Parks for the tibia of *Parksosaurus warreni* [56], the precnemial crest forms another ridge anteriorly located with respect to the fibular condyle (Fig 9B_2_, unnumbered) and forms an anterior buttress for the head of the fibula (Fig 9A_1_ and 9B_1_). In MDS-VG,137 (Fig 9A_2_) the precnemial crest is not prominent, so the more anterior *incisura tibialis* cannot be observed in proximal view. The distinctly pronounced *incisura tibialis* is noteworthy in two larger ontogenetic stage 2 specimens (Fig 9B_2_ and 9C_2_), in which the fibular condyle and the precnemial crest are both stouter and more robustly developed. The tibial diaphysis is long, thin and tubular, and expands lateromedially toward its distal extremity. It bears an anterior longitudinal groove, which is accentuated distally just before it reaches the two distal malleoli. The medial malleolus is thicker than the lateral one. The anterior ascending process of the astragalus is shown here in the largest specimen (MDS-VG,140, Fig 9D_2_) as a large blade of bone attached to the anterodistal side of the tibia, which ends as a spike dorsally. The narrow lateral malleolus has a flat anterior surface for the contact with the distal end of the fibula.

#### Fibula

The fibula MDS-VG,107 is a left proximal fragment. It is bifid and anteroposteriorly expanded. A triangular cavity is located proximomedially to receive the fibular condyle of the tibia (Fig 9E). Indeed, MDS-VG,107 fits perfectly with the left proximal part of tibia MDS-VG,137 (Fig 9A_3_). The left distal extremity of fibula MDS-VG,199 (Fig 9E) would have belonged to the smallest (ontogenetic stage 1) individual. Its posterior surface is flat and would have been superposed over the lateral condyle of the tibia. Its distal articular surface forms a sort of lip which rises facing slightly anteriorly (Fig 9F_1_). Overall, the distal extremity of the fibula is thicker anteroposteriorly on the medial side than on the lateral side (Fig 9F_2_ and 9F_3_).

#### First metatarsal

**Fig 10:**
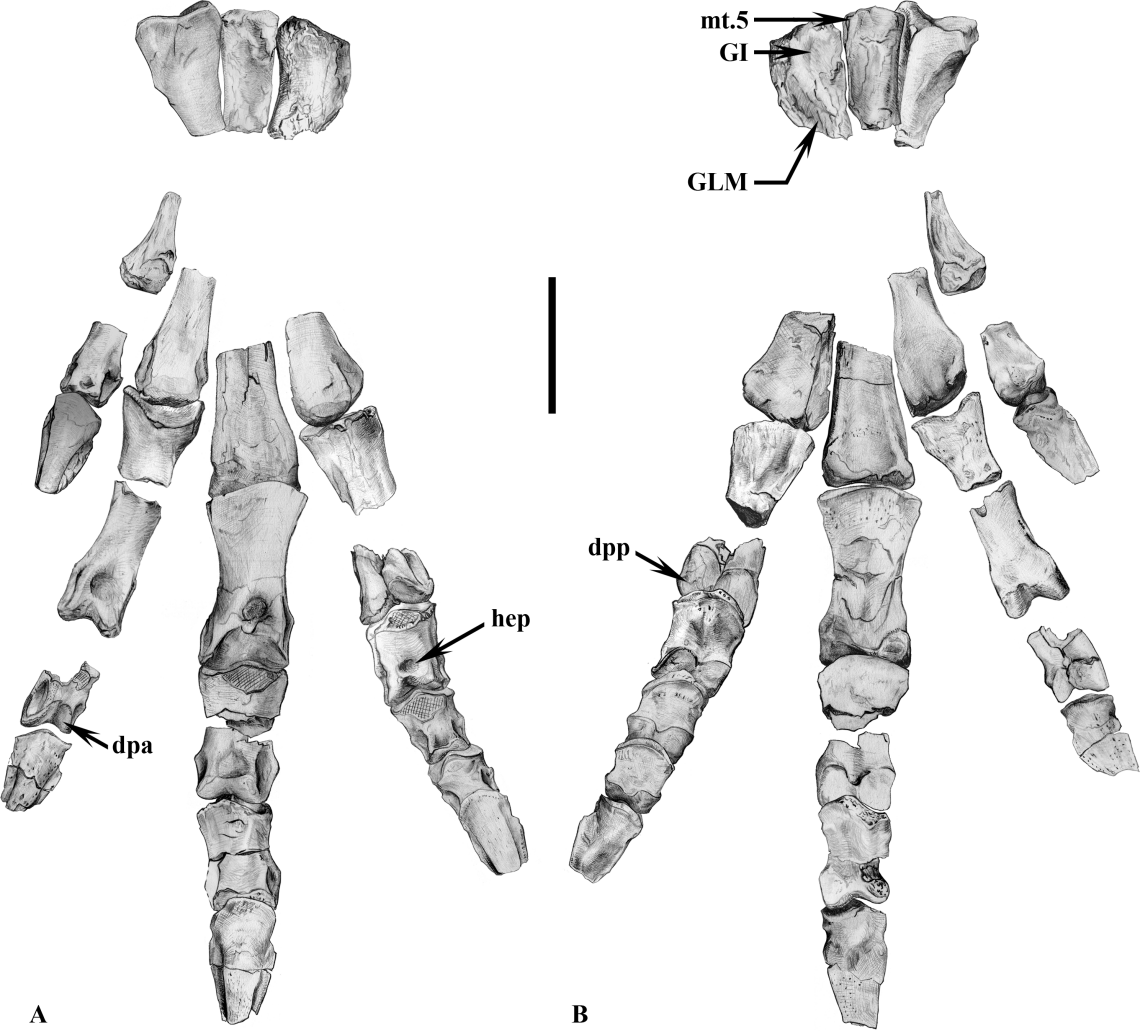
Reconstruction of the Vegagete left foot in anterior (10A) and posterior (10B) views. MDS-VG,171 (reversed) represents the distal extremity of the first metatarsal, MDS-VG,177 and 160, 174 and 163, 178 and 169 represent respectively the proximal and distal extremities of the second, third, and fourth metatarsals. Pedal phalanges are noted « X-n » for referring to the “n^th^” phalanx of digit X: distal I-1 (MDS-VG,239), claw I (MDS-VG,266), proximal II-1 (MDS-VG,232), distal II-1 (MDS-VG,210 reversed), distal II-2 (MDS-VG,233), claw II (MDS-VG,276), entire phalanx III-1 (MDS-VG,209), proximal III-2 (MDS-VG,237 reversed), distal III-2 (MDS-VG,254), entire phalanx III-3 (MDS-VG,245), claw III (MDS-VG,272), proximal IV-1 (MDS-VG,211 reversed), distal IV-1 (MDS-VG,215 reversed), and entire phalanges IV-2,3,4 (respectively MDS-VG,243, 240, 247), claw IV (MDS-VG,258 reversed). Specimens MDS-VG,210, 237, 211, 215, and -258 belong to a right foot. Abbreviations: dpa, distal pulleys anterior articular surface; dpp, distal pulleys posterior articular surface; mt.5, fifth metatarsal; GI: insertion zone for M. *gastrocnemius internus*; GLM: insertion zone for M. *gastrocnemius pars lateralis et medialis*; hep, hyper-extensional pit. All bones except metatarsal I, claw I and claw IV belong to the medium-sized individual; for them the scale represents 1 cm. Metatarsal I, claw I and claw IV belong to the larger individual; for them the scale represents 1.25 cm.

The very thin and fragile structure of the broken proximal portion of the first metatarsal (MDS-VG,171) suggests a nearly absent articulation with the astragalus. A lack of an articular surface for the astragalus has already been observed in *Gideonmantellia* [31], *Othnielosaurus* [57] and *Parksosaurus* [56]. The cross-section of the proximal first metatarsal is in the form of a thin isosceles triangle. This feature has already been observed in the articulated pes of *Muttaburrasaurus* [50], in which case the small base was told to be anteriorly located. Basing on this character, we deduce that MDS-VG,171 was a right distal fragment. The distal condyle strongly bulges distally (MDS-VG,171). In most basal neornithischians, the distal condyle of metatarsal I bulges in anterior direction with ligamentary fossae oriented medio-laterally [28, 57]. However in the Vegagete ornithopod the presumed plantar surface is unusually flat. We suggest that the flat posterior side actually faced laterally toward the medial side of the second metatarsal and that the anterior bulge was directed medially. This has been found to occur in the Gondwanan ornithopods *Anabisetia* [53] and specimen VOPC III [58]. Let’s note that Herne [58] describes for VOPC III an orthogonal torsion of the whole first digit starting from the proximal articulation of phalanx I_1_, which makes the first pedal digit being redirected planto-posteriorly as occurs for the other pedal digits. This configuration used as a hypothesis for the reconstruction of the Vegagete ornithopod foot (Fig 10).

#### Second metatarsal

The proximal part of the second metatarsal (MDS-VG,177) would have been elongated anteroposteriorly, being wider dorsally and narrowing drastically in its ventral part (Fig 10A). The thinner ventral surface was unfortunately broken off. The proximal articular surface is strongly concave to articulate with the medial tarsal. The lateral side is concave so that it could smoothly enclose the third metatarsal. By contrast, the medial side is more planar overall, probably to accommodate the adjacent first metatarsal. The shaft narrows drastically distally (MDS-VG,160). The distal articular head is very close in shape to that of *Hypsilophodon foxii* [28]. It is convex, very prominent, and displays a trapezoid outline.

#### Third metatarsal

The proximal extremity of the third metatarsal (MDS-VG,174) is expanded anteroposteriorly and compressed mediolaterally. The posterolateral side of the third metatarsal together with the posteromedial side of the fourth metatarsal bears a cavity that probably served as an articular facet for the fifth metatarsal. Ventrally, a smooth longitudinal cavity may have hosted the *M*. *gastrocnemius internus* [59–61], (Fig 10B). Insertions for the *M*. *gastrocnemius pars lateralis et medialis* are poorly developed. In the distal portion (MDS-VG,163), the shaft becomes smaller in height and expands notably mediolaterally. Distally, the shaft is marked by a small mediodistal bump (Fig 10A). Butler *et al*. [62] explain this feature as related to the ability of digit III to support hyperextension.

#### Fourth metatarsal

Proximally, the fourth metatarsal (MDS-VG,178) is sub-triangular in outline. The ventral part is shallowly concave to host the *M*. *gastrocnemius internus* insertion. The shaft thins rapidly and twists medially. The ventrolateral part becomes widely concave only a short distance from the proximal extremity, and hosted the insertions of the *M*. *gastrocnemius pars lateralis et medialis* [59, 61], (Fig 10B). The distal-most extremity of the fourth metatarsal (MDS-VG,169) bulges anteriorly. A posterolateral crest floors the well-developed lateral ligamentary fossa for the insertions of the *M*. *gastrocnemius pars lateralis et medialis*.

#### Fifth metatarsal

The fifth metatarsal is identified here as a very small bone attached to the very proximolateral side of the third metatarsal (MDS-VG,174). It is broader and plate-like in the first third of its length, after which it thins abruptly until its distal end (Fig 10B).

#### Pedal proximal phalanges

**Fig 11:**
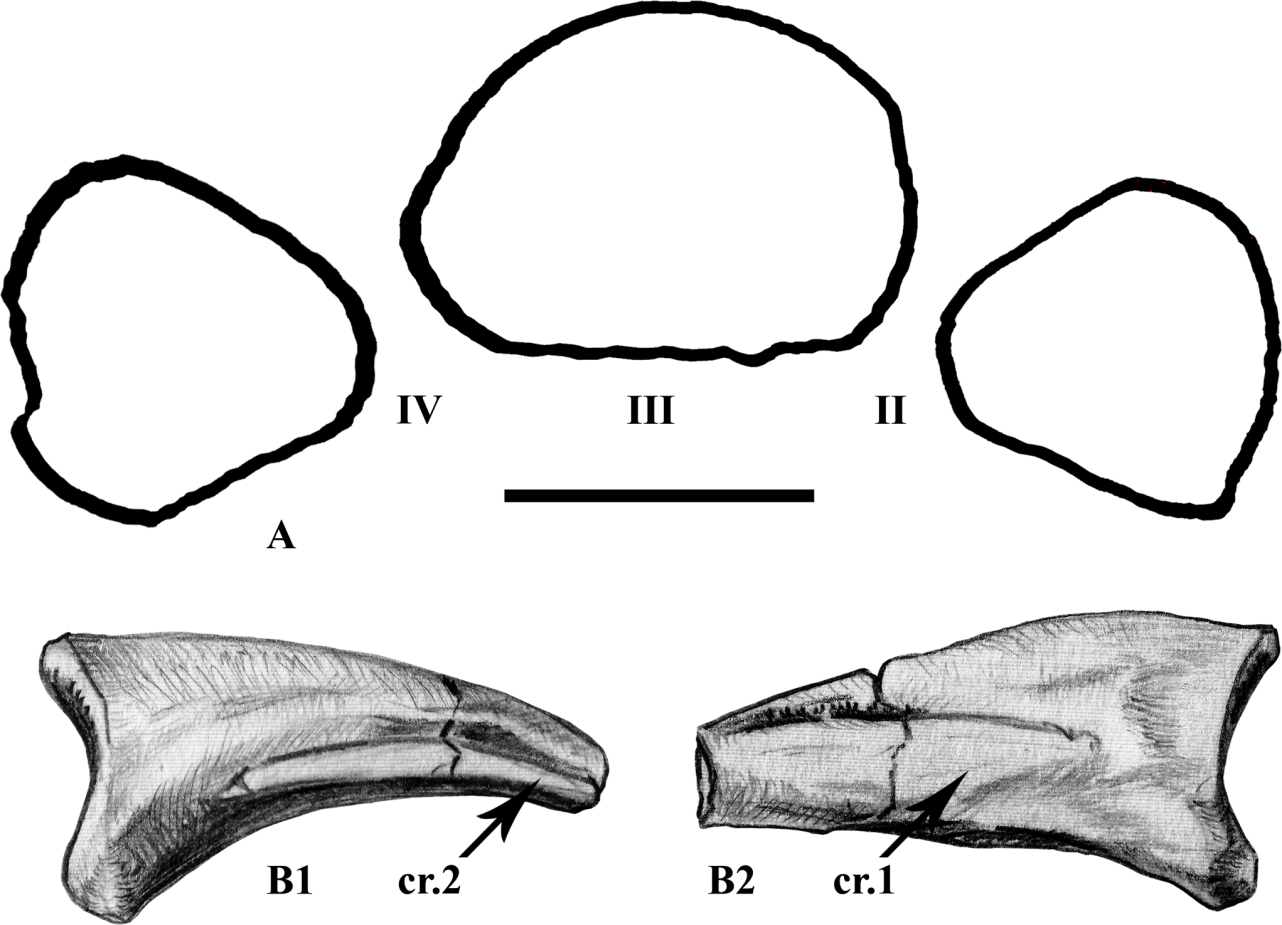
Some features of the pedal phalanges. Proximal outline of the first phalangeal row of digit IV (MDS-VG,211 reversed to left), III (MDS-VG,209), and II (MDS-VG,232) in a hypothesized left pes (A) and right claw-like phalange from digit III (MDS-VG,262) in lateral (B1) and medial (B2) views. Abbreviations: cr.1 and 2, types 1 and 2 ventrolateral ridges. Scale: 5mm.

A hypothetical left pes belonging to a medium-sized individual is reconstructed based on isolated pedal phalanges and metatarsals. Phalangeal positions are based on proposals of Thulborn [55], Galton [28], and Ruiz-Omeñaca [63]. The phalangeal formula should be (2-3-4-5-0), a plesiomorphic condition for many ornithopods. The extensor ligament insertions, or “hyperextensional pits” [61], are dorsally situated between the two distal articular pulleys. In the Vegagete ornithopod, these pits have the form of small diamonds that are found on the first phalangeal row of every digits (I_1_, II_1_, III_1_, IV_1_) and which persist until the second phalangeal row of digit II and the third phalangeal row of digits III and IV (Fig 10A). The overall length of the phalanges diminishes distally. The phalanges are attributed to their respective digit on the basis of their proportions [63]: the thinnest belong to digit I; the longest phalanges are attributed to the digit II; the widest belong to digit III; and the shortest and most robust ones are attributed to the digit IV (Fig 10). To identify the proximodistal location of the phalanges, we differentiate among proximal, intermediate and distal (or ungual) phalanges. The proximal phalanges bear a unique proximal articular facet. From the second row until the last distal phalanges, a thin median sinus separates the proximal cavity into two articular facets [28]. The extensor ligament insertion of the phalanges is sometimes observed in the proximal and intermediate phalanges, in the form of small diamond-shaped pits, dorsally situated between the two distal articular pulleys. In order to correctly reconstruct the foot and to distinguish phalanges from a right or from a left foot, we looked for some asymmetrical mediolateral features of the phalanges (see Fig 11A). The lateral edge of digit II phalanges is the highest, straightest and most vertical edge. The opposite is the case for the digit III phalanges, in which this applies to the medial edge. This criterion is less applicable to digit IV. Instead, digit IV phalanges display a conspicuous lateral angular process, in the form of a well-marked, sudden horizontal shelf on their lateral edge. This proximolateral angular shelf can in some instances become very faint to non-existent in more distal phalanges. Digit III phalanges display a very discrete obtuse ventrolateral angle [5]. The distal pulleys could also be diagnostic to distinguish phalanges from a left or from a right foot. In digit II, the medial pulley is characteristically more expanded anteroposteriorly. Moreover, it is also more expanded proximodistally in the second phalanx (II_2_), (Fig 10A). The distal pulleys of the digit III phalanges are symmetrical, with no distinguishable criterion of asymmetry. In digit IV, the medial pulley is wide mediolaterally, and displays a flat, anteriorly-facing articular surface. This feature was also observed by Ruiz-Omeñaca [31] in *Gideonmantellia*. *amosanjuanae*. Phalanx I_1_ could only be oriented following Ruiz-Omeñaca [31]. It appears that in *G*. *amosanjuanae*, the hallux bears a more angled distolateral pulley.

#### Pedal unguals

Four claw morphotypes have been found. Each of them may correspond to one of the four pedal digits. Claws from digits II, III, and IV (respectively MDS-VG,262, 272, 258) have been determined thanks to their proximal outline, which is related with that of their adjoining proximal phalanges. The last claw morphotype from the assemblage was deduced to belong to the first digit (MDS-VG,266). As in *Anabisetia* [53] and *Lesothosaurus* [55], it is distinguished by its dorsoventrally flattened nature and its very flat ventral surface. Ventro-proximally, a slight, centered concavity served as the attachment area for the *M*. *flexor hallucis longus* [64]. It is noteworthy that part of the digit II claw sample displays a similar muscle attachment site, but this time for the insertion of the *M*. *flexor digitorum longus* [64]. The *M*. *flexor digitorum longus* attachment area is fainter in the claws of the other digits. In the Vegagete specimen, the digit I claws present a proximomedially placed ligamentary pit, probably for the insertion of the *M*. *extensor hallucis longus* [61]. This pit is faint to completely absent in other digits.

All of the unguals possess two ventral ridges that are clearly distinguishable from one another. These rise more or less for the first third of the total claw length. The first type of ridge is narrow and remains close to the body of the claw. It is thicker and more rounded ventrally. A furrow excavates well above the ridge into the body of the claw (Fig 11B2). The second type of ridge is wider lateromedially, and angles out strikingly from the body of the claw proximally. It is thin and sharp. It forms the floor of the body of the whole claw in digits II, III and IV, but not in the first digit, in which it rises a bit higher. The furrow is smoother above it. Whichever the claw in question, the first type of ridge should be found medially and the second type of ridge should be found laterally (Fig 11B1). However, detailed information on articulated feet from other ornithopods is still unavailable to confirm this.

## Discussion

### Ontogenetic considerations

#### Vertebral column

**Fig 12:**
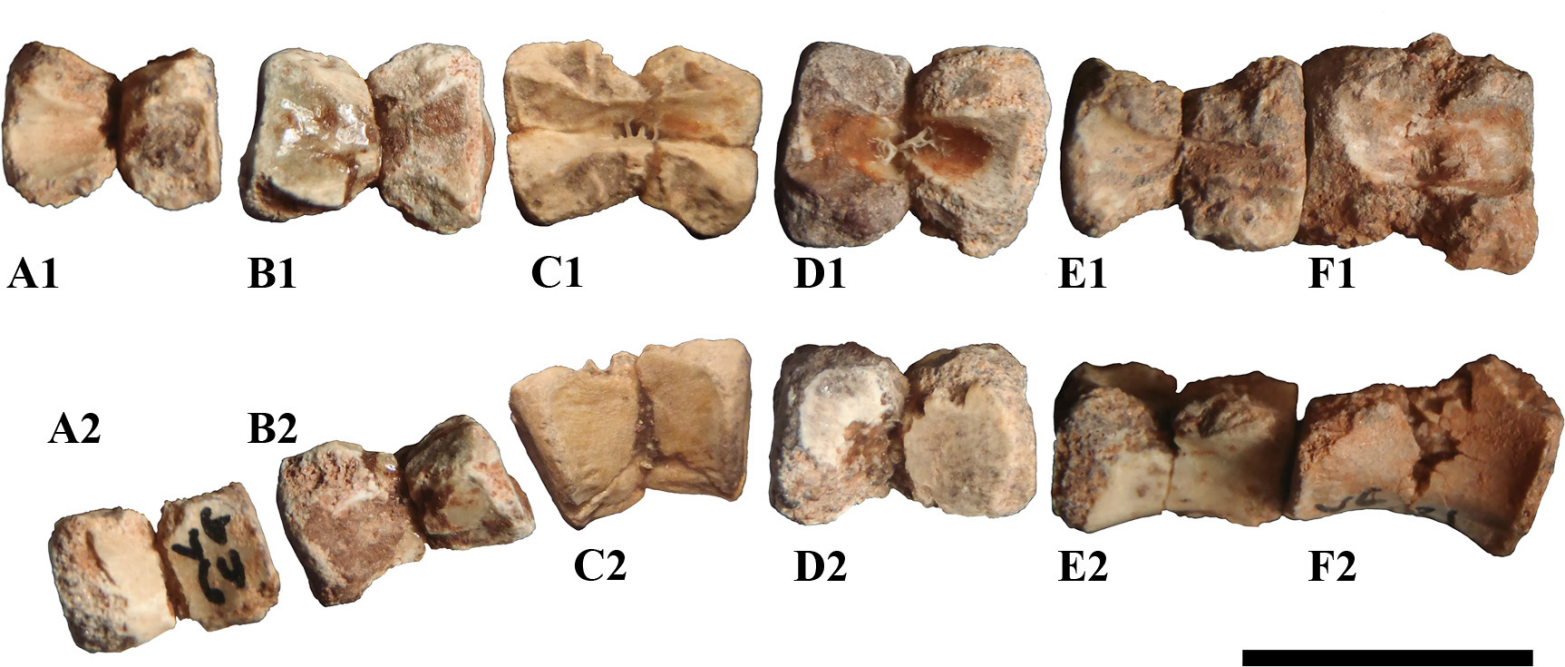
Partial reconstruction of the tiniest individual’s back from the anterior dorsal vertebrae (left) to the anterior part of the sacrum (right). Views are dorsal (A_1_-F_1_) and lateral (A_2_-F_2_). Dorsal vertebrae are from (A) to (D), the dorsosacral is (E) and articulates with the first true sacral which is (F). Their identifying numbers are respectively MDS-VG,64 (A), MDS-VG,75 (B), MDS-VG,57 (C), MDS-VG,67 (D), MDS-VG,79 (E), MDS-VG,81 (F). Scale: 1cm.

Specimen MDS-VG,57 is a centrum that belongs to a mid-series dorsal vertebra from the smallest individual of the sample, i.e. a juvenile individual (first ontogenetic stage, see Fig 12C). It is homologous in position to the ontogenetic stage 3 dorsal centrum MDS-VG,66 (Fig 5E). Apart from their typical mid-dorsal series characteristics, the two specimens differ from each other in an important feature: the smaller centrum MDS-VG,57 has anteroposterior articular surfaces that are well inclined from the vertical (up to 20°) and thus convergently angled ventrally. Among the few juvenile dorsal vertebrae found, MDS-VG,57 is the only one that bears such characteristics. There is one vertical fracture observed in the middle of the centrum, but is unlikely to be the result of compression, because the centrum kept a very regular profile and such fractures were also observed in many other centra, seemingly undeformed as well. MDS-VG,57 would have kept its natural proportions. In contrast, the articular facets from the ontogenetic stage 3 centrum (MDS-VG,66) lacks any kind of ventral inclination. MDS-VG,57 is very likely to have acted as a keystone for the downward arching of the juvenile’s back. This bending would disappear later during more advanced ontogenetic stages. It should be remembered that this phenomenon in the dorsal vertebrae has already been reported in each of the rhabdodontid genera. Anteroposterior articular facets in *Mochlodon* are inclined up to 5° from the vertical [18]. In *Zalmoxes*¸ both *Z*. *shqiperorum* and *Z*. *robustus* display this inclination, but no measurement is given [22]. With regard to *Rhabdodon*, Chanthasit [65] states that the posterior dorsal vertebrae display this ventral convergence, but no measurement is given. To conclude, the Vegagete ornithopod shares with rhabdodontids the ventrally converging articular facets of the dorsal centra, but only at the most juvenile stage. More importantly, this feature points toward a change of posture from a downwardly arching dorsal column in the juvenile toward a straighter dorsal column in the adult. Although we could not assess whether the juvenile individual from Vegagete was quadrupedal or not, its body would have been more strongly arched downwardly than bigger individuals from the same species. A transition from a juvenile quadrupedal stance to an adult bipedal stance has already been demonstrated for *Dysalotosaurus lettowvorbecki* [66]. In the Vegagete ornithopod, the structure of any of the ontogenetic stage 2 feet bones is slender, which suggests a digitigrade posture typical of bipedal ornithopods [67].

#### Femur

**Fig 13:**
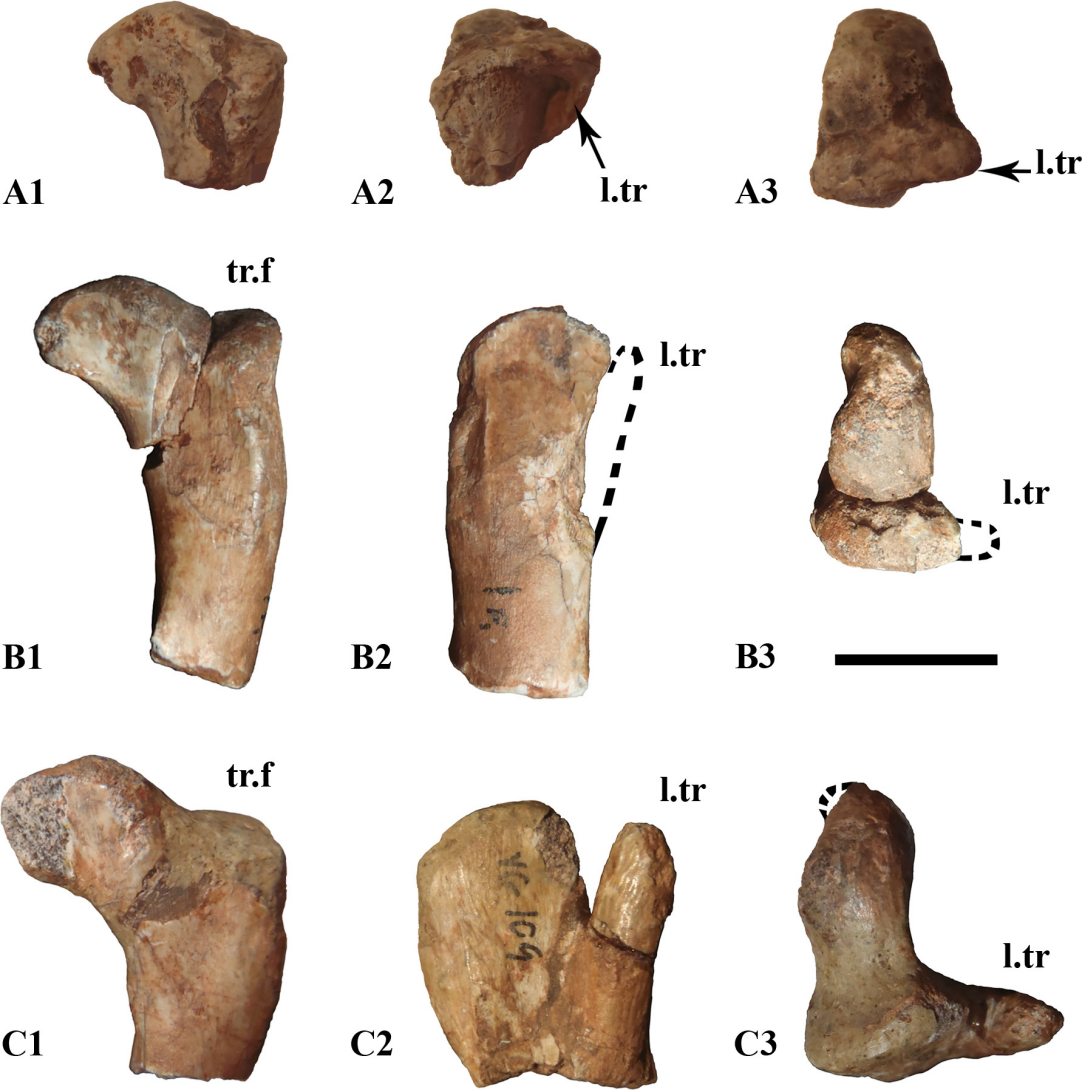
Evolution of the proximal extremity of femur’s shape throughout ontogeny. The three proximal extremities are numbered MDS-VG,159 (A), MDS-VG,108 (B) and MDS-VG,109 (C), representing ontogenetic stages 1, 2 and 3 respectively. Views are posterior (A_1_, B_1_, C_1_), lateral (A_2_, B_2_, C_2_), proximal (A_3_, B_3_, C_3_). Abbreviations: l.tr, lesser trochanter; tr.f, *trochanteris fossa*;. Scale: 1cm.

MDS-VG,109 should belong to the largest individual (ontogenetic stage 3), MDS-VG,108 to one medium-sized individual (ontogenetic stage 2), and MDS-VG,159 to the smallest individual (ontogenetic stage 1). Of note is the strikingly anteroposteriorly expanded lesser trochanter in MDS-VG,109. Interestingly, the anterior (or lesser) trochanter of MDS-VG,108, though broken and lost, would not have been so expanded anteroposteriorly as in MDS-VG,109 (Fig 13B_2_). This is deducible because otherwise the lesser trochanter would have had to bulge anteriorly in its base. MDS-VG,159 stands out by its small proportions (e.g. the shortness of its articular head), and by the fact that the lesser trochanter is stuck to the greater trochanter medially and by a proximal cap of bone. A trough is still observable laterally between both the greater and the lesser trochanters. Two arguments are in favor of the smallest individual belonging to the same taxa. Firstly, the evolution of the proximal femoral extremities’ shapes passing from MDS-VG,159 through MDS-VG,108 and to MDS-VG,109 is gradual and thus consistent with a continuous growth series (Fig 13). The *fossa trochanteris* may deepen gradually with growth, as the femoral head becomes more prominent and angles more upward medially. Congruently, the lesser trochanter is very likely to split itself progressively in the anterior direction with age. In MDS-VG,159, the proximal extremity is globular. The proximal articular head and greater trochanter become both slenderer in aspect in MDS-VG,108 and even more so in MDS-VG,109 (Fig 13A_3_–13C_3_). Identical observations have been made on femora from *Leaellynasaura amicagraphica* [68–69], where the numerous juvenile femora have very little separation between the femoral head and the greater trochanter, and there appears to have been no separation between lesser and greater trochanter. These features develop in the more adult individuals: (NMV 179564) and Victorian ornithopod femur type 1. A small femur specimen, MHNAIX-PV.2015.13.1, from the Late Cretaceous of Aix-En-Provence is strikingly similar to MDS-VG,159. Its general aspect is more globular and its femoral head and greater trochanter are very shortly expanded. We argue that these femoral charateristics are most probably typical of juvenile individuals.

Studies on ontogenetic variation in the femora of the theropod *Allosaurus* [70] and the archosaur *Silesaurus opolensis* [71] pointed out that the anteroposterior length of the greater trochanter grows with age more than the proper mediolateral width of the articular head. In the Vegagete ornithopod, however, a close look at the proximal femoral extremities (Fig 13) shows the opposite. The articular head of MDS-VG,109 is strikingly wider mediolaterally than that of MDS-VG,108, and the greater trochanter is only a little longer than that of MDS-VG,108.

In the Vegagete ornithopod, the distal femoral fragment MDS-VG,134 bears an *ilio-fibularis* notch that is less developed than seen MDS-VG,132 and MDS-VG,135. It has been previously argued that a more developed posterolateral “notch” for this muscle is associated with a more adult stage in the ontogeny of *Hypsilophodon foxii* [34]. It is probably the same for the Vegagete ornithopod. However, MDS-VG,132 and MDS-VG,134 are both medium-sized (ontogenetic stage 2). Because the specimens all apparently belong to the same species, one might hypothesizes that the sexual identity of MDS-VG,132 and MDS-VG,134 was different, and that this would have led to the appearance of a differentially stronger or weaker *ilio-fibularis* notch during the growth of the two individuals. Another possibility is mere inter-individual variability.

#### Tibia

The proximal extremities of three tibiae are present in the material (MDS-VG,136, MDS-VG,137 and another unnumbered proximal extremity). Only the first two are completely preserved anteroposteriorly. MDS-VG,137 apparently belongs to an early ontogenetic stage 2, whereas MDS-VG,136 and the unnumbered fragment would belong to a slightly more advanced ontogenetic stage 2. MDS-VG,136 is somewhat crushed mediolaterally onto its shaft and more proximally onto its fibular condyle (as shown in Fig 9C). However, because it is not broken, MDS-VG,136 allows us to discuss two noticeable morphological features that may be linked to ontogeny. In MDS-VG,137 the origin of the cnemial crest is very narrow, beginning distally with respect to the proximal extremity and thus forming a slight step (see Fig 9A_1_). However, in MDS-VG,136, the cnemial crest protrudes immediately from the proximal articular surface (Fig 9C_1_). In MDS-VG,137, the *incisura tibialis* is almost non-existent in front of the precnemial crest and the cnemial crest is very short anteroposteriorly (Fig 9A_2_). In the two bigger specimens (MDS-VG,136 and the other unnumbered fragment), the fibular condyle and the precnemial crest stand out so that the *incisura tibialis* becomes very clearly observable in front of the precnemial crest; as well the cnemial crest appears to be more expanded anteriorly (Fig 9B_2_ and 9C_2_).

### Phylogenetic analysis

The Vegagete taxon was included in a maximum parsimony analysis combining four recent phylogenetic studies [8, 18, 72, 73] with modifications and additions of characters and characters states. The combination of these matrices allows a better discrimination of the monophyletic group Rhabdodontidae within a global ornithischian phylogeny [18], and also enhances the resolution of basal iguanodontians [73] and that of basal ornithopods [72]. We add five new characters (#191, #197, #277, #278, #279). The character list and all modifications are available in the S1 Text.

The phylogenetic analysis was run under equally-weighted maximum parsimony using TNT (Tree Analysis using New Technology) [74]. A heuristic search of 1000 replications of Wagner trees (with random addition sequence) was performed, followed by a Tree Bisection Reconnection branchswapping algorithm (holding 10 trees per replicate and using the collapsing rule 3 for zero-length branches). Zero-length branches among any of the recovered most parsimonious trees (MPTs) were collapsed. Characters were treated as unordered except for: #126, #159, #167, #168, #170, #222, #223, #225, and #262, the same characters that were ordered in Ösi *et al*. (2012). Bremer and bootstrap indices were obtained using TNT. *Herrerasaurus ischigualastensis* was used as the outgroup taxon.

Though discussed in the text, *Gideonmantellia* was not included a posteriori in the phylogenetic analysis because of its incompleteness. For additional resolution, the following OTUs were not used in this analysis: *Euparkeria*, *Marasuchus*, *Silesaurus*, *Asilisaurus*, *Sanjuansaurus*, *Tawa*, NHMUK RUA 100, Stegosauria, Ankylosauria, *Micropachycephalosaurus*, *Stenopelix*, *Wannanosaurus*, *Goyocephale*, *Homalocephale*, Pachycephalosauridae, *Chaoyangsaurus*, *Liaoceratops*, *Archaeoceratops*, *Albalophosaurus*, *Leaellynasaura*, *T*. *assiniboiensis*, *T*. *garbanii*, *Notohypsilophodon*, *Oryctodromeus*, Kaiparowits Orodromiines, *Atlascopcosaurus*, *Qantassaurus*, *Elrhazosaurus*, *Valdosaurus*, *Ouranosaurus*. *Stormbergia dangershoeki* was previously recognized as undiagnostic [75] and was therefore omitted from this analysis too. Dryosauridae was split into *Dryosaurus altus* and *Dysalotosaurus lettowvorbecki*. We completely recoded *Rhabdodon priscus* Matheron [20], based on a revision of historical material (Tortosa, in progress). Actually, *Rhabdodon* character scores were originally amalgamated with many undiagnosed *Rhabdodon*-relative specimens from the same locality. Recent discoveries suggest the presence of distinctly separate species from this assemblage [18], which is the reason why we rescored *Rhabdodon* sp. 1, a partial individual from Vitrolles, originally described by Pincemaille-Quilleveré [21] and now under review. We also added the basal iguanodontoid *Muttaburrasaurus langdoni* [50]. The taxon-specific bibliography that was used for coding is provided in S2 Table.

**Fig 14:**
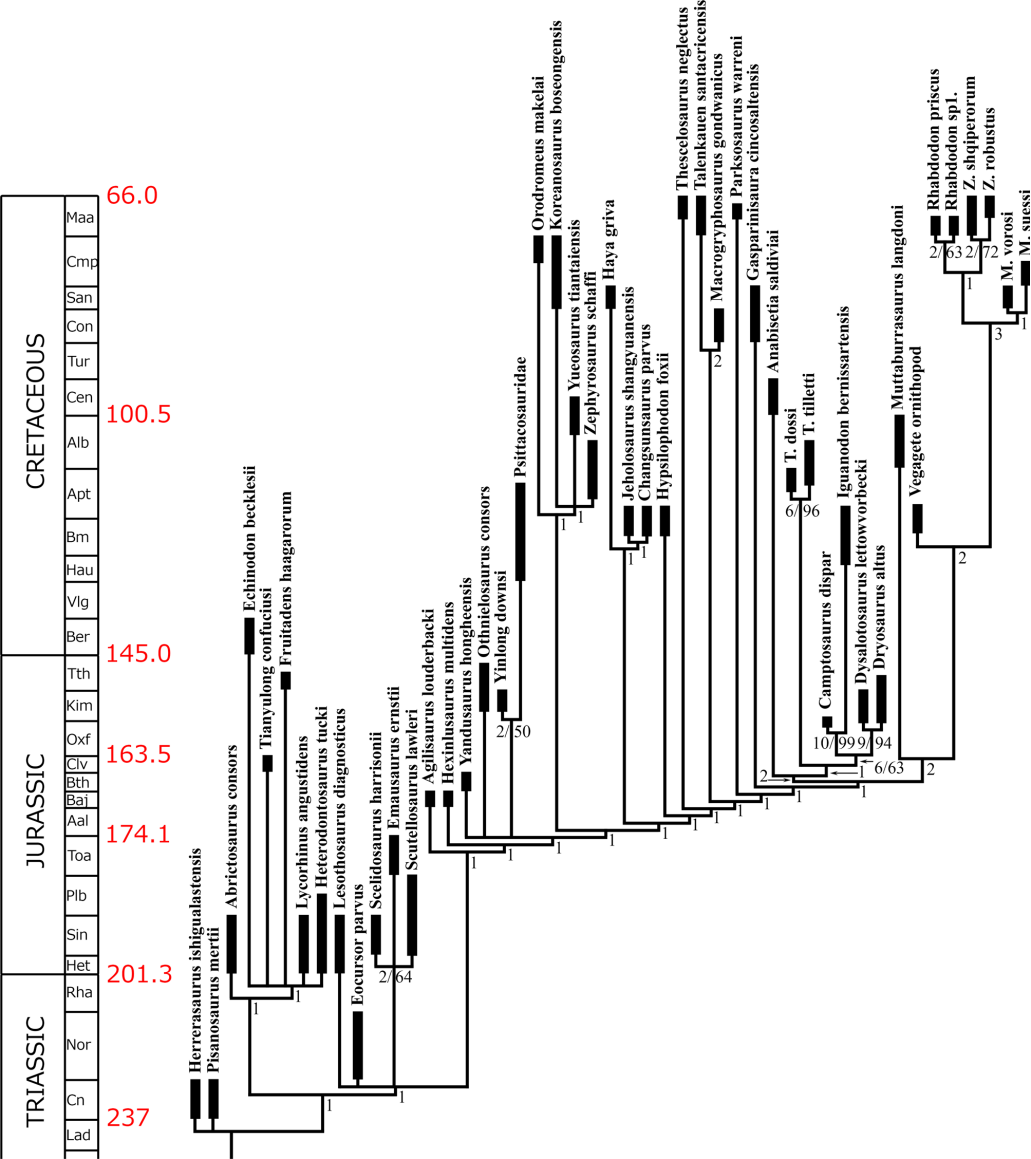
Strict consensus resulting from twenty most parsimonious trees, calibrated over the chronostratigraphic timescale of Gradstein *et al*. [76]. Boostrapping index is reported behind its node only whenever it exceeds 50%. Bremer indices are always reported.

The analysis produced 20 most parsimonious trees with a length of 765 steps (CI = 0.433, RI = 0.636). Major differences are observed between the topology obtained in this analysis (Fig 14) and that obtained in the most recent phylogeny of Boyd (2015). If we consider the two Asian genera *Changsunsaurus* and *Haya* to be part of the Thescelosaurinae (as occurs in [8]), this subfamily becomes polyphyletic. If we omit them from the Parksosauridae, as we will consider here, this subfamily becomes a paraphyletic plexus of forms more derived than *H*. *foxii*. The Orodromiinae are recovered as the sister taxa of an Asian clade comprising *Haya*, *Changsunsaurus* and *Jeholosaurus*; and these two latter clades form a paraphyletic group basal to *Hypsilophodon foxii*. All of these differences observed between our results and those of Boyd [8] may be explained by the fact that row matrices coded differently for the same characters, or focused on different parts of the tree. The present results tries to embrace all of the previous matrices, though it should also be improved with addition of new taxa, and/or an exhaustive revision of coding already done on some OTUs with first hand material observation.

Significant new phylogenetic relationships have been recovered in this paper. Within Iguanodontia, we find a monophyletic group containing Ankylopollexia, the dryosaurids, *Tenontosaurus* and *Anabisetia*. We also find another monophyletic group containing *Muttaburrasaurus* and the Rhabdodontidae (Fig 14). We must say that the latter clade was already recovered one time by McDonald *et al*. [73], but this finding was poorly resolved and not discussed. Relationships between *Muttaburrasaurus* and Rhabodontidae will be dealt in more details herein. The Vegagete taxon is resolved as the most basal member of the Rhabdodontidae. *Zalmoxes* and *Mochlodon* do not form a clade anymore, as was found in a previous analysis [18]. Instead, there is a new clade uniting *Rhabdodon* and *Zalmoxes*, with *Mochlodon* as their new basal sister taxon. This new topology is more congruent with the chronostratigraphy, but it implies a yet unresolved contact between France and Romania during the Maastrichtian.

The Vegagete ornithopod retains many characters that are plesiomorphic for ornithischians. The maxillary teeth crowns are slightly mesiodistally compressed at the base of their cingulum. The crowns of both maxillary and dentary teeth are low, which is a plesiomorphic character according to Weishampel *et al*. [22]. A ventrally convex dentary occurs in the largest individual of the Vegagete ornithopod. This character is found in basal neornithischians such as *Agilisaurus* [77], *Orodromeus* [78], and basal ornithopods such as *Hypsilophodon* [28]. Note that this character is still present in a juvenile specimen of *Mochlodon* but not in the adult [18]. A shallow *fossa trochanteris* is preserved on the Vegagete ornithopod femur (Fig 6A_1_ and 6B_1_). The lesser trochanter does not completely reach the proximal end of the greater trochanter, and this character is observed in more basal ornithischians such as *Lesothosaurus* [55], *Laquintasaura* [79], *Heterodontosaurus* [80] and *Agilisaurus* [77]. Note that this character is also widespread in the Victorian ornithopod femorae [81]).

In accordance with Weishampel *et al*. [22] and the latest conception of Boyd [8], the Vegagete taxon should be rooted within Cerapoda on the basis of the asymmetrical distribution of enamel on its upper and lower teeth [7]. Noteworthy is the fact that the ornithopod *Thescelosaurus neglectus* still possesses equally enameled upper and lower teeth (lingually and labially) [82]. Another unequivocal character indicating that the Vegagete taxon belongs to Cerapoda is the presence of a prominent central ridge on the lingual side of the dentary teeth. This is absent in the neornithischian *Othnielosaurus* [83] but occurs in *Thescelosaurus* and more strongly so in *Hypsilophodon foxii* [28, 84]. The premaxillary tooth morphology of the Vegagete ornithopod is unique and clearly stands out from that of any other premaxillary crowns described to date in any other ornithopod. Unlike the thescelosaurids [85], this crown does not bear a flaring cingulum at its base (Fig 3C). Unlike *H*. *foxii* [28] and the Proctor Lake hypsilophodontid [86], this premaxillary tooth lacks carinae.

**Fig 15:**
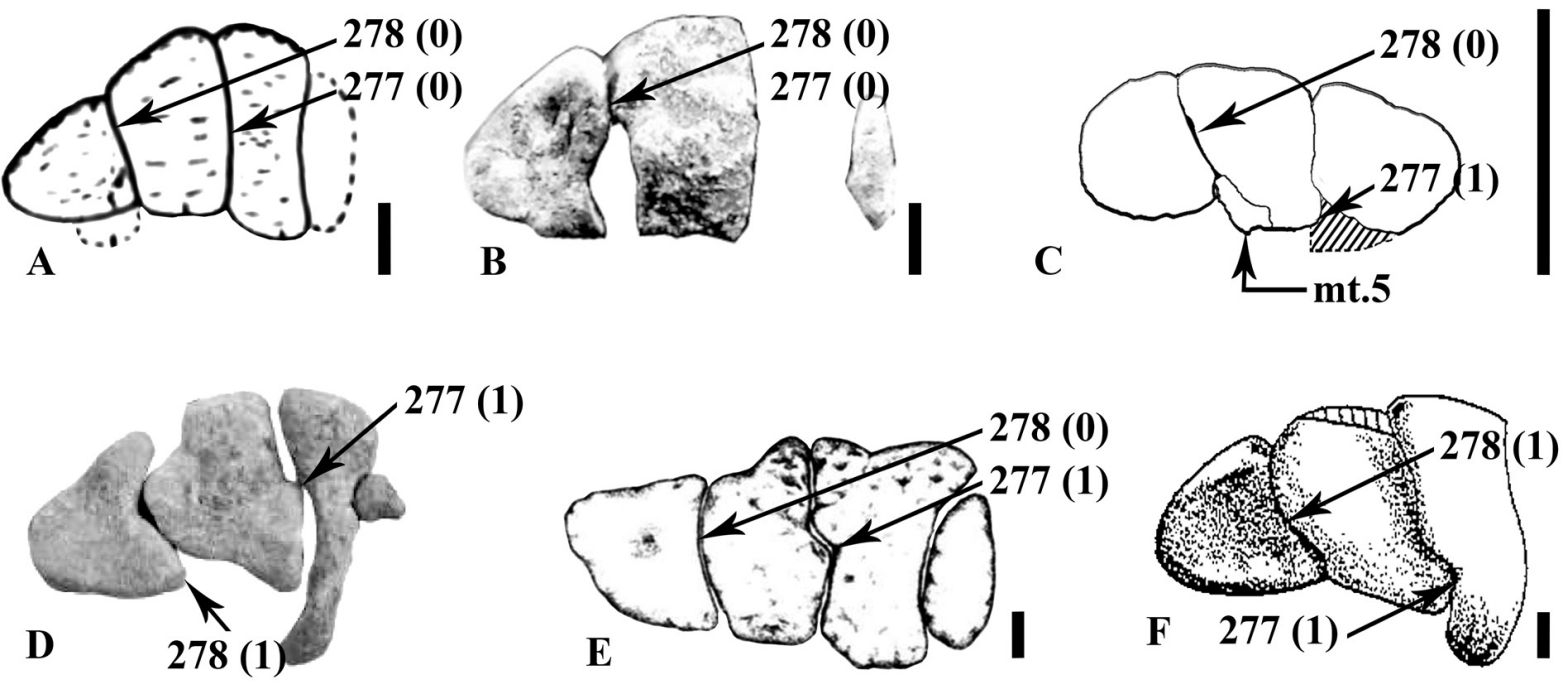
Proximal arrangement of metatarsals for diverse ornithopods in proximal views. (A) *Hypsilophodon foxii* [28]; (B) *Gideonmantellia amosanjuanae* (reversed) [65]; (C) Vegagete taxon; (D) *Anabisetia saldiviai* [53]; (E) *Tenontosaurus tilletti* (reversed) [87]; (F) *Dryosaurus altus*(reversed) [5]. Characters #277 and #278 are shown in states (0) and (1). Abbreviations: mt.5, fifth metatarsal. Scales: 5 mm (A-C), 10 cm (E-F), unknown (D).

Further data suggests the Vegagete taxon is among the most primitive iguanodonts. Firstly, an anterior intercondylar groove is present on the distal portion of its femur. This character is derived and cited as a typical iguanodontian apomorphy [12, 22, 87]. Secondly, the posterior mandible fragment MDS-VG,16/17/152 bears a wide labial emargination (Fig 2C_2_): this derived character is shown by *Zalmoxes robustus* [22], *Dryosaurus altus* [34] and *Mantellisaurus* [88]. Thirdly, a newly added character which relates to the proximal configuration of metatarsals (character #277) seems to be a strong argument for including the Vegagete ornithopod within Iguanodontia. Actually, the second metatarsal overlaps a small medial outgrowth on the proximal extremity of third metatarsal (character #277 (1)). This is observed in all iguanodonts (*Tenontosaurus*, *Dryosaurus altus*, *Anabisetia* (Fig 15D–15F), *Mantellisaurus* [88] and *Ouranosaurus* [89] for example) excepted for *Valdosaurus* [90] and *Gasparinisaura* [91]. In *Iguanodon*, this character is less developed probably because the joint is more complex [92]. Unfortunately, most other publications lack a description or illustration for this character, notably those for rhabdodontids. Regarding *Muttaburrasaurus langdoni*, Bartholomai and Molnar [50] described a second metatarsal that is expanded dorsally, compressed ventrally and “more angular antero-laterally” (i.e., toward the third metatarsal).

Unlike all other studied maxillary teeth, the posterior maxillary tooth of the Vegagete ornithopod (MDS-VG,9, Fig 3E) bears one prominent ridge near the mesial margin of its crown; the posterior margin is partly eroded. In *Muttaburrasaurus* [81, 93] two of the three maxillary teeth described bear a more prominent distal primary ridge, due to the presence of an adjacent sulcus along the posterior margin of the crown. The Argentinean iguanodont *Anabisetia* is the only non-rhabdodontomorphan dinosaur having marked heterodonty: i.e. the association of a spade-like maxillary tooth without prominent apical ridge and non-spade-like maxillary teeth bearing one prominent labial ridge [15, 34]. Concerning the hindlimb, the Vegagete ornithopod shares a plesiomorphic lower-leveled lesser trochanter with respect to the greater trochanter on the femur with all of the Victorian ornithopods [81], the Argentinean *Notohypsilophodon comodorensis* [94], and the South African *Kangnasaurus coetzeei* [47]. This character also occurs in the North American *Othnielosaurus* [59] and the European *Gideonmantellia* [30]. Moreover, the distal medial condyle protrudes anteriorly with respect to the lateral one (character #261 (1)). This occurs in many Gondwanan taxa as *Notohypsilophodon* [94], *Kangnasaurus* [47], *Anabisetia* [34], the Australian specimen VOPCII [58], and *Muttaburrasaurus* [50]. *Kangnasaurus*, *Anabisetia*, and *Muttaburrasaurus* share with the Vegagete taxon the presence of an extensor groove on the distal part of their femora, as well as a medially drawn posterolateral condyle, which is not found in *Notohypsilophodon*. However, *Kangnasaurus* is more “dryosaurid-like,” with the scar for the *M*. *caudifemoralis longus* well separated anteriorly from the fourth trochanter. In *Anabisetia* and *Muttaburrasaurus*, the descriptions and figures do not specify whether the *M*. *caudifemoralis longus* scar merges at the base of the fourth trochanter or is separated from it anteriorly. In the Vegagete material, the *M*. *caudifemoralis longus* scar is proximodistally enlarged and quite anteriorly expanded. We assume that this scar may have been fused to the base of the fourth trochanter, despite the fourth trochanter being broken (Fig 8B). In view of the considerations above, the Vegagete ornithopod presents undisputable Gondwanan affinities.

Concerning the pes, the Vegagete taxon shares with *Gasparinisaura cincosaltensis* [91] a very singular feature, which is a fifth metatarsal placed just between the third and fourth metatarsals (Fig 15C). This feature was previously hardly given any attention, and very few descriptions of the proximal arrangement of metatarsals exist in the literature on basal ornithischian and ornithopods dinosaurs. Taxa such as *Anabisetia saldiviai* [15], *Dryosaurus altus* [5], *Hypsilophodon foxii* [28] and *Heterodontosaurus tucki* [95, 96] present a fifth metatarsal articulated proximally with the beveled posterior surface of distal tarsal 2, 3 or 4, depending on the taxon. The fifth metatarsal shaft is preserved mediodistally across metatarsal IV in *D*. *altus*, and across metatarsal IV to the ventral aspect of metatarsal III in the others. These differences concerning the fifth metatarsal position could be explained by a displacement due to taphonomic processes. Nevertheless, the posterior concave articulation surface observed between metatarsal III and IV in *Gasparinisaura* and in the Vegagete specimen makes the space to receive the fifth metatarsal. Up to now, though such a position is bizarre and rarely reported, it would appear of great interest to look at where the fifth metatarsal is located in other taxa, so we could assess or reject the taxonomical value of this character in the future. We could not reject a possible post-mortem displacement of the fifth metatarsal as the result of tendons relaxing after the death of the individual. To avoid this bias, a new character (#279) has been created to deal with the presence or absence of a proximal concavity between metatarsal III and IV, whether it hosted the fifth metatarsal or not. In the Vegagete ornithopod, the proximo-medial surface of the fourth metatarsal is slightly concave to accommodate a slight convexity on the proximo-lateral surface of the third metatarsal (Fig 15C). However, from a systematic point of view, the appearance of the joint between the third and the fourth metatarsal is roughly flat in appearance, which corresponds to the primitive condition (character #278 (0)) also observed in *Hypsilophodon foxii* [28] and *Othnielosaurus* [57]. By contrast, a protrusive posteromedial process on the proximal part of the fourth metatarsal (character #278 (1)) is clearly observed in the basal iguanodontian *Gasparinisaura* [91], *Valdosaurus* [90] and *Anabisetia* [53]. *G*. *amosanjuanae* is similar to the Vegagete ornithopod in having the distal ligamentary fossae of the first metatarsal anteroposteriorly directed [30]. Herne [58] separately describes a similar condition in the Australian specimen VOPC III. He states (p. 260) that “the distal end of the (first) metatarsal is likely to be plantolaterally”. Consequently and though not described, the orientation of the distal ligamentary fossae of the first metatarsal are likely to be anteroposterior in this case too. *A*. *saldiviai* is also striking in its first pedal digit configuration [15, 53], which looks very similar to that of VOPC III. However the current state of knowledge does not allow us to be more precise. The Vegagete ornithopod still differs from *G*. *amosanjuanae* in that some twisting is observed distally on the first metatarsal of *G*. *amosanjuanae* [33], whereas the signs of such twisting have completely disappeared in the Vegagete ornithopod. It also differs from the VOPC III specimen in that the first metatarsal shaft is cylindrical in VOPC III, whereas it is plate-like in the Vegagete ornithopod.

Among iguanodonts, our taxon would be nested within the Rhabdodontomorpha. This clade was initially referred informally as “Rhabdomorpha” by Pincemaille-Quilleveré [24, 97] to group *Rhabdodon*, *Tenontosaurus*, and *Muttaburrasaurus*. Weishampel *et al*. [98] found that these three taxa form rather an unresolved polytomy with Euiguanodontia (*sensu* Coria and Salgado, 1996 [14]). At that time, “*Rhabdodon*” was used to group the French form *Rhabdodon priscus* [20], the Romanian form *Rhabdodon robustus*, and the Austrian form *Mochlodon suessi*. *M*. *suessi* was thought at that time to be synonymous with *Rhabdodon* [99]. Then, Weishampel *et al*. [22] created the family Rhabdodontidae, including *Rhabdodon priscus* from France and *Rhabdodon robustus* from Romania, the latter being renamed at the same time within the new genus *Zalmoxes* as *Zalmoxes robustus*. In recent work, Ösi *et al*. [18] reassessed the validity of the genus *Mochlodon* [51] and created the new species *M*. *vorosi*. Here, we confirm that the few similarities observed by Pincemaille [24] between *Rhabdodon*, *Muttaburrasaurus*, and *Tenontosaurus* are inaccurate because they were based primarily on plesiomorphic characters. *Muttaburrasaurus* is the basalmost member of a new monophyletic group including the Rhabdodontidae and excluding any relation with *Tenontosaurus*. The phylogeny of McDonald *et al*. [73] was the first to group Rhabdodontidae and *Muttaburrasaurus* within a clade, and we support that result. McDonald *et al*. [73] introduced an interesting character (#64 in this matrix) as an apparent synapomorphy for the rhabdodontomorphan dinosaurs. The anterior process of the jugal overlapping the maxilla with parallel dorsal and ventral margins is present in *Muttaburrasaurus* [50], *Z*. *robustus* [22] and *Z*. *shqiperorum* [23] exclusively. A low dentary tooth count (character #126 (0), fewer or equal to ten dentary teeth) also supported Rhabdodontidae [18]. This character had also been alleged to draw some rhabdodontid taxa closer with the Australian ornithopod *Qantassaurus intrepidus* [81]. By contrast, the Laurasian iguanodonts usually bear a higher dentary tooth count, including *Tenontosaurus dossi* [13], which bears eleven or twelve dentary teeth. Even though we do not know the dentary tooth count of *Muttaburrasaurus*, other taxa such as *Mochlodon*, *Zalmoxes*, *Rhabdodon priscus*, probably the Vegagete taxa, and *Qantassaurus intrepidus* bear up to ten dentary teeth. It is probable that a smaller number of dentary teeth (up to ten) could be symplesiomorphic for Rhabdodontomorpha.

The scapulae of *Muttaburrasaurus langdoni* [50], *Z*. *shqiperorum* [54], *Z*. *robustus* [22], *Mochlodon vorosi* [18], *Rhabdodon* sp.1 [21] and the Vegagete taxon all bear a short posteroventral process just above the glenoid fossa, the posterior edge of which is strongly vertically oriented (character #191 (1)). By contrast, in *T*. *tilleti* and *T*. *dossi* [13, 85], dryosaurids [5, 100] and *Camptosaurus dispar* [101], this posterior process is more divergent and turns to the horizontal posteriorly (character #191 (0)). It is not evident whether character #191 (1) is really a synapomorphy for the clade Rhabdodontomorpha, because it is also present in *Gasparinisaura* [14], *Parksosaurus* [56], *Thescelosaurus* [102], as well as in basal ornithischians such as *Lesothosaurus diagnosticus* [55].

The complete absence of a brevis shelf (character #224 (2)) was recorded only in rhabdodontomorphan dinosaurs, plus *Koreanosaurus* [103]. This is clearly the case, though not being described, in *Z*. *robustus* [22] and *Z*. *shqiperorum* [23] In *Muttaburrasaurus langdoni*, Bartholomai and Molnar [50] mention “the mesial shelf developed toward the base may disappear before reaching the posterior extremity”. However, no brevis shelf was illustrated in their drawing (Fig 9C). The absence of a brevis shelf in *Muttaburrasaurus* is confirmed by Herne (pers. comm.). By contrast, a weak brevis shelf marked by a distinct step was described for *Tenontosaurus tilleti*, which is visible only from a medial view [87]. *Muttaburrasaurus*, *Z*. *shqiperorum* and *Z*. *robustus* share a dorsal margin of the preacetabular process which is transversely expanded to form a narrow shelf (character #219, (1)). *Muttaburrasaurus* (Herne, pers. com.), *Z*. *shqiperorum* and *Z*. *robustus* [22,23] also have an ilium mediolaterally thickened dorsally above the acetabular portion (character #222, (2), (3)). Notwithstanding, we note that the postacetabular process of *M*. *langdoni* thins and ends up as a sharp blade more posteriorly (character #222, (2)). In change, the dorsal thickening of the *Zalmoxes* ilia is maintained and propagates until the post-acetabular process where we observe a strongly everted dorsal margin [22, 23] (character #222, (3)). A dorsal thickening also seems plausible in the postacetabular process of the Vegagete ornithopod, despite its medial margin was broken (Fig 7). A strong lateral deflection of the preacetabular process of ilium is shared exclusively by *Muttaburrasaurus* [50], *Z*. *shqiperorum* and *Z*. *robustus* (character #217 (1)). Though ilia are not available for all of the rhabdodontomorphans and notably for the *Rhabdodon* species referred herein, the shape of this element seems like to be very diagnostic for this group.

**Fig 16:**
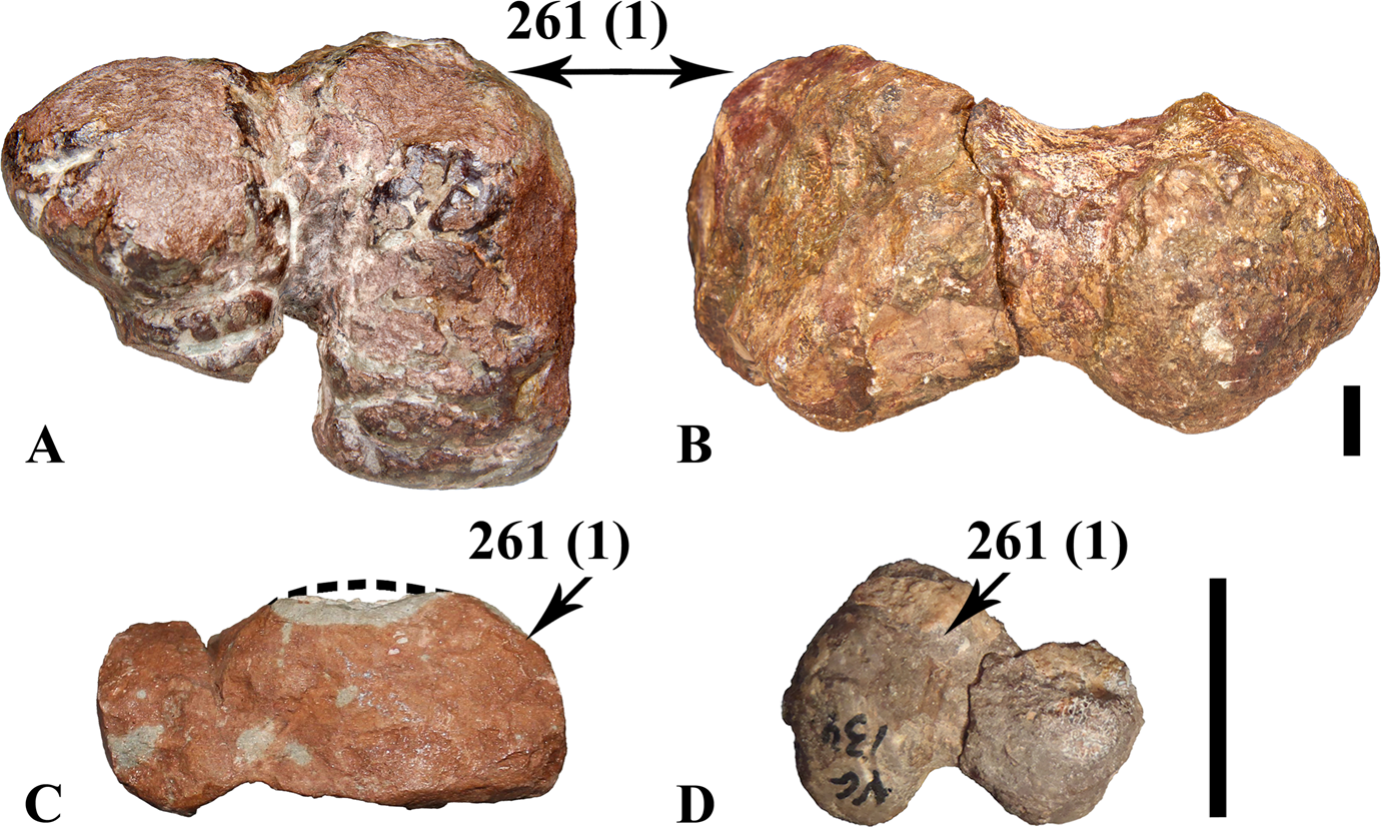
Rhabdodontid femora in distal views. The four distal extremities are right (A, C) and left (B, D). Specimens are (A) *Z*. *robustus* NHMUK 3834; (B) *Z*. *shqiperorum* NHMUK R4900; (C) cf. *Rhabdodon* MHNAIX-PV.2008.1.11; (D) Vegagete ornithopod MDS-VG,134. Character #261 is shown in state (1). Scale: 1cm.

Finally, in the distal extremity of the femora, the anterior protrusion of the medial condyle with respect to the lateral one (character #261) appears to be plesiomorphic for the clade Rhabdodontomorpha. This character was detected in all of the afore-mentioned rhabdodontomorphan genera, including two *Rhabdodon* species: *R*. *priscus* and *R*. sp.1 (Tortosa *et al*., in progress) and the very small individual, *cf*. *Rhabdodon* MHNAIX-PV.2008.1.11 (Tortosa *et al*. in progress) from the neighbouring of Aix-en-Provence, (Fig 16).

**Fig 17:**
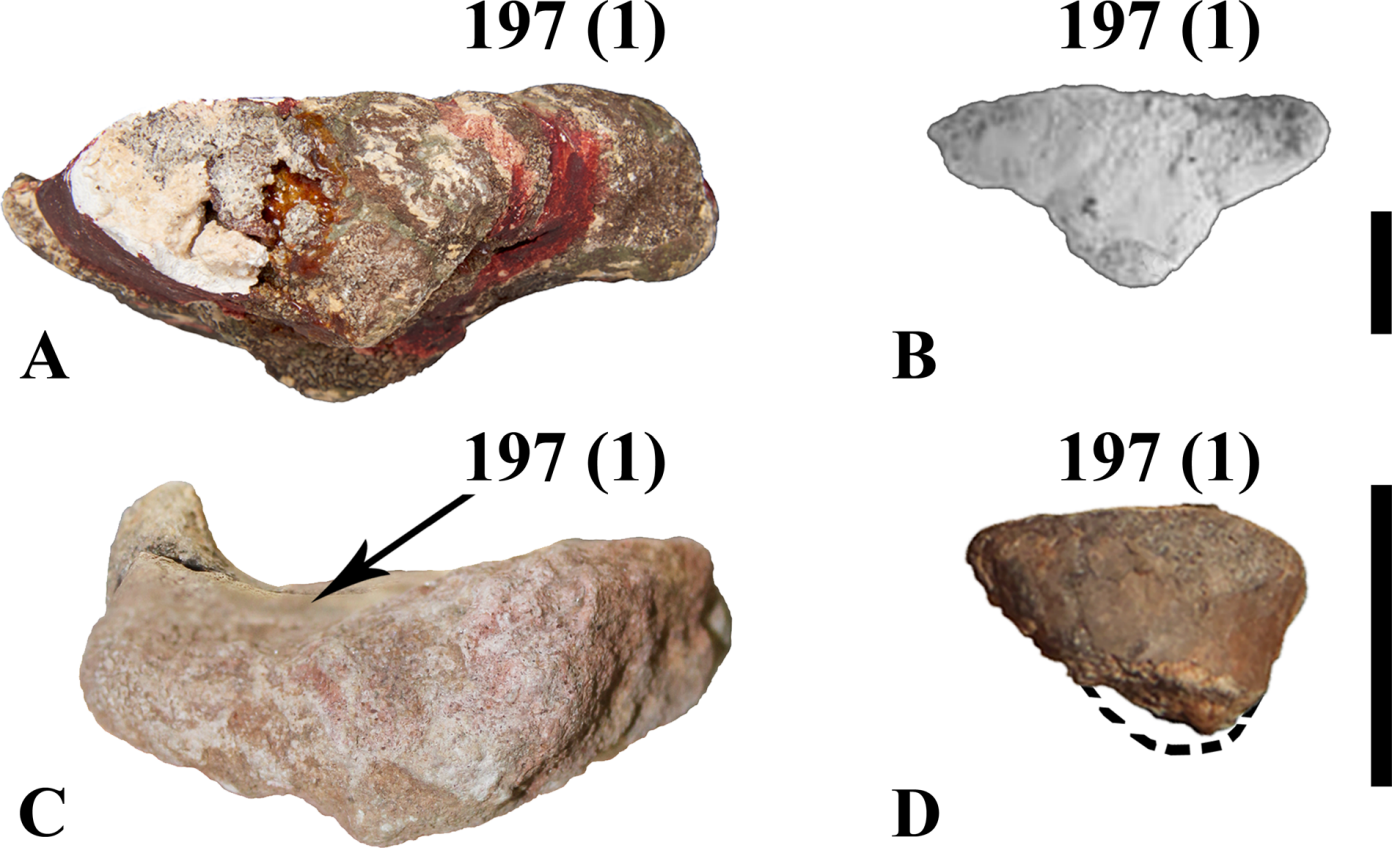
Rhabdodontid humeri in proximal views. The proximal extremities are left (A, C) and right (B, D reversed). Specimens are (A) *Z*. *robustus* NHMUK R3814; (B) *Mochlodon vorosi* MTM-V-2010.128.1 [18]; (C) cf. *Rhabdodon* MHNAIX-PV.2015.13.24; (D) Vegagete ornithopod MDS-VG,113. Character #197 is shown in state (1). The first scale-bar is 1cm for (A-C), the second scale-bar is 1cm for (D).

We reinforce the diagnosis made by Ösi *et al*. [18] for the family Rhabdodontidae, and include the Vegagete ornithopod as a new rhabdodontid. The lateral border between the humerus’ head and deltopectoral crest was found to be concave exclusively in the Vegagete ornithopod, *M*. *vorosi*, *Z*. *shqiperorum* and *Z*. *robustus* (character #198 (1)). Unfortunately, this character is unknown for *Mochlodon suessi* and for *Rhabdodon*. In the Vegagete ornithopod, the proximal head of the humerus displays a flat to smoothly convex anterior side, with no development of any bicipital sulcus (character #197 (1)). To date, this character was only appreciable in *Mochlodon vorosi* (Ösi *et al*. 2012, Fig 6G). This character is also present in *Zalmoxes robustus* (Fig 17). Some isolated humeri: MC-4765 and CM-45 1, [65] and a juvenile specimen MHNAIX-PV.2015.13.24 (Tortosa *et al*., in progress) attributed to cf. *Rhabdodon* confirm the presence of this character (Fig 17). Hence, we consider the absence of proximal bicipital sulcus as a new valid synapomorphy to define the Rhabdodontidae. In *Muttaburrasaurus*, Bartholomai and Molnar [50] mention that the anterior surface of the humerus was nearly flat proximally; however, based on the illustrations, we do not consider it flat enough to code this character as present. Therefore, this character appears to exclude *Muttaburrasaurus* (184 (0)) [47] from the Rhabdodontidae (184 (1)).On the other hand, we found that the bigger Laurasian iguanodonts (e.g. *Dryosaurus altus*, *Dysalotosaurus lettowvorbecki*, *Tenontosaurus tilletti* [5, 87]) and also other primitive ornithopods (e.g. *Hypsilophodon foxii* [28]) contrast in having a much deeper and irregular bicipital sulcus.

Concerning the femur, and among iguanodonts, we code for a non-pendant, crested fourth trochanter ([18] #253 (1)) only and exclusively in rhabdodontids (*Mochlodon*, *Zalmoxes*, and *Rhabdodon* sp.1 from Vitrolles). *Muttaburrasaurus* lies basally with respect to the rhabdodontids, and it has a pendant fourth trochanter as occurs in other Laurasian iguanodonts. Though the fourth trochanter is not preserved in the Vegagete ornithopod, we suggest that a crested, non-pendant fourth trochanter could be synapomorphic for Rhabdodontidae. We find that among iguanodonts, only and exclusively the rhabdodontids diagnosable for this character–i.e. the Vegagete taxon, *Z*. *robustus* and *Z*. *shqiperorum*–have a fully open posterior intercondylar groove on the distal part of their femora (character #259 (0)). However the Rhabdodontomorpha may have borne the plesiomorphical state (1) for this character. Actually *Muttaburrasaurus* does possess a laterally inflated medial condyle posteriorly. This character should have been reversed to state (0) latter in the lineage leading to the Rhabdodontidae. The Vegagete ornithopod shares an exclusive character with *Mochlodon* (Sachs and Hornung, 2005; Ösi *et al*. 2012), which is the presence of a deep muscle insertion on the posterolabial side of the mandibule (# 127 (1)). This character is not observed in the latter two rhabdodontids genera, i.e. *Zalmoxes* (Godefroit *et al*. 2009; Weishampel *et al*. 2003) and *Rhabdodon priscus* (Matheron, 1869).

### Rhabdodontidae origin and palaeobiogeography

Within Rhabdodontidae, the Vegagete taxon is more similar to the Campano-Maastrichtian *Rhabdodon septimanicus* [52] than to any Maastrichtian Transylvanian rhabdodontids (S1 Table) [22, 23], in bearing a tooth row that is curved but not medially offset, and therefore aligned to the coronoid process posteriorly (character #122 (0)). This character could not be safely inferred from the upper Campanian *R*. *priscus* and *R*. sp.1., because in these specimens the tooth row seems parallel with the lateral border of the dentary, which is a condition that differs categorically from that observed in all other rhabdodontids. An interesting discovery [104] concerns a mandibular tooth in the Campanian from Romania. No prominent secondary ridges were observed on this tooth. This was explained as a consequence of the erosion of the enameled surface. However, there remains a possibility that this absence of enamel did not completely mask the morphology of the underlying secondary ridges, so that this specimen would in fact be a great deal closer to the Vegagete ornithopod. Within Rhabdodontidae, the dentary teeth of the Vegagete ornithopod are more similar to those of the Campanian *Rhabdodon* from Laño [19] in that they bear only very few secondary ridges on either side of the very developed primary ridge, which do not reach the base of the crown. Hence, the Vegagete taxon from Lower Cretaceous is the most primitive representative of the European endemic family of Rhabdodontidae [105]. European teeth support the idea of an Early Cretaceous radiation of European rhabdodontids, which would have then evolved in Europe and through the rest of the Cretaceous into many lineages.

Out of Europe, the Vegagete taxon shares much morphology with *Muttaburrasaurus*, and all of the related slender forms of ornithopods already described in Australia [58]. These multiple affinities strongly support a Gondwanan origin of Rhabdodontidae. Notwithstanding, a better understanding of the phylogenetic relationships and systematics of these Southern hemisphere ornithopods is required to refine this statement.

This is not the first time that faunal affinities have been reported between Gondwanan and Western European faunas during the Early Cretaceous. We could cite: 1) the closely related rebbachisaurids *Demandasaurus* from Spain and *Nigersaurus* from Niger [45, 106–108]; 2) the closely related dryosaurids *Valdosaurus* from England and *Elrhazosaurus* from Niger [34, 90]; 3) the baryonychine theropods from Europe and North Africa [109–111]; and 4) the abelisaurid theropod *Genusaurus* from southeastern France [112–114]. The Spanish theropod *Concavenator* and the Nigerian Eocarcharia were found to form a clade at the base of Carcharodontosauridae [115]. As a whole, this faunal similarity points out to a land connection called “the Apulian Route”, implying an active migration of taxa in the Upper Barremian between the southernmost European archipelago and Gondwana [106].

## Conclusions

The ornithopod of Vegagete is a primitive iguanodontian that possesses a remarkable combination of characters, changing our ideas on some previously accepted basal iguanodontian symplesiomorphies, such as the presence of premaxillary teeth. This character was repeatedly lost in at least two lineages: the one leading to Ankylopollexia through *Tenontosaurus*, and the other one leading to Rhabdodontidae. Contrary to what was previously thought, the original radiation of rhabdodontids occurred deep in the past. The discovery of the Vegagete ornithopod pulls the origin of Rhabdodontidae back to the Upper Barremian of Spain. It exemplifies the fact that much of rhabdodontid diversity could still be obscured in the fossil record. The Rhabdodontidae are rooted in a much wider Gondwanan clade that we define here as the new clade Rhabdodontomorpha.

## Supporting Information

S1 Table Matrix of characters. The first row corresponds to the actual character number. The second row corresponds to the previous reference of that character with its corresponding number. MD = McDonald *et al*. 2010; O = Ösi *et al*. 2012; Br = Brown *et al*. 2013; Bd = Boyd, 2015.
(XLSX)

S2 Table Bibliography used for coding and/or emending each taxon’s character. (XLS)

S1 Text New character list used in the present phylogenetical analysis. (DOCX)
