# Supplementary material for: An Unexpected Early Rhabdodontid from Europe (Lower Cretaceous of Salas de los Infantes, Burgos Province, Spain) and a Re-Examination of Basal Iguanodontian Relationships
Source: PLoS One. 2016 Jun 22;11(6):e0156251. doi: 10.1371/journal.pone.0156251 (PMC4917257; doi:10.1371/journal.pone.0156251)
Supplement: S1 Text — (DOCX) [file pone.0156251.s003.docx]

**Supplement 2 -** Characters list

Cranial skeleton

1. Skull, rostral**-**quadrate length relative to the body length: 10 % (0), 13 % or more (1) (Xu *et al.* 2006; Ösi *et al.* 2012 #2).

2. Skull, preorbital skull length relative to the basal skull length: more than 45% (0), less than 40% Ösi *et al.* 2012 #1).

3. Skull, position of maximum widening of the skull: beneath the jugal–postorbital bar (0), posteriorly, beneath the infratemporal fenestra (1) (Ösi *et al.* 2012 #37).

4. Skull, widening of the skull across the jugals, chord from frontal orbital margin to extremity of jugal is more than minimum interorbital width: absent (0), present, skull has a triangular shape in dorsal view (1) (Ösi *et al.* 2012 #36).

5. Skull, cortical remodeling of surface of dermal bone: absent (0), present (1) (Ösi *et al.* 2012 #89).

6. Rostral bone (neomorphic bone anterior to premaxilla): absent (0), present (1) (Ösi *et al.* 2012 #3).

7. Rostral bone, anteriorly keeled and ventrally pointed: absent (0), present (1) (Ösi *et al.* 2012 #4).

8. Rostral bone, ventrolateral processes: rudimentary (0), well-developed (1) (Ösi *et al.* 2012 #5).

9. Premaxilla, anterior and dorsal surface: lacks rugosities (0), bears distinct rugose surface (1) (Brown *et al.*, 2013 #136).

10. Premaxilla, ventral inflection: absent, oral margin even with ventral margin of maxilla (0), present, oral margin projects farther ventrally than ventral margin of maxilla (1) (modified from Norman 2002 #2 ; McDonald *et al.* 2010 #30).

*Hypsilophodon foxii* was corrected and coded (1) following (Galton, 1974a). *Zalmoxes robustus* was corrected and coded (0) following (Weishampel *et al.* 2003, fig. 3E). N.B.: character 9 from (Ösi *et al.* 2012) is similar and was not used in this analysis. However, and consistently with its codification, *Emausaurus ernstii*, the Psittacosauridae and *Yinlong* *downsi* were coded (0), and *Tianyulong* *confuciusi* and *Abrictosaurus* *consors* were coded (1).

11. Premaxilla, denticles on oral margin: absent (0), present (1) (modified from Weishampel *et al.*, 2003 #7; McDonald *et al.* 2010 #33).

12. Premaxilla, edentulous anterior region: absent, first premaxillary tooth is positioned adjacent to the symphysis (0), present, first premaxillary tooth is inset the width of one or more crowns (1) (Ösi *et al.* 2012 #6).

13. Premaxilla, posterolateral process length: does not contact lacrimal (0), contacts the lacrimal, excludes maxilla–nasal contact (1) (Ösi *et al.* 2012 #7).

14. Premaxilla, ventral (or oral) margin: narial portion of the body of the premaxilla slopes steeply from the external naris to the oral margin (0), ventral premaxilla flares laterally to form a partial floor of the narial fossa (1) (Ösi *et al.* 2012 #8).

15. Premaxilla, position of the ventral (or oral) margin: level with the maxillary tooth row (0), deflected ventral to maxillary tooth row (1) (Ösi *et al.* 2012 #9).

16. Premaxilla, premaxillary foramen: absent (0), present (1) (Ösi *et al.* 2012 #10).

17. Premaxilla, premaxillary palate: strongly arched, forming a deep, concave palate (0), horizontal or only gently arched (1) (Ösi *et al.* 2012 #11).

18. Premaxillae: unfused (0), fused (1) (Brown *et al.* 2013 #124).

19. Premaxilla, external naris size: small, entirely overlies the premaxilla (0), enlarged, extends posteriorly to overlie the maxilla (1) (modified from McDonald *et al.* 2010 #38; Ösi *et al.* 2012 #18).

20. Premaxillary border of internal nares present (0), absent (1) (Boyd, 2015 #11).

21. Premaxilla, position of the ventral margin of external nares: below the ventral margin of the orbits (0), above the ventral margin of the orbits (1) (Ösi *et al.* 2012 #17).

22. Premaxilla, narial fossa surrounding external nares on lateral surface of premaxilla, position of ventral margin of fossa relative to the ventral margin of the premaxilla: closely approaches the ventral margin of the premaxilla (0), separated by a broad flat margin from the ventral margin of the premaxilla (1) (Ösi *et al.* 2012 #16).

23. Maximum length of external nares less than 15% basal skull length (0), maximum length of external nares greater than 15% basal skull length (1) (Boyd, 2015 #88)..

24. Premaxilla-nasal contact, overlaping of the dorsal process of the premaxilla onto the nasal: present (0), absent (1) (Ösi *et al.* 2012 #12).

25. Premaxilla-maxilla contact, fossa-like depression positioned on the premaxilla–maxilla boundary: absent (0), present (1) (Ösi *et al.* 2012 #13).

26. Premaxilla-maxilla diastema: absent, maxillary teeth continue to anterior end of maxilla (0), present, substantial diastema of at least one crowns length between maxillary and premaxillary teeth (1) (Ösi *et al.* 2012 #14).

27. Premaxilla-maxilla diastema, form: flat (0), arched ‘subnarial gap’ (1) (Ösi *et al.* 2012 #15).

28. Premaxilla-vomer contact: present (0), absent, excluded by midline contact between maxillae (1) (Ösi *et al.* 2012 #84).

29. Maxilla, prominent anterolateral boss articulates with the medial premaxilla: absent (0), present (1) (Ösi *et al.* 2012 #24).

30. Maxilla, anterior end: exhibits a spike-like process that inserts into the posterior end of the premaxilla (0), bears an anterodorsal sulcus to receive the posterior portion of the premaxilla (1) (modified from: Butler *et al.* 2011; Ösi *et al.* 2012 #25 ; Brown *et al.*, 2013 #40).

*Tenontosaurus dossi* should be corrected and coded (1) (see Winkler *et al.* 1997, description p. 333; Ösi *et al.* 2012 #25).

31. Maxilla, buccal emargination: absent (0), present (1) (Ösi *et al.* 2012 #26).

32. Maxilla, eminence on the rim of the buccal emargination of the maxilla near the junction with the jugal: absent (0), present (1) (Ösi *et al.* 2012 #27).

33. Maxilla, slot for lacrimal: absent (0), present (1) (Ösi *et al.* 2012 #28).

34. External antorbital fenestra, shape: triangular (0), oval or circular (1) (Ösi *et al.* 2012 #22).

35. External antorbital fenestra, exclusion of the jugal from the posteroventral margin by lacrimal–maxilla contact: absent (0), present (1) (Ösi *et al.* 2012 #34).

36. Internal antorbital fenestra, length relative to skull length: large, generally at least 15 % (0), very much reduced, less than 10%, or absent (1) (Ösi *et al.* 2012 #20).

37. Additional opening(s) anteriorly within the antorbital fossa: absent (0), present (1) (Ösi *et al.* 2012 #23).

38. Nasals, deep elliptic fossa present along sutural line of the bones: absent (0), present (1) (Ösi *et al.* 2012 #19).

39. Frontal, contacts orbit: along more than 25% of total frontal length (0), less than 25% (1) (Butler *et al.* 2011; Brown *et al.* 2013 #24).

*Zalmoxes robustus* was coded (1) following illustrations from Weishampel *et al.* 2003.

40. Frontal, ratio of frontal length to nasal length: greater than 120% (0), between 120% and 60% (1) or less than 60% (2) (Brown *et al.* 2013 #25).

41. Frontals: short and broad (0), narrow and elongate (more than twice as long as wide) (1) (Ösi *et al.* 2012 #64).

42. Frontals arched over orbit (0), frontals dorsally flattened (1) (Boyd, 2015 #65).

43. Lacrimal-jugal contact: jugal barely touches lacrimal (0), jugal meets lacrimal with more contact (1), lacrimal-jugal butt joint (2) (Brown *et al.* 2013 #50).

44. Accessory ossification(s) in the orbit (palpebral/supraorbital): absent (0), present (1) (Ösi *et al.* 2012 #29).

45. Palpebral/supraorbital: free, projects into orbit from contact with lacrimal/prefrontal (0), incorporated into orbital margin (1) (Ösi *et al.* 2012 #30).

46. Palpebral, shape in dorsal view: rod-shaped (0), plate-like with wide base (1) (Ösi *et al.* 2012 #31).

47. Palpebral/supraorbital, number: one (0), two (1) or three (2) (Ösi *et al.* 2012 #32).

48. Supraorbital(s) extend across at least 71% of the maximum anteroposterior length of the orbit (0), 70% or less (1) (Boyd, 2015 #25).

49. Lower margin of the orbit circular (0), lower margin of the orbit subrectangular (1) (Boyd, 2015 #95).

Based on the reconstruction of Molnar (1996), *Muttaburrasaurus langdoni* was coded (1).

50. Postorbital, orbital margin: relatively smooth curve (0), prominent and distinct projection into orbit (1) (Butler *et al.* 2011; Ösi *et al.* 2012 #49 ; Brown *et al.*, 2013 #20).

51. Postorbital-parietal contact: absent, very narrow (0), broad (1) (Ösi *et al.* 2012 #51).

52. Squamosal-Quadratojugal contact: present, between dorsal process of quadratojugal and descending process of the squamosal (0), absent (1) (Ösi *et al.* 2012 #52).

53. Supratemporal fenestrae, anteroposteriorly elongated: absent, fenestrae are subcircular to oval in shape (0), present (1) (Ösi *et al.* 2012 #66).

54. Parietosquamosal shelf: absent (0), present (1) (Ösi *et al.* 2012 #68).

55. Postorbital-squamosal tubercle row: absent (0), present (1) (Ösi *et al.* 2012 #72).

56. Postorbital-squamosal tubercle row, enlarged tubercle row on the posterior squamosal: absent (0), present (1) (Ösi *et al.* 2012 #73).

57. Squamosal, morphology of postorbital process dorsal to *M. adductor mandibulae externus* origin site: gently convex (0), mediolaterally compressed and blade-like (1) (McDonald *et al.*, 2010 #65).

58. Jugal, length of the jugal wing on the quadrate: greater than 20% quadrate length (0), less than 20% (1) (Brown *et al.*, 2013 #1).

59. Jugal, ventral extent of the jugal wing ends: at or near distal condyles of quadrate (0), above distal condyles (1), well above the distal condyles (2) (Butler *et al.* 2011; Brown *et al.* 2013 #9).

In *Parksosaurus* (Galton, 1973) and *Thescelosaurus* *neglectus* (Boyd, 2014 to fig. 1A) the jugal wing ends up high enough above the distal quadrate condyles so that they can be coded (2) (instead of (1) previously).

60. Jugal, articulation with quadrate: jugal fails to articulate with quadrate (0), jugal articulates with quadrate (1) (Brown *et al.* 2013 #14).

61. Maxillary process on the medial side of the jugal is medially projected and modestly arched (0), maxillary process on the medial side of the jugal is straight and grooved (1), maxillary process on the medial side of the jugal is anteromedially projected and arched (2) (Boyd, 2015 #39).

*Hypsilophodon* *foxii* was coded (0) accordingly with (Galton, 1974a, Fig. 5C).

62. Jugal, ectopterygoid articular facet on medial view: consists of a deep groove (0), rounded scar (1) (Brown *et al.* 2013 #47).

63. Jugal dorsoventrally deeper than mediolaterally broad (0), broader than deep (1) (Boyd, 2015 #32).

64. Jugal, morphology of portion of maxillary process that overlaps maxilla: tapers at anterior ends of maxillary and lacrimal contacts, with slightly convex ventral margin and slightly concave dorsal margin (0), subrectangular with parallel dorsal and ventral margins (1) dorsoventrally expanded anteriorly (2) or dorsoventrally expanded and deeper than the posterior ramus of the jugal (3) (modified from: McDonald *et al.* 2010 #54; Ösi *et al.* 2012 #35).

Characters tate (2) was not coded in any taxons from this matrix so it was removed. The old character state (4) were combined with the old character state (3), because both seem to originate from a dorsoventrally expanded anterior process of the jugal, whether this anterior process participates to the anterior margin of the orbit or not. This character bears the new state (2). Also, we made a new character state (3) that we picked up from character 35, (2) of Ösi *et al.* (2012). This new state deals with the proportionately larger dorsoventral expansion of the jugal anterior ramus with respect to the posterior one. *Zalmoxes robustus* was corrected and coded (1) following Weishampel *et al.* (2003, p. 74 and fig. 6). *Camptosaurus dispar* (Gilmore, 1909, fig. 2) was corrected and coded (2).Psittacosauridae and *Yinlong* *downsi* were coded (3) following character 35 (2) of Ösi *et al.* (2012).

65. No boss or ornamentation present on lateral surface of the jugal (0), lateral surface of jugal ornamented, but no boss present (1), presence of a low boss on the lateral surface of the jugal (2), presence of a tall, posteriorly projecting boss on the lateral surface of the jugal (3) (Boyd, 2015 #38).

66. Jugal, node-like ornamentation mostly on, or ventral to, the jugal–postorbital bar: absent (0), present (1) (Ösi *et al.* 2012 #41).

67. Jugal-postorbital bar, width broader than infratemporal fenestra: absent (0), present (1) (Ösi *et al.* 2012 #42).

68. Jugal-postorbital joint: elongate scarf joint (0), short butt joint (1) (Ösi *et al.* 2012 #43).

69. Jugal, form of postorbital process: not expanded dorsally (0), dorsal portion of postorbital process expanded posteriorly (1) (Ösi *et al.* 2012 #44).

70. Contact between jugal and postorbital faces anteriorly (0), contact faces partially laterally (1), postorbital inserts into a socket in the jugal (2) (Boyd, 2015 #31).

71. Jugal, forked posterior ramus: absent (0), present (1) (Ösi *et al.* 2012 #46).

72. Jugal, posterior ramus: forms anterior and ventral margin of infratemporal fenestra (0), forms part of posterior margin, expands towards squamosal (1) (Ösi *et al.* 2012 #47).

73. Greatest posterior expanse of the jugal greater than 25% height of skull (0), less than 25% (1) (Boyd, 2015 #26).

74. Jugal–quadratojugal contact: overlapping (0), tongue-and-groove (1) (Ösi *et al.* 2012 #48).

75. Jugal (or jugal–epijugal), ridge dividing the lateral surface of the jugal into two planes: absent (0), present (1) (Ösi *et al.* 2012 #38).

76. Quadratojugal, shape: L-shaped, with elongate anterior process (0), subrectangular with long axis vertical, short, deep anterior process (1) (Ösi *et al.* 2012 #53).

77. Quadratojugal, ventral margin: approaches the mandibular condyle of the quadrate (0), well-removed from the mandibular condyle of the quadrate (1) (Ösi *et al.* 2012 #54).

78. Quadratojugal, quadratojugal foramen: absent (0), present (1) (Weishampel *et al.*, 2003 #17 ; McDonald *et al.*, 2010 #58).

79. Body of the quadrate leans posteriorly (0), body of quadrate oriented vertically (1), body of quadrate leans anteriorly (2) (Boyd, 2015 #47).

80. The pterygoid wing of the quadrate arises at the dorsal head of the quadrate (0), pterygoid wing of the quadrate arises below the dorsal head of the quadrate (1) (Boyd, 2015 #53).

81. Pterygoid wing of quadrate consists of a large, anteromedially extending fan of bone (0), pterygoid wing of the quadrate small (1) (Boyd, 2015 #54). .*Z. shqiperorum* preserves a seemingly massive pterygoid wing though it is incomplete. Therefore it should be corrected (0) following (Godefroit *et al.* 2009).

82. Quadrate, prominent oval fossa on pterygoid ramus: absent (0), present (1) (Ösi *et al.* 2012 #57).

83. Paraquadratic foramen or notch, size: absent or small, opens between quadratojugal and quadrate (0), large, opens inside the quadratojugal (1) (Butler *et al.* 2011; Ösi *et al.* 2012 #60).

*Gasparinisaura* (Coria and Salgado, 1996) and *Dysalotosaurus* (Janensh, 1955) were changed and coded a question mark. *Dryosaurus* (Galton, 1983) needed to be changed and coded (0).

84. Quadrate, mandibular articulation: quadrate condyles subequal in size (0), medial condyle is larger than lateral condyle (1), lateral condyle is larger than medial (2) (Ösi *et al.* 2012 #63).

85. Laterosphenoid, socket for the head: occurs along frontal-postorbital suture (0), only in postorbital (1) (modified from Brown *et al.* 2013 #21).

Following Winkler *et al.* (1997) in *T. dossi* “the capitate process of the laterosphenoid articulates dorsally with the postorbital and dorsomedially with the frontal”. Therefore *T. dossi* should be corrected and coded (0) for this character. In *Dryosaurus altus*, “the transversely expanded dorsal cotylus [of the laterosphenoid] fitted against a surface formed mostly by the frontal and postorbital as in *Hypsilophodon*, *Zephyrosaurus* and *Camptosaurus*” (Galton, 1983). Therefore *D. altus* should be corrected and coded (0) for this character. Codification resulted erroneous as well for basal ankylopollexian. We attest this basing on the following citations on previous publications. In *Camptosaurus dispar* “The alisphenoids are a pair of roughly triangular bones which arise from the anterior dorsal surface of the basisphenoid, and unite dorsally as in the crocodile with the parietal, frontals, and postfrontals” (Gilmore, 1909). We therefore corrected and coded (0) for *Camptosaurus* *dispar*.

86. Opisthotic, presence of a ‘Y-shaped’ indentation on the dorsal edge: absent (0), present (1) (Brown *et al.* 2013 #127).

87. Prootic, position of the foramen for cranial nerve V: notches the anteroventral edge of the prootic (0), nearly or completely enclosed in prootic (1) (Butler *et al.* 2011; Brown *et al.*, 2013 #76). *Zephyrosaurus*’s character state was originally wrong and corrected to (1) (following Sues, 1980).

88. Prootic-basisphenoid plate: absent (0), present (1) (Ösi *et al.* 2012 #81).

89. Supraoccipital, contribution to dorsal margin of the foramen magnum: forms entire dorsal margin of foramen magnum (0), exoccipital with medial process that restricts the contribution of the supraoccipital (1) (Ösi *et al.* 2012 #78).

90. Paroccipital processes (Exoccipital-Opisthotic complex): extend laterally and are slightly dorsoventrally expanded distally (0), distal end pendent and ventrally extending (1) (McDonald *et al.*, 2010 #72 ; modified from Ösi *et al.* 2012 #75)

91. Paroccipital processes, proportions: short and deep (height ≥ 1/2 length) (0), elongate and narrow (1) (Ösi *et al.* 2012 #76).

92. Posttemporal foramen positioned at the boundary between the parietals and the paroccipital process (0), posttemporal foramen positioned entirely within the opisthotic (1), posttemporal foramen positioned entirely within the squamosal (2) (Boyd, 2015 #103).

93. Basioccipital, contribution to the border of the *foramen magnum*: *foramen magnum* occupies over 30% of the width of occipital condyle (0), 20-30% (1), less than 20% of occipital condyle (2) (modified from: Ösi *et al.* 2012 #79; Brown *et al.* 2013 #71).

*Gasparinisaura*’s question mark was corrected to (2) following (Coria and Salgado, 1996). *Haya* was coded (0) following (Makovicky *et al.* 2011). We coded (?) for: *Herrerasaurus*, *Heterodontosaurus*, *Agilisaurus* for which such information was not hold directly or wasn’t verifiable.

94. Basioccipital, orientation of occipital condyle: posteroventrally directed (0), posteriorly

directed (1) (McDonald *et al.* 2010 #74).

*Z. robustus* was corrected and coded (1) following Weishampel *et al.* (2003, fig. 11D).

95. Basioccipital, anteroposteriorly directed groove extending along ventral surface: absent (0), present (1) (McDonald *et al.* 2010 #75).

Weishampel *et al.* (2003) do not report any anteroposteriorly directed groove along the ventral surface of *Zalmoxes robustus*’ basioccipial, we therefore coded (?) for this taxon.

96. Basioccipital, floor of braincase on basioccipital: flat (0), arched (1) (Brown *et al.*, 2013 #72).

97. Basioccipital, median ridge on floor of braincase on the basioccipital: absent (0), present (1) (Brown *et al.* 2013 #73).

98. Basioccipital, anteroposteriorly directed, sharply defined ridge between basal tubera: absent (0), present (1) (McDonald *et al.* 2010 #76).

99. Basioccipital, tubera shape: extend further ventrally than the basisphenoid (0), level (1) (Brown *et al.* 2013 #74).

100. Basioccipital, basal tubera shape: knob-shaped (0), plate-shaped (1) (Ösi *et al.* 2012 #82).

101. Angle between the base and long axis of the braincase greater than 35 degrees (0), angle less than 35 degrees (1) (Boyd, 2015 #98).

102. Basisphenoid, basipterygoid processes orientation: anteroventral (0), ventral (1), posteroventral (2) (Ösi *et al.* 2012 #83).

103. Basisphenoid, length relative to basioccipital length: longer or subequal (0), shorter than

basioccipital (1) (Ösi *et al.* 2012 #80).

*Dryosaurus altus* and *Dysalotosaurus lettowvorbecki* were coded (0) following character #75 from Brown *et al.* (2013).

104. Palatal keel, dorsoventrally deep (deeper than 50% of snout depth) median palatal keel formed of the vomers, pterygoids and palatines: absent (0), present (1) (Ösi *et al.* 2012 #85).

105. Pterygoid-maxilla contact, at posterior end of tooth row: absent (0), present (1) (Ösi *et al.* 2012 #87).

106. Lower jaw, length of post-coronoid elements relative to the total length of the lower jaw: 35-40% (0), 25-35% (1) (modified from Brown *et al.* 2013 #62 and completed with Boyd, 2015, #83).

107. Predentary: absent (0), present (1) (Ösi *et al.* 2012 #90).

108. Predentary, size: short, posterior premaxillary teeth oppose anterior dentary teeth (0), roughly equal in length to the premaxilla, premaxillary teeth only oppose predentary (1) (Ösi *et al.* 2012 #91).

109. Predentary, anterior end in dorsal view: rounded (0), pointed (1) (Ösi *et al.* 2012 #92).

110. Predentary, grooves on either side of midline on anterior surface, extending ventrolaterally to dorsomedially: absent (0), present (1) (McDonald *et al.* 2010 #6).

111. Predentary, oral margin: relatively smooth (0), denticulate (1) (Ösi *et al.* 2012 #93).

112. Predentary, tip of in lateral view: does not project above the main body (0), strongly upturned relative to main body (1) (Ösi *et al.* 2012 #94).

113. Predentary, ventral process: single (0), bilobate (1) (Ösi *et al.* 2012 #95).

114. Predentary, ventral process: present, well-developed (0), very reduced or absent (1) (Ösi *et al.* 2012 #96).

115. Dentary, ratio of dentary height (just anterior to the rising coronoid process) divided by length of dentary: between 15-20% (0), 20-35% (1) (Brown *et al.* 2013 #63).

116. Dentary, symphysis: V-shaped (0), spout shaped (1) (Ösi *et al.* 2012 #97).

117. Dentary, position of the anterior tip: positioned high (0), mid height (1), near lower margin of dentary (2), below lower margin (3) (modified from: Butler *et al.* 2011; Brown *et al.* 2013 #51).

The initial character state for *Jeholosaurus* (Barrett and Han, 2009), *Haya* (Makovicky *et al.* 2011), and *Changsunsaurus* (Jin *et al.* 2010) were changed and turned to (2).

118. Dentary, morphology of ventral margin of anterior ramus leading to the predentary articulation: straight (0), inflected ventrally, such that it curves gently towards the predentary articulation and symphysis (1), curves dorsally towards symphysis (2) (modified from McDonald *et al.* 2010 #16).

*Camptosaurus* *aphanoecetes* (Carpenter and Wilson, 2008, fig. 5A) and *Camptosaurus* *dispar* (Gilmore, 1909, fig.8) should bear characters state (1), so that *C. dispar* was corrected and coded (1).

119. Dentary, tooth row (and edentulous anterior portion) in lateral view: straight (0), anterior end downturned (1) (Ösi *et al.* 2012 #98).

120. Dentary, dorsal and ventral margins: converge anteriorly (0), subparallel (1), deepen anteriorly (2) (McDonald *et al.*, 2010 #15; modified from Ösi *et al.* 2012 #99).

121. Dentary, ventral flange: absent (0), present (1) (Ösi *et al.* 2012 #100).

122. Dentary, orientation of tooth row relative to lateral surface of dentary: convergent anteriorly and posteriorly, bowed medially at mid-length, the tooth row ends posteriorly along the same axis as that of the coronoid process (0), convergent anteriorly and divergent posteriorly so that the tooth row ends medial or slightly medial to the coronoid process longitudinal axis (1) (modified from McDonald *et al.* 2010 #12).

123. Dentary, coronoid process: absent or weak, posterodorsally oblique, depth of mandible at coronoid is less than 140% depth of mandible beneath tooth row (0), well-developed, distinctly elevated, depth of mandible at coronoid is more than 180% depth of mandible beneath tooth row (1) (Ösi *et al.* 2012 #101).

124. Dentary, anterodorsal margin of coronoid process (formed by posterodorsal process): absent (0), present (1) (Ösi *et al.* 2012 #102).

125. Dentary, coronoid process position: posterior to dentition (0), lateral to dentition (1) (Ösi *et al.* 2012 #103).

126. Dentary, number of dentary teeth: 10 or fewer (0), 11–13 (1), 14–17 (2), 18 or more (3) (modified from Weishampel *et al.* 2003: #30; Butler *et al.* 2011; Ösi *et al.* 2012 #228) (ordered character).

If we follow the description from Galton (1983) we record eleven teeth on the dentary of *Dysalotosaurus lettowvorbecki* (state 1) and 14 to 15 in *Dryosaurus altus* (state 2). *Camptosaurus dispar* was described by Gilmore (1909) as bearing 14 to 16 teeth so this taxon was coded (2), instead of the previous unknown character state.

127. Dentary, the posterolateral surface bears a profound circular depression: absent (0), present (1) (modified from Ösi *et al.* 2012 #230).

N.B.: We modified this character to emphasize the importance of the circular depression. For example, a posterolateral depression is actually present onto the posterolateral surface of the *Tenontosaurus* *tilletti* dentary (Thomas, 2015, figure 46), though it does not have nothing comparable with the depression observed onto the posterolateral surface of the *Mochlodon* dentaries (Ösi *et al.* 2012, fig. 3E, 3G; Sachs and Hornung, 2005, fig. 2.1).

128. External mandibular fenestra, situated on dentary-surangular-angular boundary: present (0), absent (1) (Ösi *et al.* 2012 #104).

129. Retroarticular process: elongate (0), rudimentary or absent (1) (Ösi *et al.* 2012 #107).

130. Dentary-angular, node-like ornamentation: absent (0), present (1) (Ösi *et al.* 2012 #108).

131. Jaw, level of jaw joint: level with tooth row, or weakly depressed ventrally (0), strongly depressed ventrally, more than 40% of the height of the quadrate is below the level of the maxilla (1) (Ösi *et al.* 2012 #109).

132. Premaxillary teeth: present (0), absent, premaxilla edentulous (1) (Ösi *et al.* 2012 #111).

133. Premaxillary teeth, crown expanded above root: crown is unexpanded mesiodistally above root, no distinction between root and crown is observable (0), crown is at least moderately expanded above root (1) (Ösi *et al.* 2012 #113).

134. Premaxillary teeth increase in size posteriorly: absent, all premaxillary teeth subequal in size (0), present, posterior premaxillary teeth are significantly larger in size than anterior teeth (1) (Ösi *et al.* 2012 #114).

135. Teeth, crown is mesiodistally expanded above root in cheek teeth: absent (0), present (1) (Ösi *et al.* 2012 #129).

136. Teeth, close-packing and quicker replacement eliminating spaces between alveolar border and crowns of adjacent functional teeth: absent (0), present (1) (Ösi *et al.* 2012 #131).

137. Teeth, wear facets on teeth: absent or sporadically developed (0), systematic development of wear facets along the entire tooth row (1) (Ösi *et al.* 2012 #222).

138. Maxillary/dentary teeth, position of maximum apicobasal crown height in tooth rows: anterior portion of tooth row (0), central portion of tooth rows (1), posterior portion of tooth rows (2) (Ösi *et al.* 2012 #130).

139. Maxillary/dentary teeth, marginal ornamentations: fine serrations set at right angles to the margin of the tooth (0), coarse serrations (denticles) angle upwards at 45 degrees from the margin of the tooth (1) (Ösi *et al.* 2012 #116).

140. Maxillary/dentary teeth, enamel symmetrical (0), asymmetrical (1) (Ösi *et al.* 2012 #117).

141. Maxillary/dentary teeth, apicobasally extending primary and secondary ridges: absent (0), present (1) (modified from Butler *et al.* 2011; Ösi *et al.* 2012 #118).

142. Maxillary/dentary teeth, apicobasally extending ridges on labial/lingual surfaces of crowns confluent with marginal denticles: absent (0), present (1) (Ösi *et al.* 2012 #119).

143. Maxillary/dentary teeth, at least moderately developed labiolingual expansion of crown (‘cingulum’): present (0), absent (1) (Ösi *et al.* 2012 #123).

144. Maxillary teeth, overlapping of adjacent crowns in maxillary teeth: non-packed maxillary teeth (0), lack of space between adjacent maxillary teeth up through the occlusional margin (1) (modified from: Brown *et al.*, 2013 #31; Ösi *et al.* 2012 #128; McDonald *et al.*, 2010 #88).

*Agilisaurus* was modified and coded (1). The inset of maxillary teeth for this taxon is clearly seen in (Barret *et al.* 2005, fig. 2).

145. Maxillary/dentary alveolar foramina (‘special foramina’) medial to tooth rows: present (0), absent (1) (Ösi *et al.* 2012 #126).

146. Maxillary teeth, crown shape: lingually concave (0), lingually convex (1) (Brown *et al.*, 2013 #37).

147. Maxillary teeth, crowns shape: relatively low spade-like, rectangular, or triangular (0), high diamond-shaped maxillary tooth crowns (1) (Brown *et al.* 2013 #41).

148. Maxillary teeth, apical ridge position, centrally placed (0), posteriorly set (1) (Brown *et al.* 2013 #38).

149. Maxillary teeth, prominent primary ridge on labial side: absent (0), present (1) (Ösi *et al.* 2012 #120).

150. Maxillary teeth, number and morphology of ridges on labial surface of crown: primary ridge with multiple parallel accessory ridges on either side (0), multiple ridges of similar size, such that primary ridge cannot be distinguished (1) (modified from McDonald *et al.* 2010 #92).

151. Maxillary teeth, root shape: straight (0), curved (1) (Brown *et al.* 2013 #33; Boyd, 2015 #119).

152. Dentary dentition, heterodonty: no substantial heterodonty is present in dentary dentition (0), single, enlarged, caniform anterior dentary tooth, crown is not mesiodistally expanded above root (1), anterior dentary teeth are strongly recurved and caniform, but have crowns expanded mesiodistally above their roots and are not enlarged relative to other dentary teeth (2) (Ösi *et al.* 2012 #124).

153. Dentary teeth, peg-like tooth located anteriorly within dentary lacks denticles, strongly reduced in size: absent (0), present (1) (Ösi *et al.* 2012 #125).

154. Dentary teeth, intercrown spaces: present (0), absent (1) (You *et al.* 2003b #32; McDonald *et al.* 2010 #80).

155 Dentary teeth, crown shape: rectangular, triangular, or leaf-shaped (0), lozenge-shaped (1) (Brown *et al.* 2013 #60).

156. Dentary teeth, apical ridge position: anteriorly or centrally positioned (0), posteriorly positioned (1) (Brown *et al.* 2013 #52).

157. Dentary teeth, number of ridges on crown: fewer than 10 (0), more than 10, and often more than 17 (1) (Weishampel *et al.* 2003 #32; Ösi *et al.* 2012 #229).

158. Dentary teeth, prominent primary ridge on lingual side: absent (0), present (1) (Ösi *et al.* 2012 #121).

159. Dentary teeth, number and morphology of ridges on lingual surface of crown: prominent primary ridge and multiple separate faint accessory ridges on both sides of it (0), prominent primary ridge and multiple evenly-spaced accessory ridges on either side such that entire lingual surface is corrugated (1), parallel and similarly prominent primary and secondary ridges with multiple faint accessory ridges arising from marginal denticles (2) (McDonald *et al.*, 2010 #87) (ordered character).

Following Galton (1983, fig. 5F, H) and Janensh (1955, fig. 16A), *D. altus* and *D. lettowvorbecki* were corrected and coded (2) for this character.

160. Ridges present on both sides of dentary crowns (0), ridges limited to one side of dentary crowns (1) (Boyd, 2015 #124).

N.B.: Basing on respectively Weishampel *et al.* (2003), Matheron (1869) and Quilleveré (2002) it is not possible to know wether *Zamoxes* *robustus*, *Rhabdodon* *priscus* and *Rhabdodon* sp. from Vitrolles had some ridges on the labial side of their dentary crowns or not. Therefore they were corrected and coded with a question mark. In change, *Z. shqiperorum* displays well some ridges onto the labial side of its crowns (cf. Godefroit *et al.* 2009, Fig. 11B) so it was corrected and coded (0) for this character.

161. Dentary teeth, root shape in cross-section: round (0), oval (1), squared (2) (modified from Butler *et al.* 2011; Brown *et al.* 2013 #58).

162. Dentary tooth roots straight in anterior or posterior view (0), dentary tooth roots curved in anterior or posterior view (1) (Boyd, 2015 #135).

Axial skeleton:

163. Postaxial cervical vertebrae, epipophyses on anterior cervicals: present (0), absent (1) (Ösi *et al.* 2012 #133).

164. Cervical vertebrae (4-9), form of central surfaces: amphicoelous (0), at least slightly opisthocoelous (1) (Ösi *et al.* 2012 #134).

165. Ventral surface of the cervical vertebrae rounded (0), presence of a broad, flattened keel on the ventral surface of the cervical vertebrae (1), presence of a sharp ventral keel on the ventral surface of the cervical vertebrae (2) (Boyd, 2015 #143).166. Anterior cervical centra less than 1.5 times longer than tall (0), length of anterior cervical centra equal

or greater than 1.5 times longer than tall (1) (Boyd, 2015 #144).

N.B.: *Camptosaurus* *dispar* and *Iguanodon* *bernissartensis* could be coded (1) based on Gilmore (1909, fig. 11.1) and Norman (1980, fig. 26.a) respectively.

167. Cervical vertebrae, number: 7/8 (0), 9 (1), 10 or more (2) (Ösi *et al.* 2012 #135) (ordered character).

168. Dorsal vertebrae, number: 12–13 (0), 14-15 (1), 16 or more (2) (Ösi *et al.* 2012 #137) (character personally modified, ordered character). Here we include the dorsal vertebrae count of 14 and 15 together into the character state (1), as no character state was designed for a dorsal vertebrae count of 14 previously.

N.B.: *Camptosaurus* *dispar* was coded (1), as it actually bears 15 dorsal vertebrae and the sacrodorsal doesn’t count for the dorsal vertebrae number.

169. Dorsal vertebrae, neural spine: anteriorly positioned or centered over the dorsal centrum (0), posteriorly positioned (1) (Butler *et al.* 2011; Brown *et al.* 2013 #78).

*Dryosaurus altus* (Galton, 1981) and *Dysalotosaurus lettowvorbecki* (Janensh, 1955) were corrected and coded (0) for this character, as in both, the post-zygapophyzes do not expand further posteriorly than the proper centra

170. Sacrum composed of three or fewer fused vertebral centra (0), sacrum composed of between four and five fused vertebral centra (1), sacrum composed of six fused vertebral centra (2), sacrum composed of seven or more fused vertebra centra (3) (Boyd, 2015 #148).

171. Sacral vertebrae, neural spines height: less than 2 times the height of the centrum (0), neural spines between 2 and 2,5 times the height of the centrum (1), greater than 2,5 times (2) (Brown *et al.* 2013 #82).

172. Sacral vertebrae, neural spines orientation: lean posteriorly (0), slightly anteriorly (1) (Brown *et al.* 2013 #83).

173. Sacrum, accessory articulation with pubis: pubis does not articulate with the sacrum (0), pubis supported by sacral rib (1), pubis supported by sacral centrum (2) (modified from: Ösi *et al.* 2012 #139 ; Brown *et al.* 2013 #84).

Character #139 of Ösi *et al.*  (2012) was similar though more imprecise, as it dealt with the mere presence (1) or absence (0) of “sacrum” articulation with the pubis. It is worthy to note that some conflicts exist regarding the coding of *Tenontosaurus*. in both matrices. *T. tilletti* was coded (1) in (Ösi *et al.* 2012), whereas *Tenontosaurus* was coded (0) in (Brown *et al.* 2013). We kept the former character state (1) for *T. tilletti*, basing on Foster (1990, p.285 and fig. 15): “The scarred ventral portion of the medial iliac body indicates that the first sacral rib attached at the base of and all along the pubic peduncle [of the ilium…]”. We consider that, indeed, an articulation with the base of the pubic peduncle of the ilium is enough for coding (1) here. As well, *Thescelosaurus neglectus* was coded (0) in Brown *et al.* (2013), but it was corrected to (1), as occurs in Ösi *et al.* (2012) and by considering Galton (1974b, fig. 3P).

174. Ischiac peduncle of the ilium is not supported by a sacral rib (0), ischiac peduncle of the ilium supported by a sacral rib (1) (boyd, 2015 #190).

175. Caudal vertebrae, neural spine position: caudal neural spines positioned over centrum (0), extend beyond own centrum (1) (Brown *et al.* 2013 #88).

176. Anterior caudal vertebrae, neural spines: height the same or up to 50% taller than the centrum (0), more than 50% taller than the centrum (1) (Ösi *et al.* 2012 #142).

177. Dorsal ribs, transition between a near vertical orientation of the *tuberculum* and *capitulum* to a horizontal orientation: occurs within ribs 2-4 (0), 5-6 (1), 6-8 (2) (Brown *et al.* 2013 #79).

178. Anterior dorsal ribs, distal portions of the shaft in cross-section: circular or oval (0), highly laterally compressed with concave lateral and rugose posterior surfaces (1) (Brown *et al.* 2013 #135).

179. Partial ossification of the sternal segments of the cranial dorsal ribs absent (0), present (1) (Boyd, 2015 #157; Ösi *et al.* 2012 #145).

180. Caudal ribs, location: borne on centrum (0), on neurocentral suture (1), on neural arch (2) (Brown *et al.* 2013 #85).

181. Caudal ribs, longest rib position: the first caudal vertebra bears longest rib (0), longest rib posterior to the first (1) (Brown *et al.* 2013 #87).

182. Chevrons, shape: rod-shaped, often with slight distal expansion (0), strongly asymmetrically expanded distally, width greater than length in mid caudals (1) (Ösi *et al.* 2012 #144).

183. Ossified epaxial tendons along vertebral column: absent (0), present (1) (Ösi *et al.* 2012 #216).

184. Ossified epaxial tendons, arrangement: longitudinally arranged (0), double-layered lattice (1) (modified from Ösi *et al.* 2012 #218).

185. Ossified hypaxial tendons, on caudal vertebrae: absent (0), present (1) (Brown *et al.* 2013 #86; Ösi *et al.* 2012 #217).

Appendicular skeleton:

186. Ossified clavicles: absent (0), present (1) (Ösi *et al.* 2012 #147).

187. Scapula-Humerus, proportions: scapula longer or subequal to the humerus (0), humerus substantially longer than the scapula (1) (Ösi *et al.* 2012 #149).

188. Scapula, blade-shape: strongly expanded distally (0), weakly expanded, near parallel-sided (1) (Ösi *et al.* 2012 #152).

189. Scapula, scapular blade length relative to minimum width: relatively short and broad, length is 5-8 times minimum width (0), elongate and strap-like, length is at least 9 times the minimum width (1) (Ösi *et al.* 2012 #150).

190. Scapula, acromion shape: weakly developed or absent (0), well-developed spine-like (1) (Ösi *et al.* 2012 #151).

191 Scapula, proximo-distal corner above the glenoid cavity: elongated, so that the distal edge turns to the horizontal posteriorly (0), forms a more obtuse angle, the distal edge is steep and does not expand posteriorly (1) (New character).

192. Coracoid, width divided by length: less than 60% (0), between 70% and 100% (1) or greater than 100% (2) (Brown *et al.* 2013 #90).

193. Coracoid, coracoid foramen position: enclosed within coracoid (0), open along coracoid-scapula suture (1) (Brown *et al.* 2013 #91).

194. Coracoid, development of the sternal process: short and broad (0), extremely elongated and narrow (ratio greater than 0.80) (1) (Weishampel *et al.* 2003: # 44 ; Ösi *et al.* 2012 #231).

195. Sternal plates, shape: absent (0), kidney-shaped (1), shafted or hatchet-shaped (rod-like posterolateral process, expanded anterior end) (2) (Ösi *et al.* 2012 #148).

196. Humerus, length relative to femoral length: more than 60% (0), less than 60% (1) (Ösi *et al.* 2012 #153).

197. Humerus, appearance of the anterior surface in proximal view: a varyingly developed flexor bicipital sulcus is visible (0), the anterior surface is completely straight to smoothly convex, no bicipital sulcus visible (1) (New character).

198. Humerus, proximal end in anterior/posterior view, lateral border between head and deltopectoral crest: straight or gently convex (0), concave (1) (Ösi *et al.* 2012 #232).

199. Humerus, head separated from prominent medial tubercle on proximal surface by a groove: absent (0), present (1) (Ösi *et al.* 2012 #223).

200. Humerus, deltopectoral crest development: well-developed, projects anteriorly as a distinct flange (0), rudimentary, is at most a thickening on the anterolateral margin of the humerus (1) (Ösi *et al.* 2012 #154).

201. Humerus, deltopectoral crest shape: distal margin rounded and merges gradually with the lateral margin of the humeral shaft (0), distal margin angular and merges abruptly with the lateral margin of the humeral shaft (1) (modified from Weishampel *et al.* 1993#37 ; McDonald *et al.*, 2010 #103). Following Weishampel *et al.* (2003; fig. 20A, B) we corrected and coded *Z. robustus* with character state (1).

202. Humerus, shaft form in anterior or posterior view: relatively straight (0), strongly bowed laterally along length (1) (Ösi *et al.* 2012 #155).

203. Ulna, olecranon process: low (0), moderately developed (1), relatively high (2) (Butler *et al.* 2011; Brown *et al.* 2013 #93).

In *Dryosaurus altus*, the olecranon process of the ulna is low (0) (Galton, 1981). In *Tenontosaurus* *dossi* and *tilletti*, it seems moderately developed (1) (Winkler *et al.* 1997, Forster, 1990). For *Rhabdodon* sp.1, the state is unknown (Pincemaille-Quilleveré, 2002).

204. Ulna, distal end: directed ventrally in medial or lateral view (0), curves gently posteriorly in medial or lateral view (1) (Ösi *et al.* 2012 #233).

205. Carpus, fusion: unfused (0), fused (1) (Brown *et al.* 2013 #97).

206. Metacarpals, block-like proximal ends: absent (0), present (1) (Ösi *et al.* 2012 #157).

207. Metacarpals I and V: substantially shorter in length than metacarpal III (0), subequal in length to metacarpal III (1) (Ösi *et al.* 2012 #158).

208. Metacarpal I greater than 50% the length of metacarpal II (0), metacarpal I less than 50% the length of metacarpal II (1) (Boyd, 2015 #174).

209. Metacarpal/manual phalanges, extensor pits on the dorsal surface of the distal end: absent or poorly developed (0), deep, well-developed (1) (Ösi *et al.* 2012 #162).

210. Manual phalanx, the longest length relative to humerus length: less than 10% (0), more than 15% (1) (Ösi *et al.* 2012 #156).

211. Penultimate phalanx of fingers II and III: shorter than first phalanx (0), longer than the first phalanx (1) (Ösi *et al.* 2012 #159).

212. Manual digits II–IV: First phalanx relatively short compared to second phalanx (0), first phalanx more than twice the length of the second phalanx (1) (Ösi *et al.* 2012 #161).

213. Manual digit III, number of phalanges: 4 (0), 3 or fewer (1) (Ösi *et al.* 2012 #160).

214. Manual unguals, strongly recurved with prominent flexor tubercle: absent (0), present (1) (Ösi *et al.* 2012 #163).

215. Ilium, preacetabular process shape and length: short, tab-shaped, distal end is posterior to pubic peduncle (0), elongate, strap-shaped, distal end is anterior to pubic peduncle (1) (Ösi *et al.* 2012 #165).

216. Ilium, preacetabular process length relative to the ilium length: less than 50% (0), more than 50% (1) (Ösi *et al.* 2012 #166).

217. Ilium, lateral deflection of the preacetabular process: 10°-20° degrees from midline (0), more than 30° (1) (Ösi *et al.* 2012 #167).

218. Ilium, dorsal margin of preacetabular process and dorsal margin above acetabulum: narrow, not transversely expanded (0), dorsal margin is transversely expanded to form a narrow shelf (1) (Ösi *et al.* 2012 #168).

219. Ilium, preacetabular process expands mediolaterally towards its distal end in dorsal view: absent (0), present (1) (Ösi *et al.* 2012 #169).

220. Ilium, supra-acetabular ‘crest’ or ‘flange’: present (0), absent (1) (Ösi *et al.* 2012 #176).

221. Ilium, postacetabular process: posteriorly directed (0), process curves dorsally along its entire length, such that the both the dorsal and ventral margins curve dorsally (1) (McDonald *et al.* 2010 #114; Ösi *et al.* 2012 #170).

222. Ilium, morphology of dorsal margin of postacetabular process dorsal to ischial peduncle: smooth surface (0), laterally bulging eminence dorsal to ischial peduncle, no modification of dorsal margin (1), mediolaterally thickened dorsal margin compared to dorsal margin above pubic peduncle (2) or thickened and laterally-bulging everted rim along dorsal margin (3) (modified from: Norman, 2002 #56; McDonald *et al.* 2010 #112) (ordered character).

Following Shepherd *et al.* (1977), we corrected and coded (1) for *Dryosaurus altus* and *Dysalotosaurus lettowvorbecki*. We can’t infer this character in *Talenkauen santacrucensis* so it was corrected and coded a question mark here, instead of (1) previously.

223. Ilium, postacetabular process shape in lateral view: rounded with break in slope along dorsal margin, parallel dorsal and ventral margins (0), tapers to a point with break in slope along dorsal margin, forming a distinct platform for the origin of *M. iliocaudalis* (1), tapers with no break in slope along dorsal margin (2) (modified from Norman, 2002 #57; McDonald *et al.* 2010 #113) (ordered character).

We could not infer this character in *Thescelosaurus* *neglectus* (Gilmore, 1915; Galton, 1974b) so this character was set to a question mark instead of (0) previously.

224. Ilium, brevis shelf and fossa: fossa faces ventrolaterally and shelf is near vertical and visible in lateral view along entire length, creating a deep postacetabular portion (0), fossa faces ventrally and posterior portion of the shelf cannot be seen in lateral view (1), no brevis shelf distinguishable from both lateral and medial views (2) (modified from Butler *et al.* 2011; Ösi *et al.* 2012 #173).

There is apparently no post-acetabular brevis shelf in the ilia of *Zalmoxes robustus,* *Z. shqiperorum*, and *Muttaburrasaurus langdoni*. This character is unknown in *Rhabdodon* sp.1 from Vitrolles (Pincemaille-Quilleveré, 2002). These informations are alleged uniquely from the figures of Weishampel *et al.* 2003 (Fig. 22C), Godefroit *et al.* 2009 (Fig. 18B) and Herne (personal communication, 2015) respectively. Interestingly, *Koreanosaurus boseongensis* (Huh *et al.*, 2010) was also reported to be devoid of any brevis shelf.

225. Ilium, length of the postacetabular process relative to the total length of the ilium: 0. 20% or less (0), 25-35% (1), more than 35% (2) (Ösi *et al.* 2012 #174) (ordered character).

226. Ilium, medioventral acetabular flange, partially closing the acetabulum: present (0), absent (1) (Ösi *et al.* 2012 #175).

227. Ilium, pubic peduncle: large, elongate, robust (0), reduced in size, shorter in length than ischial peduncle (1) (Ösi *et al.* 2012 #178).

228. Ilium, ischial peduncle: projects ventrally (0), broadly swollen, projects ventrolaterally (1) (Butler *et al.* 2011; Ösi *et al.* 2012 #177).

In *Zalmoxes* *robustus*, it cannot be firmly stated that the ischial peduncle of the ilium is just projecting ventrally, as it was described as being “enormous” and “lenticular” (Weishampel *et al.* 2003). We therefore coded it as unknown for this taxon. On the other hand, *Z. shqiperorum* (Godefroit *et al.* 2009) is explicitly described to bear a much more laterally everted ischial peduncle on its ilium. We replaced its former character state (0) by character state (1).

229. Acetabulum on ilium normal to high (0), acetabulum on ilium short to long (1) (Boyd, 2015 #182).

230. Pubis, massive and dorsolaterally rotated body obscuring the obturator foramen in lateral view: absent (0), present (1) (Ösi *et al.* 2012 #191).

231. Pubis, orientation: anteroventral (0), rotated posteroventrally to lie alongside the ischium (opisthopubic) (1) (Ösi *et al.* 2012 #186).

232. Pubis, prepubic process: absent (0), present (1) (Ösi *et al.* 2012 #192).

233. Pubis, prepubic process shape: compressed mediolaterally, dorsoventral height exceeds mediolateral width (0), rod-like, mediolateral width exceeds dorsoventral height (1), dorsoventrally compressed (2) (modified from: Butler *et al.* 2011; Ösi *et al.* 2012 #193).

234. Pubis, prepubic process length: stub-like and poorly developed, extends only a short distance anterior to the pubic peduncle of the ilium (0), elongated into distinct anterior process (1) (Ösi *et al.* 2012 #194).

235. Pubis, prepubic process extending beyond distal end of preacetabular process of ilium: absent (0), present (1) (Ösi *et al.* 2012 #195).

236. Angle between prepubic process and pubic shaft greater than 150 degrees (0), angle less than 100 degrees (1) (Boyd, 2015 #196).

237. Pubis, pubic symphysis extending: elongate (0), restricted to distal end of pubic blade, or absent (1) (Ösi *et al.* 2012 #196).

238. Pubis, shape of the postpubis shaft in cross-section: blade-shaped (0), rod-shaped (1) (Ösi *et al.* 2012 #187).

239. Pubis, length of postpubis shaft relative to ischium length: approximately equal (0), extends for around half the length (1), very short to absent (2) (modified from: McDonald *et al.*, 2010 #117 ; Ösi *et al.* 2012 #188, #189).

240. Ischium, pubic peduncle shape: transversely compressed (0), dorsoventrally compressed (1) (Ösi *et al.* 2012 #179).

The pubic peduncle of the ischium appears more transversely compressed than dorsoventrally compressed in *Thescelosaurus neglectus* (Gilmore, 1915) but such a description was never provided in detail. This character was corrected to a question mark in this taxon, instead of (1) previously.

241. Pubic peduncle of ischium larger than iliac peduncle (0), peduncles subequal or iliac peduncle larger than pubic peduncle (1) (Boyd, 2015 #200).

242. Ischium, groove on the dorsal margin: absent (0), present (1) (Ösi *et al.* 2012 #183).

243. Ischium, tab-shaped obturator process: absent, lacks an obturator process (0), present and placed 60% down the shaft of ischium (1), placed 50% down the shaft (2) or placed within the first proximal 40% of the shaft (3) (modified from: Butler *et al.* 2011; Ösi *et al.* 2012 #184; Brown *et al.* 2013 #102). *Gasparinisaura* is corrected to a question mark as its ischium is incomplete distally (Coria and Salgado, 1996). *Camptosaurus* *dispar* and *Iguanodon* *bernissartensis* are corrected and coded (3) (Carpenter and Wilson, 2008 and Norman, 1980 respectively).

244. Ischium, morphology of shaft: distally curved (0), proximally curved (1), straight (2) (McDonald *et al.* 2010 #119).

245. Ischium, shaft in cross-section: compressed mediolaterally (0), subcircular and bar-like (1) (Ösi *et al.* 2012 #181).

246. Ischium, distal shaft expansion: expands weakly, or is parallel-sided, distally (0), distally expanded into a distinct ‘foot’ (1) (Butler *et al.* 2011; modified from Ösi *et al.* 2012 #182).

*Gasparinisaura* is corrected and coded with a question mark as its distal ischial shaft is not preserved (Coria and Salgado, 1996).

247. Ischium, symphysis length: median symphysis with the opposing blade along at least 50% of its length (0), symphysis only presents distally (1) (Ösi *et al.* 2012 #185).

248. Femur, shape in medial/lateral view: bowed anteriorly along length (0), straight (1) (McDonald *et al.* 2010 #121; Ösi *et al.* 2012 #197).

249. Femur, femoral head: confluent with greater trochanter, *fossa trochanteris* is smooth and groove-like (0), *fossa trochanteris* is modified into distinct constriction separating head and greater trochanter (1) (Ösi *et al.* 2012 #198).

*Mochlodon vorosi* (Ösi *et al.* 2012) and *Iguanodon* *bernissartensis* (Norman, 1980) were

corrected and coded (0). Character is unknown for *C. dispar* (Gilmore, 1909).

250. Anterior trochanter below or slightly below the level of the greater trochanter (0), levels the greater trochanter (1) or is higher (2) (modified from: Butler *et al.* 2011 and Boyd, 2015 #215; inspired by Ösi *et al.* 2012 #200).

Many characters are ontogenetically controlled in the femorae and for such reason, should not be taken into account. The depth of the intertrochanteric notch has been observed to vary within ontogeny in *Tenontosaurus* (e.g. Forster, 1990), and this is what occurs in the Vegagete ornithopod too, between the subadult and the adult individuals. We could not reject the possibility that this separation could vary between sexes of the same species. Therefore it is dangerous to consider the degree of separation of the lesser trochanter. By measure of precaution, we preferred not to speak about the degree of separation of the anterior trochanter in the modification brought here. *Heterodontosaurus tucki* (Santa Lucas, 1980), *Othnielosaurus* (Galton and Jensen, 1973 see plate 3), *Jeholosaurus* (Xu *et al.* 2000), *Haya* (Makovicky *et al.* 2011), *Koreanosaurus* (Huh *et al.* 2011) and *Camptosaurus* *dispar* (Gilmore, 1909) were corrected and coded (0). *Orodromeus makelai* (Scheetz, 1999, fig. 27) also seems to have a slightly lower lesser trochanter with respect to the greater one. It was corrected to character state (0). Interestingly, in *Zalmoxes robustus* the anterior trochanter almost reaches the proximal end of the greater trochanter (Weishampel *et al.* 2003) but it is clearly one step below the dorsal margin of the greater trochanter. In *Zalmoxes shqiperorum* (Godefroit *et al.* 2009 see Fig. 19) the proximalmost extent of such lesser trochanter is well below the dorsal part of the greater trochanter. Therefore both *Zalmoxes* taxa were coded (0). We could not determine firmly this character for *Thescelosaurus neglectus* (Gilmore, 1915, fig. 12) and for *Fruitadens haagarorum* (Butler *et al.* 2009), so they were coded (?).

251. Lesser trochanter of femur positioned anterior and medial to greater trochanter (0), lesser trochanter positioned anterior and somewhat lateral to greater trochanter (1) (Boyd, 2015 #216).

252. Lateral surface of the greater trochanter of femur convex (0), lateral surface of the greater trochanter flattened (1) (Boyd, 2015 #213).

N.B.: *Muttaburrasaurus* *langdoni* was corrected and coded (1) owing to Bartholomai and Molnar (1981, fig. 9F).

253. Femur, fourth trochanter shape: low eminence or absent (0), prominent ridge (1), pendent (2) (modified from: Butler *et al.* 2011; Ösi *et al.* 2012 #201).

Here we consider *Zalmoxes* *robustus* to bear a non-pendent fourth trochanter (Weishampel *et al.* 2003, fig. 23). The specimen UBB NVZ9 of *Zalmoxes* *shqiperorum*, in turn, was told fortuitously to bear a “pendent” fourth trochanter by Godefroit *et al.* (2009). However, illustrations do not support such affirmation because the fourth trochanter was broken. On the other hand, another specimen from the same species: BMNH R4900 does show a crest-like non-pendent fourth trochanter (see Weishampel *et al.* 2003, fig. 32). Therefore, we coded (1) for *Z. shqiperorum*. *Mochlodon suessi* (Sachs and Hornung, 2005 to fig. 4) and *Mochlodon vorosi* (Ösi *et al.* 2012) also shows a clearly crested, non-pendent fourth trochanter. We therefore changed their codification and turned it to character state (1).

254. Femur, fourth trochanter position: located entirely on proximal half of femur (0) or positioned at mid-length, or distal to mid-length (1) (Ösi *et al.* 2012 #202).

255. Femur, pendent fourth trochanter, rod-like with subparallel anterior and posterior surfaces: absent (0), present (1) (Ösi *et al.* 2012 #224).

256. Femur, location of insertion scar of *M. caudifemoralis longus*: extends from fourth trochanter onto medial surface of femoral shaft (0), widely separated from fourth trochanter, restricted to medial surface of femoral shaft (1) (Ruiz-Omeñaca *et al.* 2007; McDonald *et al.*, 2010 #125).

257. Femur, anterior (extensor) intercondylar groove on distal end: absent (0), present (1) (Ösi *et al.* 2012 #203).

258. Femur, anterior (extensor) intercondylar groove: shallow open through with sides that diverge from each other cranially (0), deep open through with parallel sides (1) (modified from: Norman 2002 #64; McDonald *et al.* 2010 #127).

*Z. shqiperorum* was corrected and coded (0), following the specimen NHMUK R4900 in

Weishampel *et al.* (2003, fig. 32A).

259. Femur, posterior (flexor) intercondylar groove: fully open (0), medial condyle inflated laterally, partially covers opening of flexor groove (1) (modified from: Butler *et al.* 2011; Ösi *et al.* 2012 #204).

*Zalmoxes robustus* and *Z. shqiperorum* had never figured their femorae in distal view. Based

on photos from NHMUK 3834 (*Zalmoxes robustus*) and NHMUK R4900 (*Z. shqiperorum*), we coded both species with character state (0).

260. Femur, posterolateral condyle position and size in ventral view: positioned relatively laterally and slightly narrower in width than the medial condyle (0), strongly inset medially, reduced in width relative to medial condyle (1) (modified from: Butler *et al.* 2011; Ösi *et al.* 2012 #205). *Mochlodon vorosi* (Ösi *et al.* 2012, Fig. 7), *Zalmoxes robustus* (Weishampel *et al.* 2003, fig. 23), and *Zalmoxes Shqiperorum* (Godefroit *et al.* 2009, fig. 19) were coded (1) instead of the previously unknown character state (Ösi *et al.* 2012, #205).

261. Femur, cranial expansion of medial condyle: equal to, or less than lateral condyle (0), protrudes cranially to lateral condyle, and continues onto the cranial surface as a diaphyseal ridge to cranial trochanter (1) (Herne *et al.* 2013 #233).

262. Tibia, relative size of proximal inner and external (or “fibular) condyles: both the inner and external condyles are equal in size (0), external condyle shorter antero-posteriorly, its posterior extremity is more anteriorly set than that of the inner condyle (1), external condyle very short to absent, posterior half of the fibula’s proximal head lays against the inner condyle directly (2) (modified from Butler *et al.* 2011; Brown *et al.*, 2013 #112) (ordered character). Perhaps that in *Rhabdodon* sp.1 (Pincemaille-Quilleveré, 2002, fig. 19.1) and *Z. shqiperorum* (Godefroit *et al.* 2009 to Fig. 20A) the fibula had fitted against a strongly reduced fibular condyle antero-posteriorly (character state 2), so that the posterior part of its head would have been lead over the proximal inner condyle of the tibia. However without direct view of the articulation with the fibula, this could not be asserted and these taxa keep an unknown character state pending further verification. By contrast, it seems that in *Hypsilophodon* *foxii* (Galton, 1974a, fig. 56E), *Dryosaurus altus* (Galton, 1981, fig. 16), *Dysalotosaurus lettowvorbecki* (Janensh, 1955), *Tenontosaurus tilleti* (Forster, 1990) and *Camptosaurus aphanoecetes* (Carpenter and Wilson, 2008, fig. 31A, 31E) the external condyle was anteroposteriorly reduced, but still preventing the contact of the fibular head onto the inner condyle (character state 1).

263. Tibia, mid-shaft in cross-section: triangular (0), round (1) (Brown *et al.* 2013 #114).

264. Tibia, distal shape: subquadrate, posterolateral process not substantially developed (0), elongate posterolateral process, backing fibula (1) (Ösi *et al.* 2012 #206).

265. Tibia, maximum expansion of distal end relative to proximal: distal end is considerably less expanded than proximal (0), maximum expansion of distal end is subequal to that of proximal end (1) (Ösi *et al.* 2012 #227).

266. Fibula, shaft in cross-section: elliptical or round (0), D-shaped (1) (Brown *et al.* 2013 #115).

267. Fibula, distal end is strongly reduced and splint-like: absent (0), present (1) (Ösi *et al.* 2012 #225).

268. Astragalus/calcaneum, indistinguishable and fused to one another: absent (0), present (1) (Ösi *et al.* 2012 #226).

269. Astragalus, anterior side size: high (0), moderate (1), low (2) (Brown *et al.* 2013 #118).

270. Astragalus, posterior side size: low (0), high (1) (Brown *et al.*, 2013 #117).

271. Astragalus, fibular facet on the lateral margin of the proximal surface: large (0), reduced to small articulation (1) (Ösi *et al.* 2012 #207).

272. Calcaneum, proximal surface: facet for tibia absent (0), well-developed facet for tibia (1) (Ösi *et al.* 2012 #208).

273. Calcaneum, angle between the tibial and fibular articular facets: greater than 120 degrees (0), less than 120 degrees (1) (Brown *et al.*, 2013 #119).

274. Medial distal tarsal, shape: blocky in dorsal view (0), thin and rectangular (1), round (2) (Brown *et al.*, 2013 #120).

275. Medial distal tarsal: articulates distally with metatarsal III only (0), articulates distally with metatarsals II and III (1) (Ösi *et al.* 2012 #209).

276. Lateral distal tarsal, shape: square in dorsal view (0), kidney-shaped (1) (Brown *et al.* 2013 #122).

277. Metatarsal II/metatarsal III, morphology of the contact in proximal view: continuous and rather flat (0), metatarsal II overlaps a proximal outgrowth on the ventro-medial side of the metatarsal III (1) (New character).

278. Metatarsal IV, pronounced postero-medial expansion on the most proximal extremity, covering at least part of the posterior side of metatarsal III: absent (0), present (1) (New character).

279. Metatarsal III and IV, tightly adpressed proximally, no concavity is observed posteriorly between them from a proximal view (0), conspicuous concavity to either, or both, the posterolateral side of metatarsal III and the posteromedial side of metatarsal IV which can eventually host the fifth metatarsal (1) (New character).

280. Metatarsal V, length relative to metatarsal III length: more than 50% (0), less than 25% (1) (Ösi *et al.* 2012 #213).

281. Metatarsal V: bears digits (0), lacks digits (1) (Ösi *et al.* 2012 #214).

282. Pes, number of functional digits (i.e., bear phalanges): four (0), three (1) (Brown *et al.*, 2013 #123).

283. Pedal digit I, configuration: robust and well-developed metatarsal I, distal end of phalanx I-1 projecting beyond the distal end of metatarsal II (0), reduced metatarsal I, proximally splint like, end of phalanx I-1 not extending beyond the end of metatarsal II (1), reduced metatarsal I in a vestigal splint or absent, not bearing digits (2) (Ösi *et al.* 2012 #211).

284. Pedal unguals, shape: tapering, narrow pointed, claw-like (0), wide, blunt, hoof-like (1) (Ösi *et al.* 2012 #215).

Dermal skeleton

285. Mandibular osteoderm: absent (0), present (1) (Ösi *et al.* 2012 #110).

286. Dermal osteoderms, parasagittal row on the dorsum of the body: absent (0), present (1) (Ösi *et al.* 2012 #219).

287. Dermal osteoderms, lateral row of keeled dermal osteoderms on the dorsum of the body: absent (0), present (1) (Ösi *et al.* 2012 #220).

288. Dermal osteoderms, U-shaped cervical/pectoral collars composed of contiguous keeled osteoderms: absent (0), present (1) (Ösi *et al.* 2012 #221).
